# Supplementary material for: Emx2 underlies the development and evolution of marsupial gliding membranes
Source: Nature. 2024 Apr 24;629(8010):127–35. doi: 10.1038/s41586-024-07305-3 (PMC11062917; doi:10.1038/s41586-024-07305-3)
Supplement: Supplementary file 1 — Supplementary Notes 1–4, Supplementary Figs. 1–6, Supplementary Tables 1–5 and Supplementary References. [file 41586_2024_7305_MOESM1_ESM.pdf]

nature portfolio

https://doi.org/10.1038/s41586-024-07305-3

Supplementary information

Emx2 underlies the development and evolution of marsupial gliding membranes

In the format provided by the authors and unedited

Supplementary Notes

Supplementary Note 1.

Our experiments indicate that activating *Emx2* and *Wnt5a* in mouse skin can trigger similar phenotypes to those observed during early patagium differentiation. This finding suggests that the *Emx2-Wnt5a* axis may not only be crucial for regulating patagium formation in sugar gliders but may also represent a conserved mechanism for patterning other tissues and structures in developing mammalian embryos. To explore this possibility, we obtained publicly available highly resolved spatial transcriptomics datasets from multiple stages of laboratory mouse embryos with near-single-cell resolution (i.e., Stereo-seq[93]) and examined whether *Emx2* and *Wnt5a* were co-expressed in other developing tissues. In agreement with previous studies, analysis from the forebrain of E14.5 embryos showed that *Emx2* and *Wnt5a* were co-expressed in the same cells (**Supplementary Fig. 1a,b**). This result was further validated through *in situ* hybridizations (**Supplementary Fig. 1c**). Moreover, we observed robust co-expression of *Emx2* and *Wnt5a* in the craniofacial mesenchyme of E14.5 embryo samples, particularly within the spatial domain originally annotated as "jaw and tooth" (**Supplementary Fig. 1d**)[93]. Notably, *Emx2* expression showed spatial heterogeneity, while *Wnt5a* was more widespread (**Supplementary Fig. 1e,f**). To more precisely determine the cellular identities of Stereo-seq spots co-expressing *Emx2* and *Wnt5a*, we employed SpaceFlow[94] to create spatially coherent clusters. These clusters were formed by grouping spots based on both gene expression similarity and spatial proximity (**Supplementary Fig. 1g**). Differential gene expression analysis between spots containing high *Emx2* and *Wnt5a* ($Emx2^{high}$/$Wnt5a^{high}$) and the remaining spots, revealed genes involved in craniofacial bone and tooth formation, including *Osr1*, *Trps1* and *Msx1*[95-97]. Thus, these results indicate that, in addition to patterning the forebrain, the *Emx2-Wnt5a* axis likely coordinates osteoblast fate commitment in the embryonic craniofacial mesenchyme.

Supplementary Note 2.

In addition to the lack of regulatory sequence conservation, the inability of GARs to drive reporter expression in laboratory mice may be explained by differences in the trans regulatory environment between marsupials and laboratory mice. To gain insights into this, we reanalyzed a scRNA-seq data set from E12.5-E14.5 laboratory mouse skin[74] to determine whether mouse orthologs of any of the 13 patagium-upregulated transcription factors predicted to bind to the regulatory motifs enriched in glider species were co-expressed in the same cells as *Emx2*. Among the different cell types identified in E12.5 laboratory mouse skin, *Emx2* was specifically expressed in dermal fibroblasts (**Supplementary Fig. 2a**). Analysis of reclustered fibroblasts revealed that only 5 of the 13 transcription factors (*Maz*, *Rara*, *Sp3*, *Vezf1*, and *Znf740*) were expressed in the same subcluster of dermal fibroblasts as *Emx2* (**Supplementary Fig. 2b**). Within this subcluster, we observed very little overlap between cells expressing these transcription factors and *Emx2*, as indicated by co-expression analysis (**Supplementary Fig. 2c**). Thus, while the upstream factors controlling *Emx2* expression in marsupial gliders remain unknown, the results from this analysis point to potential differences in the trans regulatory environment between marsupial gliders and laboratory mice.

Supplementary Note 3.

We used *in vitro* assays to study the functional significance of multiple GARs and found evidence suggesting regulatory convergence around the *Emx2* locus in gliding species. Both the *P. volans* and the *A. pygmaeus* GARs exhibited significantly higher activity than the orthologous sequences of their corresponding non-glider species, indicating potential key functional differences in *Emx2* expression between these glider species and their non-glider counterparts. The challenge of accessing and sampling tissue for *P. volans* and *A. pygmaeus* at the relevant stages, however, makes it difficult to confirm whether *Emx2* is expressed at high levels in the developing patagia of these two species. The *P. breviceps* GAR also induced a robust luciferase reporter activity, like the non-glider, *D. trivergata*. This could be because this GAR is located within the *Emx2* promoter, and both species require *Emx2* expression for proper tissue patterning. Although we didn't find differences in transcription factor binding sites between the two sequences, we cannot rule out the possibility that species-specific differences in trans-regulatory factors may differentially influence promoter activity between the two species.

Supplementary Note 4

During brain development, *Emx2* forms a regulatory loop with *Wnt5a* and other Wnt ligands that controls cell proliferation and promotes expansion of the cerebral cortex[98]. In *Emx*$^{-/-}$ mutants, expression of *Wnt5a* is considerably reduced, causing progenitors to exit the cell cycle prematurely. As a result, mutants have a marked reduction in the size of the occipital cortex and hippocampus[98]. Notably, *in vivo* downregulation of *Emx2* in the patagium was accompanied by a significant decrease in *Wnt5a*. Moreover, our mouse experiments show that upregulation of both *Wnt5a* and *Emx2* led, among other cellular phenotypes, to increases in cell proliferation. Thus, it is likely that some of the mechanisms by which *Emx2* and *Wnt5a* interact to direct cellular proliferation and pattern the cerebral cortex function in skin to regulate patagium development.

Supplementary Figures

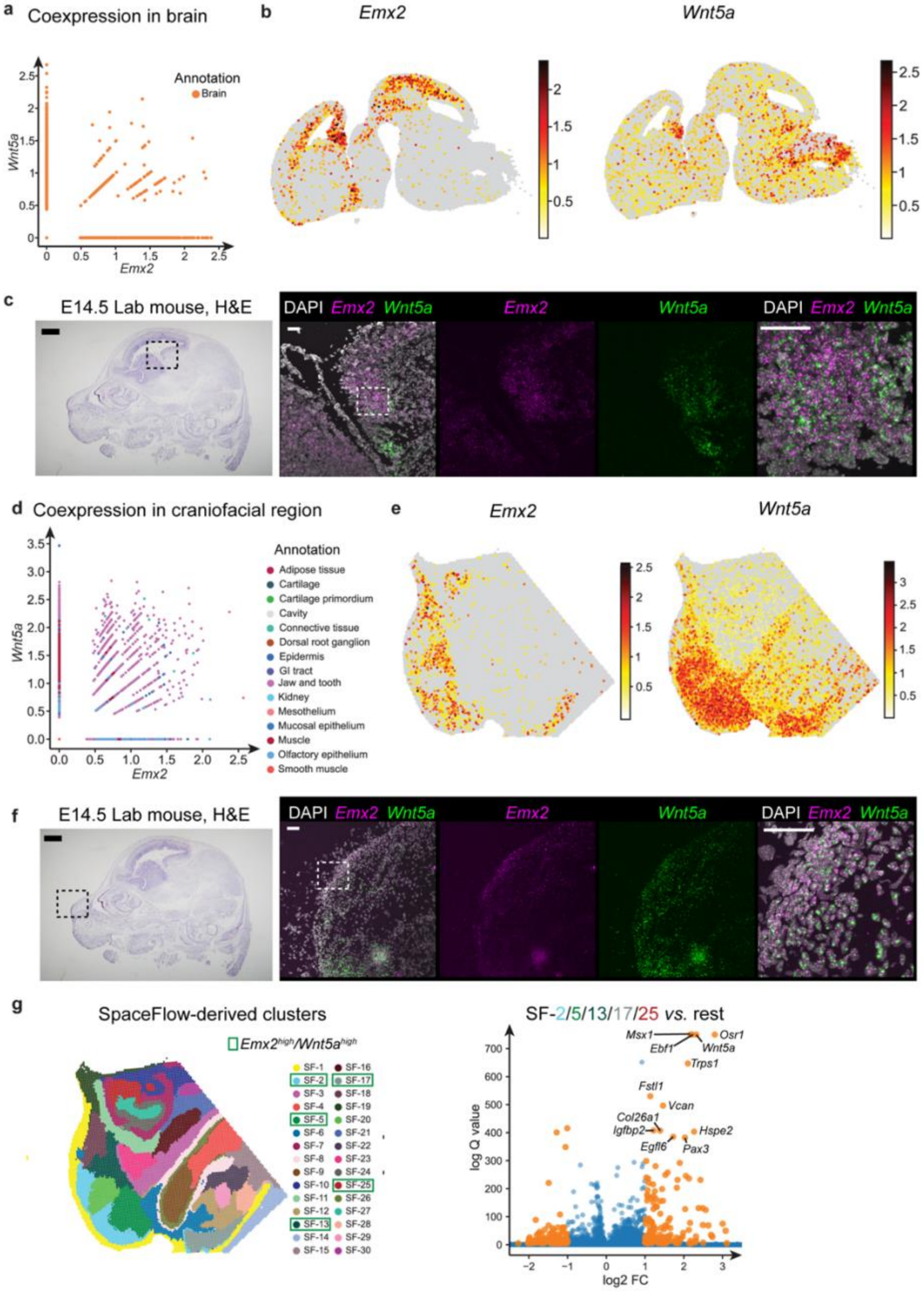

Supplementary Fig. 1. *Emx2* and *Wnt5a* co-expression analysis. a-g, Analysis of the E14.5 E1S1 sample showing co-expression of *Emx2* and *Wnt5a* in the brain (**a**) and in the craniofacial region (**d**), where spots are colored by their original annotation. **b, e,** Spatial expression of *Emx2* (left panel) and *Wnt5a* (right panel) in the E1S1 sample. **c, f,** *In situ* hybridization chain reaction for *Emx2* (magenta) and *Wnt5a* (green) in E14.5 mouse embryo forebrain (hippocampus region) (**c**) and mouse rostra (**f**). **g,** Spatial clusters identified by SpaceFlow (left panel) and differentially expressed genes for *Emx2*^{high}/*Wnt5a*^{high} spatial cluster SF-2, SF-5, SF-13, SF-17, and SF-25 (right panel). Scale bars: 1mm (whole mount, left panels) and 50 µm (fluorescent micrograph panels) in **c**. Dotted boxes in **c** and **f** denote regions of interest. Scale bars in **c** and **f**: 1mm (whole mount, left panels) and 50 µm (micrograph panels).

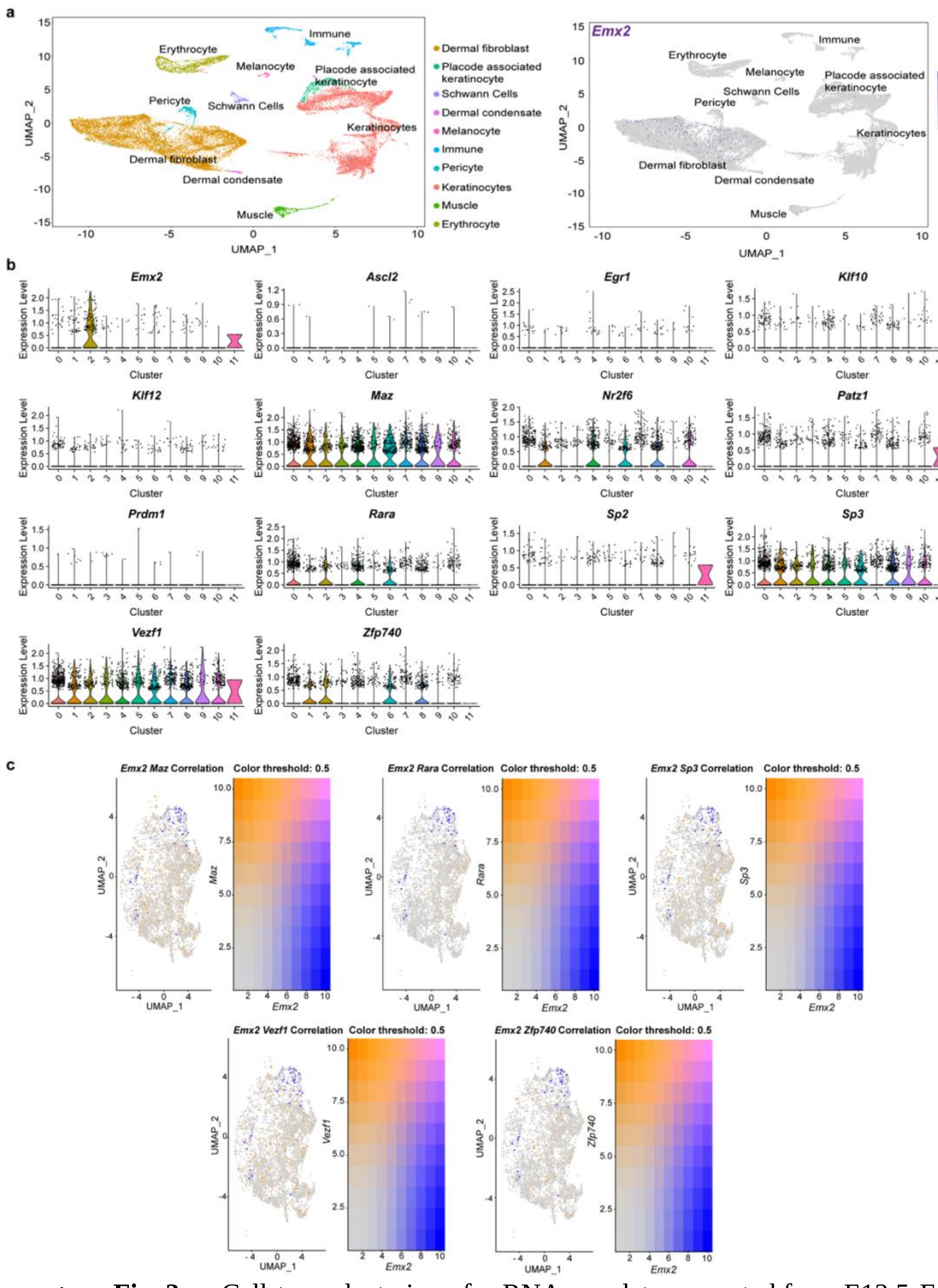

Supplementary Fig. 2. a, Cell-type clustering of scRNA-seq data generated from E12.5-E15.5 laboratory mouse trunk skin (left panel). Expression of *Emx2* is restricted to dermal fibroblasts (right panel). **b**, Violin plots of the 11 dermal fibroblast subclusters displaying expression levels of *Emx2* and of the different genes predicted to bind to motifs enriched in glider accelerated regions (i.e., GARs) and upregulated in the sugar glider patagium. **c,** Visualization of co-expression between *Emx2* and the five genes expressed in the same fibroblast subcluster as *Emx2* (i.e., subcluster 2).

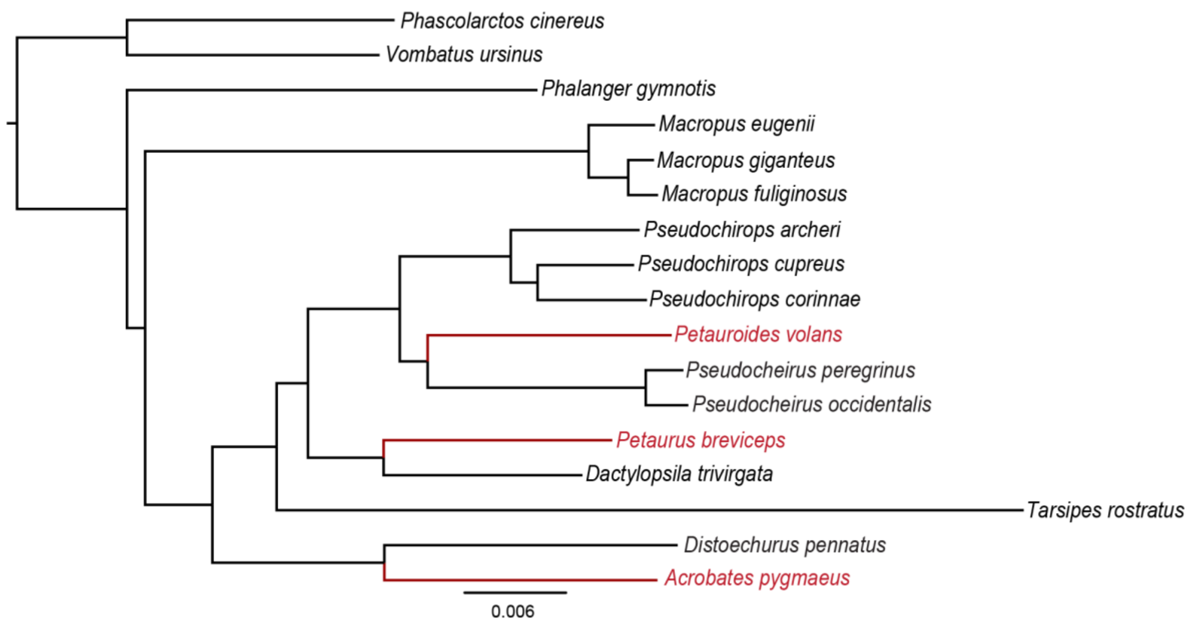

Supplementary Fig. 3. Tree topology. Species tree topology estimated from 1st and 2nd codon positions. All displayed branches have 100% bootstrap support. Topology is consistent with the independent evolution of patagia in three petauroid species (labeled in red font): *Petaurus breviceps*, *Petauroides volans*, and *Acrobates pygmaeus.* There is a complete match between this topology and the one generated with neutral sites shown in Fig. 1e.

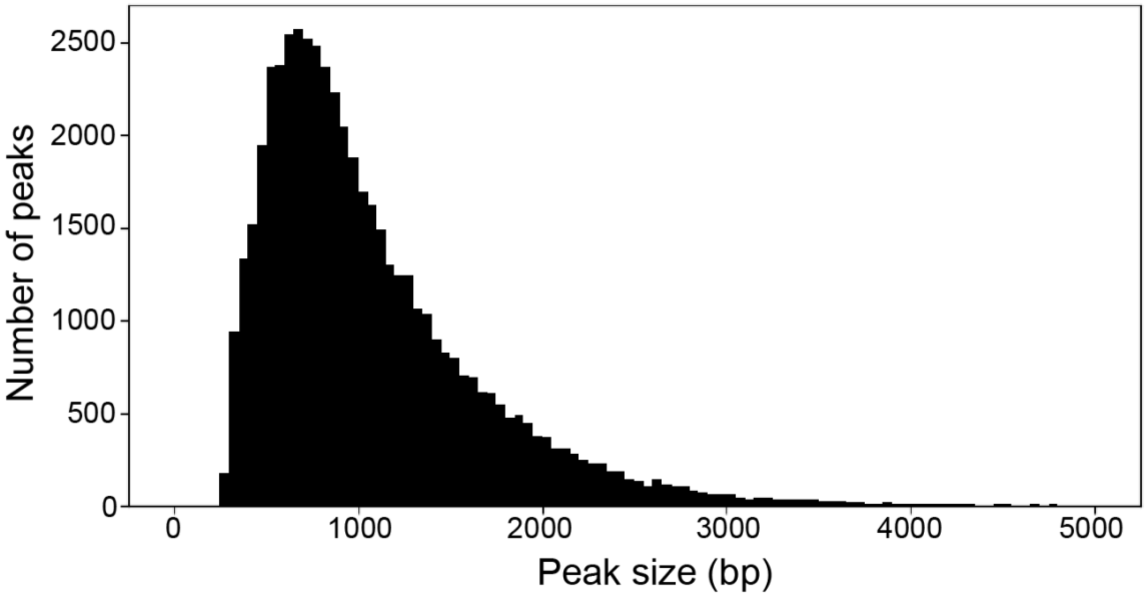

Supplementary Fig. 4. Size distribution of the 52,169 sugar glider candidate cis-regulatory elements.

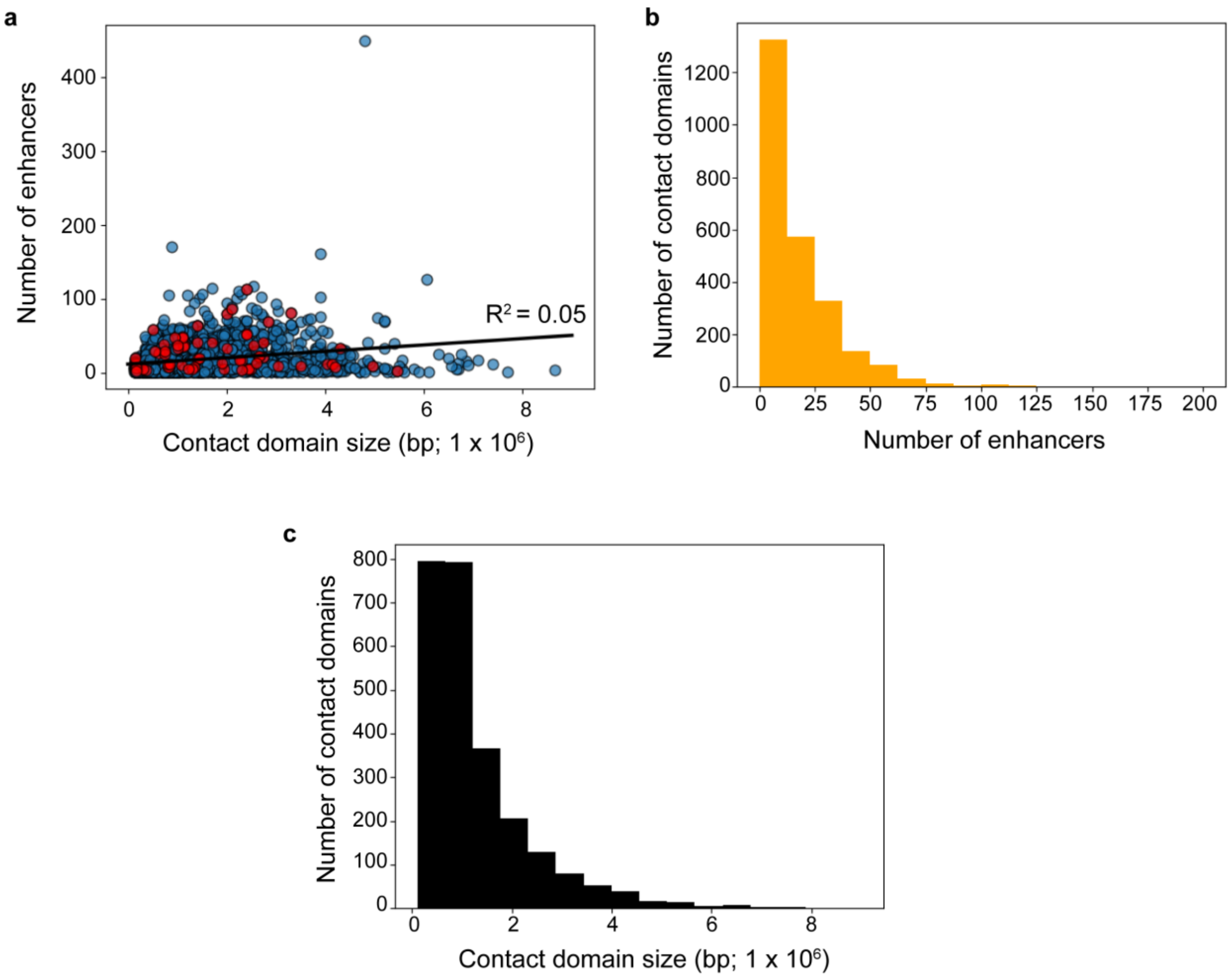

Supplementary Fig. 5. Relationship between contact domain size and enhancer number. a, Correlation between contact domain size and number of enhancers. **b**, Contact domain size distribution. **c**, enhancer distribution in the different contact domains. Red dots in **a** correspond to contact domains containing the 59 genes upregulated in the patagium primordium of wildtype sugar gliders and downregulated in patagia injected with shRNAEmx2_3.

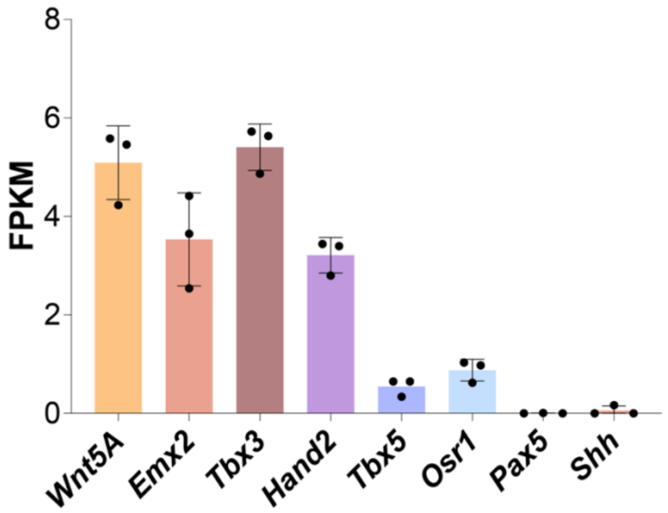

Supplementary Fig. 6. RNA-seq profiling of immortalized sugar glider dermal fibroblasts. Shown are FPKM values (Mean + SE; N= 3) for selected genes that we previously found to be expressed (i.e., *Emx2*, *Tbx3*, *Hand2*, *Tbx5*, *Osr1*, and *Wnt5a*) and not expressed (i.e., *Pax5* and *Shh*) in the sugar glider patagium.

Supplementary Tables

Supplementary Table 1. Genome metrics for the species for which we generated genome assemblies. We refer to N50 as the largest length such that 50% of all nucleotides in the genome assembly are contained in scaffolds/contigs of at least that size. L50 is defined as the smallest number of scaffolds/contigs which make up half of the genome assembly.

Species	Scaffold length (Mb)	Contig length (Mb)	Gap %	Scaffold L50	Scaffold N50 (Mb)	Contig L50	Contig N50 (bp)
Acrobates pygmaeus	3921.75	3892.51	0.745	22650	0.01	92609	10130
Distoechurus pennatus	3611.11	3575.54	0.985	4	356.66	48009	20090
Dactylopsila trivirgata	4108.35	3611.72	12.088	5	317.87	134907	6241
Macropus fuliginosus	3638.57	3609.87	0.789	5	341.28	29935	30302
Macropus giganteus	3535.64	3508.38	0.771	4	392.87	22824	41690
Pseudochirops archeri	3478.08	3448.48	0.851	8	157.99	42583	22267
Petaurus breviceps	3408.64	3344.79	1.873	4	375.54	14460	68444
Pseudochirops corinnae	3503.58	3469.81	0.964	5	209.25	35899	26947
Pseudochirops cupreus	3579.98	3546.63	0.932	4	425.24	34610	27965
Phalanger gymnotis	3624.08	3590.53	0.926	4	353.05	34302	28064
Pseudocheirus occidentalis	3603.85	3569.49	0.953	7	213.46	39429	24462
Pseudocheirus peregrinus	3653.86	3621.62	0.882	6	225.19	32746	29458
Petauroides volans	3693.03	3659.70	0.902	10	147.17	61039	15860
Tarsipes rostratus	4511.50	2793.78	38.074	6	278.43	280206	1860
Vombatus ursinus	3341.35	3323.28	0.541	3	576.11	11795	74778

Supplementary Table 2. Public genomes accessed in the present study.

Species name	GenBank ID/Link to genome	Assembly Name
Macropus eugenii	https://www.dnazoo.org/assemblies/Macropus_eugenii	me-1k
Phascolarctos cinereus	GCA_002099425.1	phaCin_unsw_v4.1

Supplementary Table 3. Number of GARs found in the genes that were enriched for GARs and upregulated in the sugar glider patagium. *Emx2*, highlighted in yellow, is the only gene that had GARs in the three glider species. RNA-seq data was corrected for multiple comparisons; FDR < 0.1).

Gene Name	Peaks	pValue	GAR count	Species
Cd248	3	0.000726659	2	*A. pygmaeus*
Lrrn1	1	0	1	*P. volans*
Loc110195665	2	0.008084879	1	*P. volans*
Gem	2	0.008084879	1	*A. pygmaeus*
Loc110220503	5	0.006322383	2	*A. pygmaeus, P. volans*
Loc110222792	2	0.008084879	1	*A. pygmaeus*
Jam3	2	0.008084879	1	*P. breviceps*
Cadm2	2	0.008084879	1	*P. breviceps*
Fut8	1	0	1	*P. breviceps, P. volans*
Nov	4	0.002710756	2	*A. pygmaeus, P. breviceps*
Spocd1	2	0.008084879	1	*A. pygmaeus*
Hmcn1	5	0.006322383	2	*P. breviceps*
Tnk2	2	0.008084879	1	*P. volans*
Emx2	16	0.001906554	5	*A. pygmaeus (3), P. volans, P. breviceps*
Tcf4	6	0.000843899	3	*P. breviceps*
Pyroxd2	2	0.008084879	1	*P. volans*
Entpd1	2	0.008084879	1	*P. volans*
Tll2	1	0	1	*A. pygmaeus*
Nxph3	2	0.008084879	1	*P. breviceps*
Map1b	10	0.001003676	4	*A. pygmaeus, P. volans (3)*
Kirrel3	10	0.008798515	3	*A. pygmaeus (2), P. volans*
Mmp28	2	0.008084879	1	*P. breviceps*
Prrt2	2	0.008084879	1	*A. pygmaeus*

Supplementary Table 4. List of genes downregulated in patagia injected with shRNAEmx2_3 and upregulated in wildtype sugar glider patagia. Labeled with 'a' are genes in which EMX2-bound sites overlapped with ATAC/ChIP (H3k27ac) peaks. Labeled with 'b' are genes in which EMX2-bound sites overlapped with assigned GARs.

Gene Name		
Acsf2	*Gria2*[a]	*Plekhg1*[a,b]
Adamts15[a,b]	*Hmcn1*[a,b]	*Plekhh2*[a]
Atp2b4[a]	*Hoxb9*	*Pou3f3*
Barx2[a]	*Hspa12b*[a]	*Prokr1*[a]
Cacna2d3[a,b]	*Ick*[a]	*Ptchd1*[a]
Cadm1[a,b]	*Igdcc4*	*Ptk7*
Cdc42ep4[a,b]	*Igfbp5*[a]	*Ptprr*[a]
Cilp	*Lmx1b*[a]	*Pxdnl*[a]
Cunh14orf37	*Loc110201139*	*Sdc3*[a]
Cunh2orf40[a]	*Lrp1b*[a]	*Spred1*
Dixdc1[a]	*Lrrtm1*[a]	*Tshr*
Dok6[a,b]	*Ltbp4*	*Tspan9*[a]
Emx2[a,b]	*Mmp15*	*Ube2ql1*
Enpp2[a]	*Mpdz*[a]	*Vasn*
Entpd2[a]	*Nov*[a]	*Wnt5a*[a]
Epha4[a]	*Ntng1*	*Gap43*
Etv4[a]	*Parva*[a]	*Gfra2*
Fbln7	*Pax1*[a]	*Pi15*[a]
Fbn3[a]	*Pcdh10*	*Pirt*[a]
Gabrb2	*Pdgfra*	

Supplementary Table 5. List of shRNA sequences and qPCR primer sequences used in this study.

Experiment	Name	Sequence
shRNA	shRNAEmx2_1	CAGGCTAGTCCAGAGGAAATA
	shRNAEmx2_2	GGCCACAGATTTCAAGGAAAT
	shRNAEmx2_3	CCTTTGAGAAGAATCACTATG
	shRNAEmx2_4	CCTCACGGAAACTCAGGTAAA
	shRNAEmx2_5	ACAGAAGAACGAAGTTCAAAC
	shScram Control	CCTAAGGTTAAGTCGCCCTCG
qPCR	Emx2_F	GCCTCACGGAAACTCAGGTA
	Emx2_R	TCCTCTGGACTAGCCTGTTTG
	Bactin_F	GAGGGTACAGTTTCACCACCA
	Bactin_R	TTTTCCAGAGAGGAGCTGGA

Supplementary References

93. Chen, A. *et al.* Spatiotemporal transcriptomic atlas of mouse organogenesis using DNA nanoball-patterned arrays. *Cell* **185**, 1777-1792.e21 (2022).
94. Ren, H., Walker, B. L., Cang, Z. & Nie, Q. Identifying multicellular spatiotemporal organization of cells with SpaceFlow. *Nat. Commun.* **13**, 1–14 (2022).
95. Nishioka, K. *et al.* Trps1 deficiency enlarges the proliferative zone of growth plate cartilage by upregulation of Pthrp. *Bone* **43**, 64–71 (2008).
96. Chung, I.-H., Han, J., Iwata, J. & Chai, Y. Msx1 and Dlx5 function synergistically to regulate frontal bone development. *Genesis* **48**, 645–655 (2010).
97. Kawai, S., Yamauchi, M. & Amano, A. Zinc-finger transcription factor Odd-skipped related 1 regulates cranial bone formation. *J. Bone Miner. Metab.* **36**, 640–647 (2018).
98. Muzio, L., Soria, J. M., Pannese, M., Piccolo, S. & Mallamaci, A. A mutually stimulating loop involving emx2 and canonical wnt signalling specifically promotes expansion of occipital cortex and hippocampus. *Cereb. Cortex* **15**, 2021–2028 (2005).
