# Supplementary material for: Emx2 underlies the development and evolution of marsupial gliding membranes
Source: Nature. 2024 Apr 24;629(8010):127–35. doi: 10.1038/s41586-024-07305-3 (PMC11062917; doi:10.1038/s41586-024-07305-3)
Supplement: Supplementary file 2 — Reporting Summary [file 41586_2024_7305_MOESM2_ESM.pdf]

nature portfolio

Corresponding author(s): Ricardo Mallarino

Last updated by author(s): Feb 22, 2024

Reporting Summary

Nature Portfolio wishes to improve the reproducibility of the work that we publish. This form provides structure for consistency and transparency in reporting. For further information on Nature Portfolio policies, see our Editorial Policies and the Editorial Policy Checklist.

Statistics

For all statistical analyses, confirm that the following items are present in the figure legend, table legend, main text, or Methods section.

n/a	Confirmed	
☐	☒	The exact sample size (*n*) for each experimental group/condition, given as a discrete number and unit of measurement
☐	☒	A statement on whether measurements were taken from distinct samples or whether the same sample was measured repeatedly
☐	☒	The statistical test(s) used AND whether they are one- or two-sided<br>*Only common tests should be described solely by name; describe more complex techniques in the Methods section.*
☐	☒	A description of all covariates tested
☐	☒	A description of any assumptions or corrections, such as tests of normality and adjustment for multiple comparisons
☐	☒	A full description of the statistical parameters including central tendency (e.g. means) or other basic estimates (e.g. regression coefficient) AND variation (e.g. standard deviation) or associated estimates of uncertainty (e.g. confidence intervals)
☐	☒	For null hypothesis testing, the test statistic (e.g. *F*, *t*, *r*) with confidence intervals, effect sizes, degrees of freedom and *P* value noted<br>*Give P values as exact values whenever suitable.*
☒	☐	For Bayesian analysis, information on the choice of priors and Markov chain Monte Carlo settings
☒	☐	For hierarchical and complex designs, identification of the appropriate level for tests and full reporting of outcomes
☒	☐	Estimates of effect sizes (e.g. Cohen's *d*, Pearson's *r*), indicating how they were calculated

Our web collection on statistics for biologists contains articles on many of the points above.

Software and code

Policy information about availability of computer code

Data collection	NIS Elements v5 (Nikon) was used to acquire microscopy images.
Data analysis	megahit v1.1.4.2, redundans v0.14a, ragtag v2.0.1, NGmerge v0.2_dev, picard MarkDuplicates v2.21.4-SNAPSHOT, bowtie2 v2.4.2, IDR v2.0.4.2, bedtools v2.27.1, RAxML v8.2.12, R v3.5.3, rphast v1.6.9, mafft v7.453, trimAl v1.4.rev15, bwa v0.7.17-r1188, pairtools v0.3.0, juicertools v1.22.01, samtools v1.12, mustache v1.0.1, cooler v0.8.5, higlass-manage v0.8.0, clodius v0.3.5, DESeq2 v1.34.0, LiftOff v1.6.3, BUSCO v5.4.4, FIJI v2.1.0, gffread v0.12.7, trimmomatic v0.39, MEME v5.5.4, BLAT, STAR v2.7.9a, feattureCounts v2.0.1, MACS2 v2.2.7.1, Seurat package v4.3.0.<br><br>Code availability<br>Code used for all analyses is available in a FigShare repository: https://figshare.com/s/81cf39b7de363f1526a1

For manuscripts utilizing custom algorithms or software that are central to the research but not yet described in published literature, software must be made available to editors and reviewers. We strongly encourage code deposition in a community repository (e.g. GitHub). See the Nature Portfolio guidelines for submitting code & software for further information.

Data

Policy information about availability of data

All manuscripts must include a data availability statement. This statement should provide the following information, where applicable:

- Accession codes, unique identifiers, or web links for publicly available datasets
- A description of any restrictions on data availability
- For clinical datasets or third party data, please ensure that the statement adheres to our policy

Data availability: All genome assemblies reported, and corresponding genome sequencing reads are submitted under NCBI PRJNA512907. The ATAC-seq, ChIP-seq, Micro-C, and RNA-seq reads are submitted under NCBI BioProject: PRJNA1078418

Human research participants

Policy information about studies involving human research participants and Sex and Gender in Research.

Reporting on sex and gender	n/a
Population characteristics	n/a
Recruitment	n/a
Ethics oversight	n/a

Note that full information on the approval of the study protocol must also be provided in the manuscript.

Field-specific reporting

Please select the one below that is the best fit for your research. If you are not sure, read the appropriate sections before making your selection.

☒ Life sciences ☐ Behavioural & social sciences ☐ Ecological, evolutionary & environmental sciences

For a reference copy of the document with all sections, see nature.com/documents/nr-reporting-summary-flat.pdf

Life sciences study design

All studies must disclose on these points even when the disclosure is negative.

Sample size	No predetermined sample size calculation was performed in this study. Sample size was chosen based on the numbers accepted by the field to run the relevant statistical tests described. The sample sizes used are sufficient to provide the desired statistical power for our analyses
Data exclusions	No data were excluded for experiments involving micro-C, RNA-seq, ATAC-seq, immunohistochemistry, histology, in situ hybridizations, or lentiviral experiments. We carried out outlier analysis for luciferase assays, as luminescence data from plate readers can be noisy. For all our GARs, we used 6 technical replicates and, in a few cases (as noted in the figure legends), we excluded a maximum of one data point per treatment based on our statistical outlier analysis.
Replication	All experiments involving antibody stains and in situ hybridizations were carried out in three different individuals and they all yielded the same results. LacZ based transgenic experiments were replicated in at least two animals per construct tested and all yielded the same results. Each luciferase assay was carried out in duplicate and both experiments yielded the same result. All other experiments, including RNAseq, ATACseq, ChIPseq, and Micro-C were done with multiple biological replicates, as indicated in the figure legends and methods.
Randomization	Samples/organisms were allocated into groups based on genotype/experimental conditions
Blinding	All measurements were taken from images in which the researcher performing the measurements was unaware of which images corresponded to which genotypes/experimental conditions

Reporting for specific materials, systems and methods

We require information from authors about some types of materials, experimental systems and methods used in many studies. Here, indicate whether each material, system or method listed is relevant to your study. If you are not sure if a list item applies to your research, read the appropriate section before selecting a response.

Materials & experimental systems

n/a	Involved in the study	
☐	☒	Antibodies
☐	☒	Eukaryotic cell lines
☒	☐	Palaeontology and archaeology
☐	☒	Animals and other organisms
☒	☐	Clinical data
☒	☐	Dual use research of concern

Methods

n/a	Involved in the study	
☐	☒	ChIP-seq
☒	☐	Flow cytometry
☒	☐	MRI-based neuroimaging

Antibodies

Antibodies used

Primary antibodies: anti-H3K27ac antibody (Abcam ab4729: lot # GR232896-1; 4ug), Anti-EMX2 antibody (Novus NBP2-39052: lot # 27711; (dilution: 1:50), anti-IgG antibody (Millipore 12-370; lot # 297424; 4 ug). Anti-GFP (Novus Biologicals # NB100-1614, Dilution: 1:200). anti-KRT14 (BioLegend #905301, Dilution: 1:1000)
Secondary antibodies: Alea-Fluor 488 (ThermoFisher; ab150169; Dilution 1:500); Goat Anti-Rabbit Biotinylated (Vector Labs, R.T.U. (BP-9100-50; Ready-to-use dilution)

Validation

Primary antibody validation:
1) Anti-EMX2 antibody: The target protein was examined with an antibody independent strategy (in this case, RNA sequencing in human tissue) and compared with results from an antibody-dependent strategy. A correlation between these two strategies indicated specificity between the antibody and Emx2. Manufacturer: https://www.novusbio.com/products/emx2-antibody_nbp2-39052
2) anti-H3K27ac antibody (Confirmed species reactivity: Mouse, Rat, Cow, Human; Predicted: Chicken, Xenopus laevis, Arabidopsis thaliana, Drosophila melanogaster, Monkey, Zebrafish, Plasmodium falciparum, Rice, Cyanidioschyzon merolae. Applications:ICC/IF, WB, IHC-P, ChIP; Validation: Manufacturer https://www.abcam.com/products/primary-antibodies/histone-h3-acetyl-k27-antibody-chip-grade-ab4729.html)
3) anti-IgG antibody: Validated by IP/WB as a non-specific IgG control. Manufacturer: https://www.emdmillipore.com/US/en/product/Normal-Rabbit-IgG,MM_NF-12-370)
4) anti-GFP: Validated on transgenic mice expressing recombinant GFP; https://www.novusbio.com/products/gfp-antibody_nb100-1614#datasheet
5) anti-KRT14 (Confirmed Species reactivity: Human; Applications: Immunohistochemistry; Validation: Manufacturer -https://d1spbj2x7qk4bg.cloudfront.net/Files/Images/media_assets/pro_detail/datasheets/905301_V09.pdf?v=20230922064116)

Eukaryotic cell lines

Policy information about cell lines and Sex and Gender in Research

Cell line source(s)	Sugar glider dermal fibroblasts from a male joey (25 days of age)
Authentication	Sugar glider dermal fibroblasts were authenticated by RNA sequencing
Mycoplasma contamination	All cell lines tested negative for Mycoplasma
Commonly misidentified lines (See ICLAC register)	No commonly misidentified cell lines were used in this study

Animals and other research organisms

Policy information about studies involving animals; ARRIVE guidelines recommended for reporting animal research, and Sex and Gender in Research

Laboratory animals	Mus Musculus (laboratory mouse) from the following genotypes: (1) RosaEmx2-GFP, (2) Emx2-Cre, (3) Pdgfra-CreERT2, (4) FVB/N769Tg(tetO-Wnt5a)17Rva/J, (5) B6.Cg Gt(ROSA)26Sortm1(rtTA*M2)Jae/J. The age of all mice ranged from Embryonic Day 11.5 to Postnatal day 40. Petaurus breviceps (sugar glider) of ages ranging from Postnatal day 2-14
Wild animals	Study did not involve wild animals
Reporting on sex	Sex was not considered in study design as the patagium growth is indistinguishable between sexes.
Field-collected samples	Study did not include field-collected samples
Ethics oversight	Princeton University's Institutional Animal Care and Use Committee approved all experiments and protocols.

Note that full information on the approval of the study protocol must also be provided in the manuscript.

ChIP-seq

Data deposition

☒ Confirm that both raw and final processed data have been deposited in a public database such as GEO.

☒ Confirm that you have deposited or provided access to graph files (e.g. BED files) for the called peaks.

Data access links<br>*May remain private before publication.*	ChIP-seq reads are submitted under NCBI BioProject: PRJNA1078418
Files in database submission	Emx2_Pbrev_ChIP_2456432000_peaks.narrowPeak, Control_Pbrev_ChIP_2456432000_peaks.narrowPeak,<br>zr2392_2_1_NGm_TM_X1k_vs_HiC.sorted.rmDup.rmMulti.filtered_nolambda_g2456432000_peaks.broadPeak,<br>zr2392_3_1_NGm_TM_X1k_vs_HiC.sorted.rmDup.rmMulti.filtered_nolambda_g2456432000_peaks.broadPeak,<br>zr2392_2_3_NGm_TM_X1k_vs_HiC.sorted.rmDup.rmMulti.filtered_nolambda_g2456432000_concatenated_merged_peaks<br>Numbered.broadPeak
Genome browser session<br>(e.g. UCSC)	No longer applicable

Methodology

Replicates	For H3K27Ac ChIP-seq, chromatin was divided into 2 experimental (each experimental sample consisting of pooled tissue from 6-7 joeys) and 1 control sample and ChIP assays were performed in triplicate for each of the experimental samples and in duplicate for the negative control sample. In all cases, we used 7 µg of chromatin and 4 µg of antibody. ChIP DNA was then processed into 3 standard Illumina ChIP-seq libraries (2 experimental and 1 control) and sequenced to generate ~25 million reads per sample.<br><br>EMX2 ChIP-Seq was carried out in a similar way as described above, except that only one experimental (pooled tissue from 6-7 joeys) and one control library were generated and sequenced.
Sequencing depth	H3K27ac: paired-end; control 56,614,872 97.3% mapped; experimental 1: 56,957,310 97.4% mapped; experimental 2: 70,392,940 97.4% mapped<br><br>Emx2: single-end; experimental: 41,672,698 ~100% mapped ; control: 43,909,990 ~100% mapped
Antibodies	anti-H3K27ac antibody (Abcam ab4729: lot # GR232896-1), Anti-EMX2 antibody (Novus NBP2-39052: lot # 27711), anti-IgG antibody (Millipore 12-370; lot # 297424).
Peak calling parameters	H3K27ac: --broad -f BAMPE -g 2456432000 -q 0.05 --nolambda<br>Emx2: -f BAMPE -g 2456432000
Data quality	H3k27ac: The input was used as a background control. Peaks between the two replicates were concatenated and overlapping peaks were merged using BEDTools merge.<br>Emx2: Input was used as a background control. FIMO was used to scan for enriched motif and the canonical Emx2 binding motif was recovered.
Software	NGmerge v0.2_dev, picard MarkDuplicates v2.21.4-SNAPSHOT, bowtie2 v2.4.2, bedtools v2.27.1
