# Supplementary material for: Emx2 underlies the development and evolution of marsupial gliding membranes
Source: Nature. 2024 Apr 24;629(8010):127–35. doi: 10.1038/s41586-024-07305-3 (PMC11062917; doi:10.1038/s41586-024-07305-3)
Supplement: Supplementary file 8 — Alignment of GARs across marsupial species [file 41586_2024_7305_MOESM8_ESM.pdf]

GAR 11730

	10 20 30 40	
Petaurus breviceps	CTCAAACACATTCCAACCAACACAACCATCCGAGTTGGAG	40
Acrobates pygmaeus	CTCAAACACATTCCAACCAACACAACCATCCGAGTTGGAG	40
Distoechurus pennatus	CTCAAACACATTCCAACTAACACAAGTATCCGAGTTGGAG	40
Dactylopsila trivirgata	CCCAAACACATTCCAACCAACACAACCATCCGAGTTGGAG	40
Macropus giganteus	CTCAAACACATTCCAACCAACACAACCATCCGAGTTAGAG	40
Pseudochirops archeri	CTCAAACACATTCCAACCAATACAACCATCCGAGGTGGAA	40
Phascolarctos cinereus	CTCAAACACATTCCAACCAACACAACCATTCGAGTTGGAG	40
Pseudochirops corinnae	CTCAAACACATTCCAACCAATACAACCATCCGAGGTGGAA	40
Pseudochirops cupreus	CTCAAACACATTCCAACCAATACAACCATCCGAGGTGGAA	40
Phalanger gymnotis	-----ACACATTCCAATCAACACAACCAACAGAACTGGAG	35
Pseudocheirus occidentalis	CCCAAACACATTCCAACCAACACAACCATCCGAGTTGGAG	40
Pseudocheirus peregrinus	CCCAAACACATTCCAACCAACACAACCATCCGAGTTGGAG	40
Petauroides volans	CTCAAACACATTCCAACCAACACAACCATCCGAGTTGGAG	40
Tarsipes rostratus	CTCAAACACATTCCAACTAGCAAAACCGTCCGACCTGGAG	40
Vombatus ursinus	CTCAAACACATTCCAACCAACACAACCATTCGAGTTGGAG	40

	50 60 70 80	
Petaurus breviceps	GCTTCTTCGCCTAGGTCCCCCATCCCGGGGAGCTGGGCCC	80
Acrobates pygmaeus	GTTTCTTCTCCTATGTCCTCCATCCCAGGGAGCTGGGCCC	80
Distoechurus pennatus	GTTTCTTTTCCTAGGTTCCCCATCCCAGAGAGTTGGGCCC	80
Dactylopsila trivirgata	GCTTCTTCGCCTCGGTCCCCTATCCCAGGGAGCTGGGCCC	80
Macropus giganteus	GTTTCTTCGTCTAGGTCCCCCATCCCAGGGAGATGGGCCC	80
Pseudochirops archeri	GTTTCTTCGCCTAGGTCCCCCATCCCACGGAGCTGGGCCC	80
Phascolarctos cinereus	GTTTCTTCGTCTAGGTCCCCCGTCCCAGGGAGCTGGGCCC	80
Pseudochirops corinnae	GTTTCTTCGCCTAGGTCCCCCATCCCACCGAGCTGGGCCC	80
Pseudochirops cupreus	GTTTCTTCGCCTAGGTCCCCCATCCCACGGAGCTGGGCCC	80
Phalanger gymnotis	GTTCCTTCGCCTAGGTTACCCATCCCAGGGGCCTGCGCCC	75
Pseudocheirus occidentalis	GTTTCTTCGCCTAGGTCCCCCATCCCACGGAGCTGGGCCC	80
Pseudocheirus peregrinus	GTTTCTTCGCCTAGGTCCCCCATCCCACGGAGCTGGGCCC	80
Petauroides volans	GTTTCTTCGCCTAGGTCTCCCATCCCACGGAGCTGAGCCC	80
Tarsipes rostratus	GCTTCTTCGCCTAGGTCCCCCATCC-------CTGGGCCC	73
Vombatus ursinus	GTTTTTTCGTCTAGGT-CCCCGTCCCAGGGAGCTGGGCCC	79

	90 100 110 120	
Petaurus breviceps	ATTTCCTCAGTTGGCTCAGGCCCTCAAGGAAACTACCAGG	120
Acrobates pygmaeus	AGTTTCTCAGTTAGCTCAGGCCCTCAGGGAAACTACCAGG	120
Distoechurus pennatus	AGTTTCTCAGTTAGCTCAGGCCCTCAAGGAAACTACCAGG	120
Dactylopsila trivirgata	ATTTCCTCAGTTAGCTCAGGCCCTGAAGGAAACTACCAGG	120
Macropus giganteus	AGGTT-TCTGTTAGCTCAGGCCCTCAAGGAAATCACCAGG	119
Pseudochirops archeri	AGTTCCTCAGCTAGCTCAGGCCCTCAAGGAAACTACCAGG	120
Phascolarctos cinereus	AGTTTCTCAGTTAGCTCAGGCCCTCAAGGAAACCACCAGG	120
Pseudochirops corinnae	CGTTCCTCAGTTAGTTCAGGCCCTCAAGGAAACTACCAGG	120
Pseudochirops cupreus	AGTTCCTCAGTTAGCTCAGGCCCTCAAGGAAACTACCAGG	120
Phalanger gymnotis	AGTTTCTTAGTTAGCTCAGGCCCTCAAGGAAACCACCAGG	115
Pseudocheirus occidentalis	AGTTCCTCAGTTAGCTCAGGCCCTCAAGGAAACTACCAGG	120
Pseudocheirus peregrinus	AGTTCCTCAGTTAGCTCAGGCCCTCAAGGAAACTACCAGG	120
Petauroides volans	AGTTCCTCAGTTAGCTCAGGCCCTCAAGGAAACTACCAGG	120
Tarsipes rostratus	GGTTCATCTGTTAGCTCGGGCCCTCAAGGAAACTATCAGG	113
Vombatus ursinus	AGTTTCTCAGTTAGCTCAGGCCCTCAAGTAAACCACCAGG	119

Species	130 140 150 160	
Petaurus breviceps	TGCAAACTCCCTTGGGGGAGGGGCAGCCCTGGGTAAAGGG	160
Acrobates pygmaeus	TGCAAACTCCCTTGGGGGAGGGGCAGCCGTGGGCAAAGGG	160
Distoechurus pennatus	TGCAAACTCCCTTGGGGGAGGGGCAGCCCTGGGCAAAGGG	160
Dactylopsila trivirgata	TGCAAACTCCCTTGGGGGAGGGGCAGCGCTGGGTAAAGGG	160
Macropus giganteus	TGCAAACTCCCTTGGGGGAGGGGCAG-CCTGGGCAAAGGG	158
Pseudochirops archeri	TGCAAACTCCGTTGGGGGAGGGGCAGCCCTGCGCAAAGGG	160
Phascolarctos cinereus	TGCAAACTCCCTTGGGGGAGGGGCATCCCTGGGCAAAGGG	160
Pseudochirops corinnae	TGCAAACTCCGTTGGGGGAGGGGCAGCCCTGGGCAAAGGG	160
Pseudochirops cupreus	TGCAAACTCCGTTGGGGGAGGGGCAGCCCTGGGCAAAGGG	160
Phalanger gymnotis	TGCAAACTCCCTTGGGGGAGGGGCAGCCCTGGGCAAAGGG	155
Pseudocheirus occidentalis	TGCAAACTCCTTTGGGGGAGGGGCAGCCCTGGGCAAAGGG	160
Pseudocheirus peregrinus	TGCAAACTCCTTTGGGGGAGGGGCAGCCCTGGGCAAAGGG	160
Petauroides volans	TGCAAACTCCATTGGGGGAGGGGCAGCCCTGGGAAAAAGG	160
Tarsipes rostratus	TGCAAACTCCCTTGGGGGAGGGGTAGCCCCGGGCAAGGGG	153
Vombatus ursinus	TGCAAACTCCCTTGGGGGAGGGGCATCCCTGGACAAAGGG	159

Species	170 180 190 200	
Petaurus breviceps	TGAAACCCAGGACGCGACCCCTGCTCCGCTGGCTCAGTGC	200
Acrobates pygmaeus	TGAAACCCAGGACACGACCCCTGCTCCGCTGGCTCGGTGC	200
Distoechurus pennatus	TGAAACCTGGGACACGACCCCTGCTCCGCTGGCTCGGTTC	200
Dactylopsila trivirgata	TGAAACCCAGGACATGACCCCTGCTCCGCTGGCTCAGTGC	200
Macropus giganteus	TGAAACCCAGGACACGACCCCTGCTCTGCTCGCTTTGTGC	198
Pseudochirops archeri	TGAAACCCAGGACACGACCCCTGCTCCGTTGGCTCAGTGC	200
Phascolarctos cinereus	TGAAACCTAGGACACGACCCCAGCTTGGCTCGCTCCGTGC	200
Pseudochirops corinnae	TGAAACCCAGGACACGACCCCTGCTCCGTTGGCTCAGTGC	200
Pseudochirops cupreus	TGAAACCCAGGACACGACCCCTGCTCCGTTGGCTCAGTGC	200
Phalanger gymnotis	TGAAACCCAGGACACGACCCCTGCTCTGCTCGCTGTGTGC	195
Pseudocheirus occidentalis	TGAAACCCAGGACATGACCCCTGCTCCGCTAGCTCGGTGC	200
Pseudocheirus peregrinus	TGAAACCCAGGACATGACCCCTGCTCCGCTAGCTCGGTGC	200
Petauroides volans	TGAAACCCAGGACACGACCCCTGCTCCATTGGCTCCGTGC	200
Tarsipes rostratus	TGAAACCCAGGACACGACCCCTGCTCCGCCGGCTCAAAGC	193
Vombatus ursinus	TGAAACCCAGGACACGACCCCTGCTCCGCTCGCTCCGTGC	199

Species	210 220 230 240	
Petaurus breviceps	TACGAATGAAACCTGGAAAGTGCGGGAGGGAGGGG-----	235
Acrobates pygmaeus	TACGAACGAAACCCGTAAGGTGCGGGAGGGAGTGGGGTTT	240
Distoechurus pennatus	TACGAAGGAAACCTGGAAAGTGAGGGAGGG-GGTGGGTTT	239
Dactylopsila trivirgata	TACGAATGAAACCTGGAAAGTTCGGGAGGGAGAGGGGGTT	240
Macropus giganteus	TACGAATGAAACCCGGAAAGTGAGGGAGGGAGGGGGGTTT	238
Pseudochirops archeri	TGCGAATGAAACCTGGAAAGTGCGGAAGGGAGAGGGGTTT	240
Phascolarctos cinereus	TACGAATGAAACCAGGAAAGTGCAGGAGGGAGGGGGATTT	240
Pseudochirops corinnae	TACGAATGAAACCTGGAAAGTGCGGAAGGGAGAGTGGTTT	240
Pseudochirops cupreus	TACGAATGAAACCTGGAAAGTGCGGAAGGGAGACGGGTTT	240
Phalanger gymnotis	TACGAATGAAACCCGGAAAGTGCGGGAGGGAGAGGGGTTT	235
Pseudocheirus occidentalis	TACGAATGAAACCTGGAAAGTGCGGGAGGGAGAGGGGTTT	240
Pseudocheirus peregrinus	TACGAATGAAACCTGGAAAGTGCGGGAGGGAGAGGGGTTT	240
Petauroides volans	TACGAATGAAACCTAGAAAGTGCAGGAGGGAGAGGCGTTT	240
Tarsipes rostratus	TAGGAATGAAACCTGGAAAGTGCGGGAGAGAGAGGGCTTT	233
Vombatus ursinus	TACGAATGAAACCCAGAAAGTGCAGGAGGAAGGGGGGGTT	239

	250 260 270 280	
Petaurus breviceps	---------------------------GTGGTTATACAGC	248
Acrobates pygmaeus	ACCCAAAAGAGACTACAGAATCTTCAGGCAACCCTCCCCC	280
Distoechurus pennatus	ACCCAAAAGAGACTACAGAATCTTCAGGCAACCACTTTTC	279
Dactylopsila trivirgata	ACCCAAAAGAGACTACAGAATCTTCTGGCAGCTGCTTTTT	280
Macropus giganteus	ACCCAAAAGTGACTACAGAATCTCTAAGCAACTGCTTTTT	278
Pseudochirops archeri	ACCCAGAAGAGACTACAAAATCTTCAGGCAACTGCTTTTT	280
Phascolarctos cinereus	ACCCCAAAGAGACTACAGATTCTCCAGGCAACTGCTTTTT	280
Pseudochirops corinnae	ACCCAGAAGAGACTACAAAATCTTCAGGCAACTGCTTTTT	280
Pseudochirops cupreus	ACCCAGAAGAGACTACAAAATCTTCAGGCAACTGCTTTTT	280
Phalanger gymnotis	ACCCAAAAGAGACTACAGAGTCTTCAGGCACCAGCTTTTT	275
Pseudocheirus occidentalis	ACCCAGAAGAGGCTACAGAATCTTCACGCAACTGCTTTTT	280
Pseudocheirus peregrinus	ACCCAGAAGAGGCTACAGAATCTTCACGCAACTCCTTTTT	280
Petauroides volans	ACCCAGAAGAGGCTACACAATCTTCAGGCAACTGCTTTTT	280
Tarsipes rostratus	ACCCAAAAGAGACTACACAACCTTCAGGCAACTGCTTTTT	273
Vombatus ursinus	ACCCAAAAGAGACTACAGAATCTTCAGGCAACTGCTTTTT	279

	290 300 310 320	
Petaurus breviceps	ACCTAGTAGCGTGGGCATGTACATATGTATGTATGAGTTA	288
Acrobates pygmaeus	CCTCCATATCATGGATATGCACATATTCGTGTATAAGTTA	320
Distoechurus pennatus	CCCTCCTATCGTGGATATGTACATATTAGTGTATAAGTTA	319
Dactylopsila trivirgata	ACCTACTAGCGTGGGTATGTACATATGTATGTATGAGTTA	320
Macropus giganteus	ATCTACTAGTGTGGGTATGTACATATGTGTGTATGAGTTA	318
Pseudochirops archeri	ACGTACTAGCGTGGGAATGTACATATGTGTGCATGAGTTA	320
Phascolarctos cinereus	ACCTACTAGCGTGGGTATGTACAAATGTGTGTATGAGTTA	320
Pseudochirops corinnae	ACGTACTAGCGTGGGAATGTACATATGTGTGCATGAGTTA	320
Pseudochirops cupreus	ACGTACTAGCGTGGGAATGCACATATGTGTGCATGAGTTA	320
Phalanger gymnotis	ATCTACTAGCGTGGGTATGTACATATGTGTGTATGAGCTA	315
Pseudocheirus occidentalis	ACGTACTAGCGCGGGTATGTACATATGTGTGTATGAGTTA	320
Pseudocheirus peregrinus	ACGTACTAGCGCGGGTATGTACATATGTGTGTATGAGTTA	320
Petauroides volans	ATGTACTAGC--------------------------GTTA	294
Tarsipes rostratus	ACCTACTAGAGTGGGTATACACATATGTGTATATGAGT--	311
Vombatus ursinus	ACCTACTAGTGTGGGTATGTACATATGTGTGTATGAGTTA	319

	330 340 350 360	
Petaurus breviceps	TGTATAATATGAGTATTTGCAAGTCCGTGCTGGGTACAGA	328
Acrobates pygmaeus	TGTACAATATGTGTATTTGCAAGTCAGTGCTGGGTAAAGA	360
Distoechurus pennatus	TGTACAATATGTGTATTTGCAAGTCAGTGCTGGGTAAAGA	359
Dactylopsila trivirgata	TGTACAATATGTGTATTTGCAAGTCAGTGCTGGGTAAAGA	360
Macropus giganteus	TGTACAATATGTGTATTTGCAAGTCAGTGCTGGGTAAAGG	358
Pseudochirops archeri	TGTACAATATGTGTATTTGCAAGTCAGTGCTGGGTAAAGA	360
Phascolarctos cinereus	TGTACAATAGGTGTATTTGCAAATCAGTGCTAGATAAAGA	360
Pseudochirops corinnae	TATACAATATATGTATTTGCAAGTCAGTGCTGGGTAAAGA	360
Pseudochirops cupreus	TGTACAATATGTGTATTTGCAAGTCAGTGCTGGGTAAAGA	360
Phalanger gymnotis	TGTACAATATTTGTATTTGCAA--CAGCGCTGGATAAAGA	353
Pseudocheirus occidentalis	TGTACAATATGTGTATTTGCAAGTTAGTGCTGGGTAAAGG	360
Pseudocheirus peregrinus	TGTACAATATGTGTATTTGCAAGTTAGTGCTGGGTAAAGG	360
Petauroides volans	TGTACAATATGTGTATTTGCAAGTCAGTGGTGGGTAAAGA	334
Tarsipes rostratus	------ATACGTGCATTTGCAAGTCAGTGCTGGGTAAAGA	345
Vombatus ursinus	TGTACAATATGTGTATTTGCAAATCAGTGCTGGATAAAGA	359

```
                              370       380       390       400
Petaurus breviceps            AATCAAGTTAGGGGTGGGGTATGCAAGAACTTTTTAACGC  368
Acrobates pygmaeus            AATCAAGTTAGGGATGGGGTATGCAAGAACTTTTTAGCAC  400
Distoechurus pennatus         --------TAGGGATGGGGTATGCACGAACTTTTTAGCAC  391
Dactylopsila trivirgata       AATCAAGTTAGGGGTGGGGTATGCAAGAACTTTTTAACAG  400
Macropus giganteus            AATCAAGTTAGGGGTGGGGTGTGCAAGAACTTTTTAACAA  398
Pseudochirops archeri         AACCAAGTTAGAGGTGGGATATGCAAGAACTTTTTAACAG  400
Phascolarctos cinereus        AATCAAGTTAGGGGTGGGGTATGCAAGAACTTTTTAGCAC  400
Pseudochirops corinnae        AACCAAGTTAGAGGTGGGGTATGCAAGAACTTTTTAACAG  400
Pseudochirops cupreus         AACCAAGTTAGAGGTGGGGTACGCAAGAACTTTTTAACAG  400
Phalanger gymnotis            AATCAAGTTAGACATGGGGTATGCAAGAACTTTTTAGCAG  393
Pseudocheirus occidentalis    AACCAAGTTAGGGGTAGGGTGTGCAAGAACTTTTTAGCAC  400
Pseudocheirus peregrinus      AACCAAGTTAGGGGTAGGGTGTGCAAGAACTTTTTAGCAC  400
Petauroides volans            AACCAAGTTAGGGGTGGGGTGTGCAAGAACTTTTTAGCAC  374
Tarsipes rostratus            AATCGAGTTAGGAGTGGGGTATGCAAGAACTTTTTAGCAC  385
Vombatus ursinus              AATCAAGTTAGGGGTGGGGTATGCAAGAACTTTTTAGCAC  399
```

```
                              410       420       430       440
Petaurus breviceps            G-TACTCCAGTCACTAATGCGCTGGGACACCTAAGAGAA-  406
Acrobates pygmaeus            GAAGTTCCAGCCACCAATGCTCTGGGATACCTAAGAGAAG  440
Distoechurus pennatus         GATGCTCTGTCCACTAATGCTCTAGGATACCTAATAGAAG  431
Dactylopsila trivirgata       GATACTCCAGTCACTAATGCGCTGGGATACCTAAGAGAAG  440
Macropus giganteus            GATATTACAGTCGCTAATGGGCTGGGATACCTAAGAGAAG  438
Pseudochirops archeri         GATACTCTAGTCACTAATGCGCTGGGATACCTAAAAGAAG  440
Phascolarctos cinereus        AATACTCCAGTCATTAATGCGCTAGGATACCTAAGAGAAG  440
Pseudochirops corinnae        GATACTCCAGTCACTAATGCGCTGGGATACCTAAAAGAAG  440
Pseudochirops cupreus         GATACTCCAGTCACTAATGCGCTGGGATACCTAAAAGAAG  440
Phalanger gymnotis            GATATTCTAGTCGCTA----------ATACCTAAGAGAAG  423
Pseudocheirus occidentalis    GAGACTCCAGTTACTAATGCGCTGGGATACCTAAGAGAAG  440
Pseudocheirus peregrinus      GAGACTCCAGTTACTAATGCGCTGGGATACCTAAGAGAAG  440
Petauroides volans            GAGACTCCAGTCACTAACGCGCTGGGATACCTAAAAGAAG  414
Tarsipes rostratus            GATATTCCAGTCACTAAGATGCTGGGCTACTTAACAAAAG  425
Vombatus ursinus              AAAACTCCAGTCATTAATGCGCTAGGATACCTAAGAGAAG  439
```

```
                              450       460       470       480
Petaurus breviceps            --GGTCCCTCAGTGTATGGTCCAACTTTCAGAAGGGATTT  444
Acrobates pygmaeus            AAGGTTCCTAAGTGTATGGTTCAATTTTCAGAAGGGATTT  480
Distoechurus pennatus         AAGGTTCCTAAGTGTATGGTTCAATTTTCAGAAGGAATTT  471
Dactylopsila trivirgata       ACGGTTCCTAAATGTATGGTCCAATTTTCAGAAGGGATTT  480
Macropus giganteus            ACGGTTCCTAAATGTATGGTCCAATTTTCAGAAGGGATTT  478
Pseudochirops archeri         ACGGTCCCTCAATGTATGGTCCAATTTTCAGAAGGGATTT  480
Phascolarctos cinereus        ACGGTTCCTAAATGTATGGTCCAATTTTCAGAAGGGATTT  480
Pseudochirops corinnae        ACGGTCCCTCAATGTATGGTCCAATTTTCAGAAGGGATTT  480
Pseudochirops cupreus         ACGGTCCCTCAATGTATGGTCCAATTTTCAGAAGGGATTT  480
Phalanger gymnotis            GCAGTTCCTAAATGTATAGTCCAGTTTTCAGAAGGGATTT  463
Pseudocheirus occidentalis    ACGGTCCCTAAATGTATGGTCCAATTTTCAGAAGGGATTT  480
Pseudocheirus peregrinus      ACGGTCCCTAAATGTATGGTCCAATTTTCAGAAGGGATTT  480
Petauroides volans            ACGGTCCCTAAATGTATCGTCCAGTTTTCAGAAGGGATTT  454
Tarsipes rostratus            ACAGGTCCT--ATATATGGTCCAATTTTCAGAAAGGATTT  463
Vombatus ursinus              ACGGTTCCTAAATGTATGGTCCAATTTTCAGAAGGGATTT  479
```

Species	490 500 510 520	
Petaurus breviceps	CTGAAGACAACACCCCCCATGCCAACACTAACAAAAATAA	484
Acrobates pygmaeus	CCAGAGACAACACCCTCCATGCCAACACTAACAAAAATAA	520
Distoechurus pennatus	CCAAAAACAACACCCGCCATGCCAATACTAACAAAAATAA	511
Dactylopsila trivirgata	CCGAAGACTACACCCCCCATGCCAACACTAACAAAAATAA	520
Macropus giganteus	TCAAAGACAACTCCCCCAATACCAACACTAACAAAAATAA	518
Pseudochirops archeri	CCAAACACAGCACCCCTCATGCCAACACAAACAAAAATAA	520
Phascolarctos cinereus	CCAAAGACAACACCCCCCATGCCAACACTAACAAAAATAA	520
Pseudochirops corinnae	CCAAACACAGCACCCCCCATGCCAACACAAACAAAAATAA	520
Pseudochirops cupreus	CCAAACACAGCACCCCCCATGCCAACACAAACAAAAATAA	520
Phalanger gymnotis	CCAAAGACA--ACCCCCCACGCCAACACTAACAAAAATAA	501
Pseudocheirus occidentalis	CCAAAGACAGCATCCCCCATGCCAACACAAACAAAAATAA	520
Pseudocheirus peregrinus	CCAAAGACAGCATCCCCCATGCCAACACAAACAAAAATAA	520
Petauroides volans	CCAAAGACAGCACCCCCCATGTCAACACAAACAAAAATAA	494
Tarsipes rostratus	CCAAAGACACCACCCCCCATGCCAACACTAACAAAAATAA	503
Vombatus ursinus	CCAAAGACAACACCCCCCATGCCAACACTAACAAAAATAA	519

Species	530 540 550 560	
Petaurus breviceps	GTTGATAAACAAGCAAATAAAGAAGGGGAAATGAGGGAGG	524
Acrobates pygmaeus	GTTGATAAACAAGCAAATAAAGAAGGGGAAATGGGGGAGG	560
Distoechurus pennatus	GTTGATAATCAAGTAAATAAAGAAGGGGAAATGTGGGGGG	551
Dactylopsila trivirgata	GTTGATAAACAAGCAAATAAAGAAGGGGAAATGGGAGAGG	560
Macropus giganteus	GTCTATAAACATGAAAATAAAGAAGGGGAAATGGGGGGGA	558
Pseudochirops archeri	GTTGATAAACAAGCAAATAAAGAAAGGGAAATGGGGGGGG	560
Phascolarctos cinereus	GTCAATAAACAAGCAAATAAAGAAGGGGAAATGGGGATAA	560
Pseudochirops corinnae	GTTGATAAACAAGCAAATAAAGAAGGGGAAATGGAGGGGA	560
Pseudochirops cupreus	GTTGATAAACAAGCAAATAAAGAAGGGGAAATGGGGGGGA	560
Phalanger gymnotis	TTCGATAAACAAGTAAATAAAGGGGAGGAAATGTGGGGGG	541
Pseudocheirus occidentalis	GTTGATAAACAAGCAAATAAAGGAGGGGAAATG-GGGGGG	559
Pseudocheirus peregrinus	GTTGATAAACAAGCAAATAAAGGAGGGGAAATGGGGGGGG	560
Petauroides volans	GTTGATAAACAAGCAAATAAAGAAGGGGAAATGGGTGGGG	534
Tarsipes rostratus	GTTGATAAACAAGCAAATAAAGAAGGGGAAATGGAAAGAG	543
Vombatus ursinus	GTCGATAAACAAGCAAATAAAGAAGGGGAAATGGGTATAA	559

Species	570 580 590 600	
Petaurus breviceps	AACGAGGTATGCGGTCACCCACTGGGCGAAAGAACGAGAT	564
Acrobates pygmaeus	AACGAGGTATGTAGTCACCCACTGGGCAAAAGAAAGA-TC	599
Distoechurus pennatus	GAAGAGGTATGCGGTCACCCATTGGGCAAAAGAAAGAGAT	591
Dactylopsila trivirgata	AACGAGGTATTCGGTCACCCACTGGGCGAAAGAACGAGAT	600
Macropus giganteus	GATGAGGTATGGGGTCACCTATTGGGCAAAAGAAAGAGCT	598
Pseudochirops archeri	GACGAGGTATGCGGTCACCCACTGGGCAAAAGAACTAGAT	600
Phascolarctos cinereus	GATGAGGTATGGGGTCACCCACAGGGCAAAAGAAAGAGAT	600
Pseudochirops corinnae	AACGAGGTATGCGGTCACCCACTGGGCAAAAGAACGAGAT	600
Pseudochirops cupreus	AACGAGGTATGCGGTCACCCACTGGGCAAAAGAACGAGAT	600
Phalanger gymnotis	GATGAGGGATGGGGTCACCCACTGGGAAAAAGAAATAGAT	581
Pseudocheirus occidentalis	GACGAGGTATGCGGTCACCCACTGTGCAAAAGAACGAGAT	599
Pseudocheirus peregrinus	GACGAGGTATGCGGTCACCCACTGTGCAAAAGAACGAGAT	600
Petauroides volans	GACGAGGTATGCGGTCACCCACTGGGCAAAAGAACGAGAT	574
Tarsipes rostratus	AACGAGGCATGCGGTCACCCACTGGGCAAAAGAACGAGAT	583
Vombatus ursinus	GAAGAGGTATGGGGTCACCCACTGAGCAAAAGAAAAAGAT	599

	610 620 630 640	
Petaurus breviceps	CCACAGATAATCACTTTCAGAAGAAATGTGTATCCAGACG	604
Acrobates pygmaeus	CTCAAGATAATCATTTTCAGAAGAAATGTGTATCCAGACG	639
Distoechurus pennatus	CTTCAGATAATCATTTTCAGAAGAAATGTGTATCCAGACA	631
Dactylopsila trivirgata	CCTCAGATAATCACTTTTTCAGAAGAAACATATCCAGACA	640
Macropus giganteus	CTTCAGATAATCACTTTCAGAGGAAATGTGTATCTAGACA	638
Pseudochirops archeri	CCTCAGATAATCACTTTCAGAAGAAATGTGTATCCAGACA	640
Phascolarctos cinereus	CCTCAGATAATCACTTTTAGAAGAAATGTGTATCCAGACA	640
Pseudochirops corinnae	CCTCAGATAATCACTTTCAGAAGAAATGTGTATCCAGACA	640
Pseudochirops cupreus	CCTCAGATAATCACTTTCAGAAGAAATGTGTATCCAGACA	640
Phalanger gymnotis	GCTCAGATAATCTCTTTCAGAAGAAATGTGTATCCAGACA	621
Pseudocheirus occidentalis	CCTCAGATAATCACTTTCAGAAGAAATGTGTATCCAGACA	639
Pseudocheirus peregrinus	CCTCAGATAATCACTTTCAGAAGAAATGTGTATCCAGACA	640
Petauroides volans	CCTCAGATAATCGCTTTCAGAAGAAATGTGTATCCAGACA	614
Tarsipes rostratus	CCTGAGATAATCACTTTCGGAAGAAGTGTGTATCGAGACA	623
Vombatus ursinus	CCTCAGATAATCACTTTTAGAAGAAATATGTATCCAGACA	639

	650 660 670 680	
Petaurus breviceps	CGGCGTTTTTCGTTGTTGTCGCCTCCTAAAAATGTGTGTA	644
Acrobates pygmaeus	CTGCGTTTTTC--TGTTGTCGCCTCCTAAAAA-----ATA	672
Distoechurus pennatus	AGGCATTTTTCGCTGTTGTCGCCTCCTAAAAATGTGTATA	671
Dactylopsila trivirgata	CGGCGTTTTTCGTTGTTGTCGCCTCCTAAAAATGTGTATA	680
Macropus giganteus	CGGCGTTTTTCGTTGTTGTCGCCTCCTAAAAATGTGTATA	678
Pseudochirops archeri	CGGCGTTTTTCGTTGTTGTCGCCTCCTAAAAATGTGTATA	680
Phascolarctos cinereus	CG---------GATGTTGTCGCCTCCTAAAAATGTGTATA	671
Pseudochirops corinnae	CGGCGTTTTTCGTTGTTGTCGCCTCCTAAAAATGTGTATA	680
Pseudochirops cupreus	CGGCGTTTTTCGTTGTTGTCGCCTCCTAAAAATGTGTATA	680
Phalanger gymnotis	CGGTGTTTTTCGTTCCTGTCGACTCCTAAAAATGTGTATA	661
Pseudocheirus occidentalis	CGGCTTTTTTCGTTGTTGTCGCCTCCTAAAAATGTGTATA	679
Pseudocheirus peregrinus	CGGCTTTTTTCGTTGTTGTCGCCTCCTAAAAATGTGTATA	680
Petauroides volans	CCGCGTTTTTCGTTGTTGTCGCCTCCTAAAAATGTGCATA	654
Tarsipes rostratus	CGGCGTTTTTCGTTGTTGTCGCCTCCTAAAAATGTGTATA	663
Vombatus ursinus	CGGCATTTTTCGTTGTTGTAGGCTCCTAAAAATGTGTGTA	679

	690 700 710 720	
Petaurus breviceps	TCAAGCTTCTATTTCTGTCTCCCTCCCCGTTTCCCCTCCC	684
Acrobates pygmaeus	TCAAGCTTCTATTTCTGTCTTCCCCCCGGTTTCCCCTCCC	712
Distoechurus pennatus	TCAAGCTTCTATTACTGTCTT-CCCCCGGTTTCCCCTCCC	710
Dactylopsila trivirgata	TCAAGCTTCTATTTCTGTCTTCCCCCTGGTTTCCCCTCCT	720
Macropus giganteus	TGAAGCTTCTGTTTCTGTCTCCCCCCCCGTTTCCCCTCCC	718
Pseudochirops archeri	TCAAGCTTCTATTTCTGTCTTCCCCCCGGTTTCCCCTCCC	720
Phascolarctos cinereus	TCGAGTTGCTATTTCTGTCTTCTCCCCAGTTTCCCCTCCC	711
Pseudochirops corinnae	TCAAGCTTCTATTTCTGTCTTCCCCCCGGTTTCCCCTCCC	720
Pseudochirops cupreus	TCAAGCTTCTATTTCTGTCTTCCCCCCGGTTTCCCCTCCC	720
Phalanger gymnotis	TCAAGGTTCTATTTCTGTCTTCCCCCCGGTTTCCCCTCCC	701
Pseudocheirus occidentalis	TCAAACTTATATTTCTGTCTTACCCCCGGTTTCCCCTCCC	719
Pseudocheirus peregrinus	TCAAGCTTATATTTCTGTCTTACCCCCGGTTTCCCCTCCC	720
Petauroides volans	TCAAGCTTCTATTTCTGTCTTCCCCCCGGTTTCCCCTCCC	694
Tarsipes rostratus	TAAAGCTTCTATTTCTGTCTTCCCCCCGGTTTCCCTTCCC	703
Vombatus ursinus	TCGAGTTGTTATTTCTGTCTTCCCCCCATTTTCCCCTCCC	719

	730 740 750 760	
Petaurus breviceps	GGTCTACTTTCTCTCAGGTTTCTCGGCAACCTATTCACTC	724
Acrobates pygmaeus	GGTCTACTTTCTCTCAGGTTTCTCGGCAACCTATTCATTC	752
Distoechurus pennatus	GGTCTACTTTCTCTCAGGTTTCTCGGCAACCTATTCATTC	750
Dactylopsila trivirgata	GATCTACTTTCTCTCAGGTTTCTCGGCAACCTATTCACTC	760
Macropus giganteus	GGTGTACTTTCTCTCAGGTTTCTCGGCAACCTATTCACTC	758
Pseudochirops archeri	GGTCTACTTTCTCTCAGGTTTCTCGGCAACCTATTCACTC	760
Phascolarctos cinereus	GGTCTACTTTCTCCCAGGTTTCTCGGCAACCTATTCACTC	751
Pseudochirops corinnae	GGTCTACTTTCTCTCAGGTTTCTCGGCAACCTATTCACTC	760
Pseudochirops cupreus	GGTCTACTTTCTCTCAGGTTTCTCGGCAACCTATTCACTC	760
Phalanger gymnotis	GGTCTACTTTCTCTCAGGTTTCTCGGCAACCTATTCACTC	741
Pseudocheirus occidentalis	GGTCTACTTTCTCTCAGGTTTCTCAGCAACCTATTCACTC	759
Pseudocheirus peregrinus	GGTCTACTTTCTCTCAGGTTTCTCAGCAACCTATTCACTC	760
Petauroides volans	GGTCTACTTTCTCTCAGGTTTCTCGGCAACCTATTCACTC	734
Tarsipes rostratus	GGTCTACTTTCTCTCAGGTTTCTCGGCAACCTATTCACTC	743
Vombatus ursinus	GGTCTACTTTCTCCTAGGTTTCTCGGCAACCTATTCACTC	759

	770 780 790 800	
Petaurus breviceps	AACACAAGCATTTCTTCGTCCTTAAGATAAATTACACCGC	764
Acrobates pygmaeus	AACACAAGCATTTCTGCGTCCTTAAGATAAATTACACCGC	792
Distoechurus pennatus	AACACAAGCATTTCTGCGTCCTTAAGATAAATTACACCGC	790
Dactylopsila trivirgata	AACACAAGCATTTCTGCGTCCTTAAGATAAATTACACCGC	800
Macropus giganteus	AACACAAGCATTTCTGCGTCCTTAAGATAAATTACACCGC	798
Pseudochirops archeri	AACACAAGCATTTCTGCGTCCTTAAGATAAATTACACCGC	800
Phascolarctos cinereus	AACACAAGCATTTCTGCGTCCTTAAGATAAATTACAACGC	791
Pseudochirops corinnae	AACACAAGCATTTCTGCGTCGTTAAGATAAATTACACCGC	800
Pseudochirops cupreus	AACACAAGCATTTCTGCGTCGTTAAGATAAATTACACCGC	800
Phalanger gymnotis	AACACAAGCATTTCTGCGTCCTTAAGATAAATTACACTGC	781
Pseudocheirus occidentalis	AACACAAGCATTTCTGCATCCTTAAGATAAATTACACCGC	799
Pseudocheirus peregrinus	AACACAAGCATTTCTGCGTCCTTAAGATAAATTACACCGC	800
Petauroides volans	AATACAAGCATTTCTGCGTCCTTAAGATAAATTACACCGC	774
Tarsipes rostratus	AACACAAGCATTTCTGCGTCCTTAAGATAAATTACACCGC	783
Vombatus ursinus	AACACAAGCATTTCTGCGTCCTTAAGATAAATTACACCGC	799

	810 820 830 840	
Petaurus breviceps	CTCGGCGTAATAGATTTAAAGTACGCCAGATTTTTTTTTT	804
Acrobates pygmaeus	CTTGTCGTAATAGATTTAAAGTACGCCAGGTTTTTTTTTT	832
Distoechurus pennatus	CTCGTCGTAATAGATTTAAAGTACGCCAGA---TTTTTTT	827
Dactylopsila trivirgata	CTCGGCGTAATAGATTTAAAGTACGCCAGA-TTTTTTTAT	839
Macropus giganteus	CTCGGCGTAATAGATTTAAAGTACGCCAGATTTTTTTTTT	838
Pseudochirops archeri	CTCGGCGTAATAGATTTAAAGTACGCCAGATTTTTTTTTT	840
Phascolarctos cinereus	CTCGGCGTAATAGATTTAAAGTACGCCAGATTTTTTTGTT	831
Pseudochirops corinnae	CTCGGCGTAATAGATTTAAAGTACGCCAGATTTTTTTTTT	840
Pseudochirops cupreus	CTCGGCGTAATAGATTTAAAGTACGCCAGATTTTTTTTTT	840
Phalanger gymnotis	CTCGGCGTAATAGATTTAAAGTACGCCAGATTTTTTTTTT	821
Pseudocheirus occidentalis	CTCAGCGTAATAGATTTAAAGTACGCCAGATTTTTTTTTC	839
Pseudocheirus peregrinus	CTCAGCGTAATAGATTTAAAGTACGCCAGATTTTTTTTTC	840
Petauroides volans	CTCGGCGTAATAGATTTAAAGTACGCCAGATTTTTTTAAA	814
Tarsipes rostratus	CTCGGCGTAATAGATTTAAAGTATGCCAGATTTTTTTTTT	823
Vombatus ursinus	CTCGGTGTAATAGATTTAAAGTACGCCAGATTTTTTTTAT	839

	850 860 870 880	
Petaurus breviceps	ATAATTAATTGTAAACGTGAAAAATACCTGCTGGTACTTC	844
Acrobates pygmaeus	ATAATTAATTGTAAACGTGAAAAATACCTGCTGGTACTTC	872
Distoechurus pennatus	ATAATTAATTGTAAACGTGAAAAATACCTGCTGGTACTTC	867
Dactylopsila trivirgata	ATAATTAATTGTAAACGTGAAAAATACCTGCTGGTACTTC	879
Macropus giganteus	ATAATTAATTGTAAACGTGAAAAATACCTGCTGGTACTTC	878
Pseudochirops archeri	ATAATTAATTGTAAACGTGAAAAATACCTGCTGGTACTTC	880
Phascolarctos cinereus	ATAATTAATTGTAAACGTGAAAAATACCTGCTGGTACTTC	871
Pseudochirops corinnae	ATAATTAATTGTAAACGTGAAAAATACCTGCTGGTACTTC	880
Pseudochirops cupreus	ATAATTAATTGTAAACGTGAAAAATACCTGCTGGTACTTC	880
Phalanger gymnotis	ATAATTAATTGTAAACGTGAAAAATACCTGCTGGTACTTC	861
Pseudocheirus occidentalis	ATAATTAATTGTAAACGTGAAAAATACCTGCTGGTACTTC	879
Pseudocheirus peregrinus	ATAATTAATTGTAAACGTGAAAAATACCTGCTGGTACTTC	880
Petauroides volans	ATAATTAATTGTAAACGTGAAAAATACCTGCTGGTACTTC	854
Tarsipes rostratus	ATAATTAATTGTAAACGTGAAAAATACCTGCTGGTACTTC	863
Vombatus ursinus	ATAATTAATTGTAAACGTGAAAAATACCTGCTGGTACTTC	879

	890 900 910 920	
Petaurus breviceps	ACTTTAGAGGAATGTAATTGGAAAAAAAAAAAAAAAGGAA	884
Acrobates pygmaeus	ACTTTAGAAGAATGTAATTGGCAAAAAGAAAAAAAAAGAA	912
Distoechurus pennatus	ACTTTAGAAGAATGTAATTGGCAAAAAAAAAAAAAAGGAA	907
Dactylopsila trivirgata	ACTTTAGAAGAATGTAATTGGCAAAAAAAAAAAAAAGGAA	919
Macropus giganteus	ACTTTAGAAGAATGTAATTGGCAAAAAAAAAAAAAAGGAA	918
Pseudochirops archeri	ACTTTAGAAGAATGTAATTGGCAAAAAAAAAAAAAAGGAA	920
Phascolarctos cinereus	ACTTTAGAAGAATGTAATTGGC-AAAAAAAAAAAAAGGAA	910
Pseudochirops corinnae	ACTTTAGAAGAATGTAATTGGCAAAAAAAAAAAAAAGGAA	920
Pseudochirops cupreus	ACTTTAGAAGAATGTAATTGGCAAAAAAAAAAAAAGGAAA	920
Phalanger gymnotis	ACTTTAGAAGAATGTAATTGGCAAAAAAAAAAAAAGGGAA	901
Pseudocheirus occidentalis	ACTTTAGAAGAATGTAATTGGCAAAAAAAAAAGGAAAAAA	919
Pseudocheirus peregrinus	ACTTTAGAAGAATGTAATTGGCAAAAAAAAAAGGAAAAAA	920
Petauroides volans	ACTTTAGAAGAATGTAATTGGCAAAAAAAAAAAAAGGAAA	894
Tarsipes rostratus	ACTTTAGAAGAATGTAATTGGC-AAAAAAAAAAAAAGGGA	902
Vombatus ursinus	ACTTTAGAAGAATGTAATTGGCAAAAAAAAAAAAAAGGAA	919

	930 940 950 960	
Petaurus breviceps	AAAAGAGGCGGCTGATGAATAGATAATAGATCTTTAACTA	924
Acrobates pygmaeus	AAAAGAGGCGGCTGATGAATAGATAATAGATCTTTAACTA	952
Distoechurus pennatus	AAAAGAGGCGTCTGATGAATAGATAATAGATCTTTAACTA	947
Dactylopsila trivirgata	AAAAGAGGCGGCTGATGAATAGATAATAGATCTTTAACTA	959
Macropus giganteus	AAAAGAGGCGGCTGATGAATAGATAATAGATCTTTAACTA	958
Pseudochirops archeri	AAAAGAGGCGGCTGATGAATAGATAATAGATCTTTAACTA	960
Phascolarctos cinereus	AAAAGAGGCGGCTGATGAATAGATAATAGATCTTTAACTA	950
Pseudochirops corinnae	AAAAGAGGAGGCTGATGAATAGATAATAGATCTTTAACTA	960
Pseudochirops cupreus	AAAAGAGGCGGCTGATGAATAGATAATAGATCTTTAACTA	960
Phalanger gymnotis	AAAAGAGGCGGCTGATGAATAGATAATAGATCTTTAACTA	941
Pseudocheirus occidentalis	AAGAGAAGCGGCTGATGAATAGATAATAGATCTTTAACTA	959
Pseudocheirus peregrinus	AAGAGAAGCGGCTGATGAATAGATAATAGATCTTTAACTA	960
Petauroides volans	AAAAGAGGCGGCTGATGAATAGATAATAGATCTTTAACTA	934
Tarsipes rostratus	AAAAGAGGGGGCTGATGAATAGATAATAGATCTTTAACTA	942
Vombatus ursinus	AAAAGAGGCGGCTGATGAATAGATAATAGATCTTTAACTA	959

Species	970 980 990 1000	
Petaurus breviceps	CCAATAAACAGTAAGCTGCAGCCAGCAGCCTCGGGATGTG	964
Acrobates pygmaeus	CCAATAAACAGTAAGCTGCAGCCAGCAGCCTCGGGATGTG	992
Distoechurus pennatus	CCAATAAACAGTAAGCTGCAGCCAGCAGCCTCGGGATGTG	987
Dactylopsila trivirgata	CCAATAAACAGTAAGCTGCAGCCAGCAGCCTCGGGATGTG	999
Macropus giganteus	CCAATAAACAGTAAGCTGCAGCCAGCAGCCTCGGGATGTG	998
Pseudochirops archeri	CCAATAAACAGTAAGCTGCAGCCAGCAGCCTCGGGATGTG	1000
Phascolarctos cinereus	CCAATAAACAGTAAGCTGCAGCCAGCAGCCTCGGGATGTG	990
Pseudochirops corinnae	CCAATAAACAGTAAGCTGCAGCCAGCAGCCTCGGGATGTG	1000
Pseudochirops cupreus	CCAATAAACAGTAAGCTGCAGCCAGCAGCCTCGGGATGTG	1000
Phalanger gymnotis	CCAATAAACAGTAAGCTGCAGCCAGCAGCCTCGGGATGTG	981
Pseudocheirus occidentalis	CCAATAAACAGTAAGCTGCAGCCAGCAGCCGCGGGATGTG	999
Pseudocheirus peregrinus	CCAATAAACAGTAAGCTGCAGCCAGCAGCCGCGGGATGTG	1000
Petauroides volans	CCAATAAACAGTAAGCTGCAGCCAGCAGCCTCGGGATGTG	974
Tarsipes rostratus	CCAATAAACAGTAAGCTGCAGCCAGCAGCCTCGGGATGTG	982
Vombatus ursinus	CCAATAAACAGTAAGCTGCAGCCAGCAGCCTCGGGATGTG	999

Species	1010 1020 1030 1040	
Petaurus breviceps	ATCATAATTATTCCTCGGCGCGAACCAATGGGGATGAGGG	1004
Acrobates pygmaeus	ATCATAATTATTCCTCGGCGCTAACCAATAGGGATGAGGG	1032
Distoechurus pennatus	ATCATAATTATTCCTCGGCGCGAACCAATGGGGATGAGGG	1027
Dactylopsila trivirgata	ATCATAATTATTCCTCGGCGCGAACCAATGGGGATGAGGG	1039
Macropus giganteus	ATCATAATTATTCCTCGGCGCGAACCAATGGGGATGAGGG	1038
Pseudochirops archeri	ATCATAATTATTCGTCGGCGCGAACCAATGGGGATGAGGG	1040
Phascolarctos cinereus	ATCATAATTATTCCTCGGCGCGAACCAATGGGGATGAGGG	1030
Pseudochirops corinnae	ATCATAATTATTCGTCGGCGCGAACCAATGAGGATGAGGG	1040
Pseudochirops cupreus	ATCATAATTATTCGTCGGCGCGAACCAATGGGGATGAGGG	1040
Phalanger gymnotis	ATCATAATTATTCCTCGGCGCGAACCAATAGGGATGAGGG	1021
Pseudocheirus occidentalis	ATCATAATTATTCCTCGGCGCGAACCAATGGCGATGAGGG	1039
Pseudocheirus peregrinus	ATCATAATTATTCCTCGGCGCGAACCAATGGCGATGAGGG	1040
Petauroides volans	ATCATAATTATTCCTCGGAGTGAACCAATGGGGATGAGGG	1014
Tarsipes rostratus	ATCATAATTATTCCTCGGCGCTAACCAATGGGGATGAGGG	1022
Vombatus ursinus	ATCATAATTATTCCTCGGCGCGAACCAATGGGGATGAGGG	1039

Species	1050 1060 1070 1080	
Petaurus breviceps	AACTCAGCCCTAAAATCATCCCAAAACCAAAGGCGAACTC	1044
Acrobates pygmaeus	AACTCAGCCCTAAAATCATCCCAAAACCAAAGGCGAACTC	1072
Distoechurus pennatus	AACTCAGCCCTAAAATCATCCCAAAACCAAAGGCGAACTC	1067
Dactylopsila trivirgata	AACTCAGCCCTAAAATCATCCCAAAACCAAAGGCGAACTC	1079
Macropus giganteus	AACTCAGCCCTAAAATCATCCCAAAACCAAAGGCAAACTC	1078
Pseudochirops archeri	AACTCAGCCCTAAAATCATCCCAAAACCAAAGGCGAATTC	1080
Phascolarctos cinereus	AGCTCAGCCCTAAAATCATCCCAAAACCAAAGGCGAGCTC	1070
Pseudochirops corinnae	AACTCAGCCCTAAAATCATCCCAAAACCAAAGGCGAACTC	1080
Pseudochirops cupreus	AACTCAGCCCTAAAATCATCCCAAAACCAAAGGCGAACTC	1080
Phalanger gymnotis	AACTCAGCCCTAAAATCATCCCAAAACCAAAGGCGAACTC	1061
Pseudocheirus occidentalis	AACTCAGCCCTAAAATCATCCCAAAACCAAAGGCGAACTC	1079
Pseudocheirus peregrinus	AACTCAGCCCTAAAATCATCCCAAAACCAAAGGCGAACTC	1080
Petauroides volans	AACTCAGCCCTAAAATCATCCCAAAACCAAAGGCGAACTC	1054
Tarsipes rostratus	AGCTCAGCCCTAAAATCATCCCAAAACCAAAGGCGAACTC	1062
Vombatus ursinus	AACTCAGCCCTAAAATCATCCCAAAACCAAAGGCGAACTC	1079

	1090 1100 1110 1120	
Petaurus breviceps	GAAAATACGGACAAGGTCTGGAGCCTGAAATCCAACTCAC	1084
Acrobates pygmaeus	GAAAATACGGACAAGGTCTGGAGCCTGAGATCCAACTCAC	1112
Distoechurus pennatus	GAAAATACGGACAAGGTCTGGAGCCTGAGATCCAACTCA-	1106
Dactylopsila trivirgata	GAAAATACGGACAAGGTCTGCAGCCTGAGCTCCAACTCAC	1119
Macropus giganteus	GAAAATACGGACAAGGTCTGGAGCCTGAGATCCAACTCAT	1118
Pseudochirops archeri	GAAAATACGGACAAGGTCTGGAGCCTGAGATCCAACTCAC	1120
Phascolarctos cinereus	GAAAATACGGACAAGGTCTGGAGCCTGAGATCCAACTCAC	1110
Pseudochirops corinnae	GAAAATACGGACAAGGTCTGGAGCCTGAGATCCAACTCAC	1120
Pseudochirops cupreus	GAAAATACGGACAAGGTCTGGAGCCTGAGATCCAACTCAC	1120
Phalanger gymnotis	GAAAATACGGACATGGTCTGGAGCCTCAGATCCAACTCAC	1101
Pseudocheirus occidentalis	GAAAATACGGACAAGGTCTGGAGCCTGAGATCCAACTCAC	1119
Pseudocheirus peregrinus	GAAAATACGGACAAGGTCTGGAGCCTGAGATCCAACTCAC	1120
Petauroides volans	GAAAATACGGACAAGGTCTGGAGCCTGAGATCCAACTCAC	1094
Tarsipes rostratus	GAAAATACGGACAAGGTCTGATGCATAAGATCCAACTCA-	1101
Vombatus ursinus	GAAAATACGGACAAGGTCTGGAGCCTGAGATCCAACTCAC	1119

	1130 1140 1150 1160	
Petaurus breviceps	---TTTCTAGTCGTCTTCCCGACTGAAGCCCGAGATTTAA	1121
Acrobates pygmaeus	CTGTTTCTAGTCTTCTCCAAGGCTGAAGCCTGAGATTTAA	1152
Distoechurus pennatus	CTGTTTCTAGTCCTCTCCCCGACTGAAGCCTGAGATTTAA	1146
Dactylopsila trivirgata	CTGTTTCTAGTCCTCTCCCCGATTGAAGCCCGAGATTTAA	1159
Macropus giganteus	CCATTCCTAGTCTTTTCCCCAACTGAAGCCTGAGATTTAA	1158
Pseudochirops archeri	CTGTTTCTAGTCCTCTCCCCGACTGAAGGCTGAGATTTAA	1160
Phascolarctos cinereus	CTGTGCCTAGTCCTCTCCCTGACTGAAGCCTGAGATTCAA	1150
Pseudochirops corinnae	CTGTTTCTAGTCCTCTCCCCGACTGAAGGCTGAGATTTAA	1160
Pseudochirops cupreus	CTGTTTCTAGTCCTCTCCCCGACTGAAGGTTGAGATTTAA	1160
Phalanger gymnotis	CCATTCCTAGTCCTTTCCCCGACTGAAGCCTGAGATTTAA	1141
Pseudocheirus occidentalis	CTGTTTCTAGTCCTCTCCCCGACTGAAGCCAGAGATTTAA	1159
Pseudocheirus peregrinus	CTGTTTCTAGTCCTCTCCCCGACTGAAGCCAGAGATTTAA	1160
Petauroides volans	CTGTTTCTAGTCCTCTCCCCGACTGAAGCCTGAGATTTAA	1134
Tarsipes rostratus	CTGTTTTTAGTCCTCTCCCCGACTGAAGCCGGAGATTTAA	1141
Vombatus ursinus	CTGTGCCTAGTCCTCTCCCTGACTGAAGCCTGAGATTCAA	1159

	1170 1180 1190 1200	
Petaurus breviceps	CTCACACTGCTCCTGATCCTCT-CCAAACATAGCAATTAA	1160
Acrobates pygmaeus	CTCACACTGCTCCTGGTCCTCTCCCAAACATAGCAATTAA	1192
Distoechurus pennatus	GT--CACTGCTCCTGGTCCTCTCCCAAACACAGCAATTAA	1184
Dactylopsila trivirgata	CTCACACTGCTCCTGATCCTCT-CCAAACATAGCAATTAA	1198
Macropus giganteus	CTCACACCGCTCCTGGTCCTCTCCCAGACACAGCAATGAA	1198
Pseudochirops archeri	CTCACACTGCTCCTGGTCCTCTCCCAAACATAGCAATTAA	1200
Phascolarctos cinereus	CTCACCCTGCTCCTGGTCCTCTCCCAGACATAGCAATTAA	1190
Pseudochirops corinnae	CTCACACTGCTCCTGGTCCTCTCCCAAACGTAGCAATTAA	1200
Pseudochirops cupreus	CTCACACTGCTCCTGGTCCTCTCCCAAACATAGCAATTAA	1200
Phalanger gymnotis	CTCACAATGCTCCTGGTCCTCTCCCAAACATAGCAATTAA	1181
Pseudocheirus occidentalis	CTCACACTGCTCCAAGTCCTCTCCCAAACATAGCAATTAA	1199
Pseudocheirus peregrinus	CTCACACTGCTCCAAGTCCTCTCCCAAACATAGCAATTAA	1200
Petauroides volans	CTCACACTGCTCCAGGTCCTCTCCCAAACATAGCAATGAA	1174
Tarsipes rostratus	CTCACTCTGCTCCTGGTCCTTTCCCAAACATAGCAATTAA	1181
Vombatus ursinus	CTCACACCGCTCGTGGTCCTCTCCCAGACATAGCAATTAA	1199

	1210 1220 1230 1240	
Petaurus breviceps	CAATCCAGGGTGGGACATAGTGGTAGATGCTTTAATACAT	1200
Acrobates pygmaeus	CAATCGAAGGTGGGGCATAGTGGTAGATGCTTTAATAAAT	1232
Distoechurus pennatus	CAATCCAGGGTGGGGCATAGTGGTAGATGCTTTAATAAAT	1224
Dactylopsila trivirgata	CAATCCAGGGTGGGGCATAGTGGTAGATGCTTTAATAAAT	1238
Macropus giganteus	CAATCCAGGGTGAGGCATGGTGCTGGATGCTTTAATAAAT	1238
Pseudochirops archeri	CAATCCAGGGTGGGGCATAGTGGTAGATGTTTTAATAAAT	1240
Phascolarctos cinereus	CAATCCAGGGTGGGGCATAGTGGTAGATACTTTAATAAAT	1230
Pseudochirops corinnae	CAATCTAGGGTGGGGCACAGTGGTAGATGTTTTAACAAAT	1240
Pseudochirops cupreus	CAATCCAGGGTGGGGCATAGTGGTAGATGTTTTAACAAAT	1240
Phalanger gymnotis	CAATCCAGGGTGGGGCATAGTGGTAGATGCTTTAATAAAT	1221
Pseudocheirus occidentalis	CAATCCAGGGTAGGGCATAGAGGTAGATGTTTTAATAAAT	1239
Pseudocheirus peregrinus	CAATCCAGGGGAGGGCATAGAGGTAGATGTTTTAATAAAT	1240
Petauroides volans	CAATCCAGGGTGGGGCATAGTGGTAGATGCTGTAATAAAT	1214
Tarsipes rostratus	CAATCCAGGGTGGGGCATAGAGGTGGCTGCTTTAATAAAT	1221
Vombatus ursinus	CAATCCAGGGTGGGGCATGGTGGTAGATGCTTTAATAAAT	1239

	1250 1260 1270 1280	
Petaurus breviceps	GACGTTAGGAACAACAATAATAACAAAGATAATAAGAATG	1240
Acrobates pygmaeus	GACATTAGCAACAACAACAATAACAAAGATAATAATAATG	1272
Distoechurus pennatus	GGCATTAGCAACAACAATAATAACAAAGATAATAATAATG	1264
Dactylopsila trivirgata	GACATTAGTAACAACAATACTAACAAAGATAATAAGAATG	1278
Macropus giganteus	GGCATTAGCAACAACATTAATAACAAAGATAATAATAATG	1278
Pseudochirops archeri	GACATTAGTAACAATAATAATAAGAAAGATAATAATAATG	1280
Phascolarctos cinereus	TACATTAGCAACAACAA------------TAATAACAATG	1258
Pseudochirops corinnae	GACATTAGTAACAATATTAATAACAAAGATAATAATAATG	1280
Pseudochirops cupreus	GACATTAGTAACAATAATAATAACAAAGATGACGATAATG	1280
Phalanger gymnotis	GACATTAGCAACAGCAATAATAACAAATATAATAATGATG	1261
Pseudocheirus occidentalis	GACATTAGTAACAACAATAATAACAAAGATAATAATAATG	1279
Pseudocheirus peregrinus	GACCTTAGTAACCACAATAATCACAAAGAGAATAATAACG	1280
Petauroides volans	GACATTAGTAACAACAATAATAACAAAGATAATAATAATG	1254
Tarsipes rostratus	GACATTTGTAAGAACAA-----------------------	1238
Vombatus ursinus	TGCATTAGCAACAACAATAATAACAAAGATAATAACAATG	1279

	1290 1300 1310 1320	
Petaurus breviceps	ATCGCTAAGACACAAACAGCATCTACCTCAACAATGCAGA	1280
Acrobates pygmaeus	ACCGCTAAGACTCAAACTGCCTCTACCTC-ACAGTACAGA	1311
Distoechurus pennatus	ACCGCTAAGTCACAAACTGCATCTACCTTGACAGTACAGA	1304
Dactylopsila trivirgata	ATCGCTAAGACACAAACCGCATCTACCTCGACAGTACAGA	1318
Macropus giganteus	ACCGCTAAGACACAAACTGCATCTACCTCCACTATACAGA	1318
Pseudochirops archeri	ACCGCTAAGACACAAACCGCATCTACCTCGACAGTACAGA	1320
Phascolarctos cinereus	ACCGCTAAGGCATAAACTGCTTCTACCTCCACAGTAGAGA	1298
Pseudochirops corinnae	ACCGCTAAGACACAAACCACATCTACCTCGACAGTACAGA	1320
Pseudochirops cupreus	ACCGCTAAGACACAAACCGCATCTACCTCGACAGTACAGA	1320
Phalanger gymnotis	ACCGCTAAGACACAAACTGCATCTACCTCCACAGTACAGA	1301
Pseudocheirus occidentalis	ACCGCTAAGACACAAACCGCATCTACCTCGACAGTACGA-	1318
Pseudocheirus peregrinus	---------------ACCGCATCTACCTCGACAGTACGA-	1304
Petauroides volans	ACCGCTAAGACACAAACCGCATCTACCTCAACAGGGCAGA	1294
Tarsipes rostratus	-------------ATACCACATCTACCTCGACAGTCCGGA	1265
Vombatus ursinus	ACCGCTAAGACACAAACTGCTTCTACCTCCACAGTACAGA	1319

	1330 1340 1350 1360	
Petaurus breviceps	CTTTCCCCCAGAACTTTCGAAGGAATCATTTGTATTTGTG	1320
Acrobates pygmaeus	CTTTCCCCCAGAACTTTCGAAGGAAGCGTTTGTATGTGTG	1351
Distoechurus pennatus	CTTTCCCCCAGAACTTTCGAAAGAAGCGTTTGTATGTGTG	1344
Dactylopsila trivirgata	CTTTACCCCAGAACTTTCGAAGGAAGCATTTGTATGTGTG	1358
Macropus giganteus	CTTTTCCCCAGGCCTTTCAAAGGAAGCGTTTGTCTGTGTG	1358
Pseudochirops archeri	CTTTGCCCCAGAACTTTCGCAGGAAGCATTTGTATGTGTG	1360
Phascolarctos cinereus	CTTTTCCCCAAAACTTTCGAAAGAAGCGTTTGTACGTGTG	1338
Pseudochirops corinnae	CTCTGCCCCAGAACTTTCGAAGGAAGCATTTGTATGTGTG	1360
Pseudochirops cupreus	CTTTGCCCCAGAACTTTGGAAGGAAGCATTTGTATGTGTG	1360
Phalanger gymnotis	CTTTTCCCCAGAACTTTCGAAGGAAGCGTTTGTATGTGTG	1341
Pseudocheirus occidentalis	-TTTGCCCCAGAACTTTTGAAGGAAGCATTTGTATGTGTG	1357
Pseudocheirus peregrinus	-TTTGCCCCAGAACTTTTGAAGGAAGCATTTGTATGTGCG	1343
Petauroides volans	C--TGCCCCAGAACTTTCGAAGGAAGCATTTGTATGTGTG	1332
Tarsipes rostratus	-----CCCCAGGACTTTCGAAGGAAGCATTTGTACATGTG	1300
Vombatus ursinus	CTTTTCCCCAGAACTTTCGAAAGAAGTGTTTGTATGTGTG	1359

	1370 1380 1390 1400	
Petaurus breviceps	ATGGTGGAGGGGGTGAGTTTGTGGAGGGTAGAGGTTGCAA	1360
Acrobates pygmaeus	ATGGTGGAGGGGGTGTGATTGTGGAGGGTAGAGGTCATAA	1391
Distoechurus pennatus	GTGGTGGAGGGGGGGTGTTTGTGGAGGGTAGAGGCCGTAA	1384
Dactylopsila trivirgata	AAGGTGGAGGGGGTGTGTTTGTGGAGGGTAGAGGTCGCAA	1398
Macropus giganteus	ACGGTGGAGGGAGTGTGTTTGAGAAGGGTAGAGGTGGTAA	1398
Pseudochirops archeri	ATGGTGGAGGGGGTGTGTTTATAGAGGGTAGAGGTCTCAA	1400
Phascolarctos cinereus	ATGGTGGAGGGGGTGTGTGTGTGGAGAGTAGAGGTCGTAA	1378
Pseudochirops corinnae	ATGGTGGAGGGGGTGTGTTTGTAGAGGGTAGAGGTCTCAA	1400
Pseudochirops cupreus	ATGGTGGAGGGGGTGTGTTTGTAGAGGGTAGAGGTCTCAA	1400
Phalanger gymnotis	ATGGTGGAGGGTGTGTGTTTGTGGGGGGTAGAGGTCGTAA	1381
Pseudocheirus occidentalis	ATGGTGGAGGGGGTGTGTTTGTAGAGGGTAGAGGTCTCAA	1397
Pseudocheirus peregrinus	ATGGTGGAGGGGGTGTGTTTGTAGAGGGTAGAGGTCTCAA	1383
Petauroides volans	ATGGTGGAGGGCGTATGTCTGTAGAGGGTAGAGGTCTCA-	1371
Tarsipes rostratus	ATGGAGGAGGGGGTGTGTTTGTGGAGGGGAGAAGTCTCAA	1340
Vombatus ursinus	ATGGGGGAGGGTGTGTGTGTGTGGAGGGTAGAGGTCGTAA	1399

	1410 1420 1430 1440	
Petaurus breviceps	TCACCACCCAAGGAAAAAAAAATGTGACTTGAGAAAGAGA	1400
Acrobates pygmaeus	TCACCACCCAGGGAAAAAAAAGTTTGACTTGATTAAGAGA	1431
Distoechurus pennatus	TCACCACCCAGAAGAAAAAAAGTGTGACTAGATTCAAAGA	1424
Dactylopsila trivirgata	GCACCACCCAAGGAAAAAAAAATGTGACTTCATTAAGAGA	1438
Macropus giganteus	TCACCACTAAGGAGGAAAAAAGTGTGACTTGATTAAGAGA	1438
Pseudochirops archeri	TCACCACCCAGGGAAAAAAAAGTGTGACTTGATTAAGAGA	1440
Phascolarctos cinereus	TCACCACCCAGAAGAAAAAAAGTCTGATTTGATTAAGAGA	1418
Pseudochirops corinnae	TCACCACCCAGGGAAAAAAAAAAGTGACTTGATTAAGAGA	1440
Pseudochirops cupreus	TCACCACCCAGGGGAAAAAAA--GTGACTTGATTAAGAGA	1438
Phalanger gymnotis	TCACCACCCAGGAAAAAAAAAGTGTTACTTGATTGAGAGA	1421
Pseudocheirus occidentalis	TCACCACCCAGGGGAACAAAAGTGTGACTTGATTAAGAGA	1437
Pseudocheirus peregrinus	TCACCACCCAGGGGAACAAAAGTGTGACTTGATTAAGAGA	1423
Petauroides volans	--ACCACCCAGGGGGAAAAAAATGTGACTTGATTAAGAGA	1409
Tarsipes rostratus	TCACCACCCAGGAGGAAAAACAAGGGACTTGATGAAAAGA	1380
Vombatus ursinus	TCACCACCCAGGAGAAAAAAAGTGTGATTTGATTAAGAGA	1439

Species	1450 · 1460 · 1470 · 1480	
Petaurus breviceps	AGGGACAAACTAGTAGTTTGCTCTAGTTGCTCTTCGAA-G	1439
Acrobates pygmaeus	AGGGACAAGCTAGCAGTAGGCTCTAGTTGCCCTTCGAAGG	1471
Distoechurus pennatus	AGGGACAAACTAGCAGTAGGCTCTAGTTGCCCTTCGAAGG	1464
Dactylopsila trivirgata	AGGGACAAACTAGTACTTTGCTCTAGTTACCCTTCGAAGG	1478
Macropus giganteus	AGGGACAAGCTAACAGTAGGCTTGGGTTGCACTTTGAAGG	1478
Pseudochirops archeri	AAGGACAAACGAGTAGTTTGCTCTAGTTGCCCTTCGGAGG	1480
Phascolarctos cinereus	AGCGACAAGCTAGCAGTAAGCTCTGGTTGCCCTTTGAAGG	1458
Pseudochirops corinnae	AAGGACAAACGAGTAGTTTGCTCTAGTTGCCCTTCGGAGG	1480
Pseudochirops cupreus	AAGGACAAACGAGTAGTTTGCTCTAGTTGCCCTTCGGAGG	1478
Phalanger gymnotis	ACGGACAAGCTAGCAGTAGGCTCCGGTTGCCCTTTGAAGG	1461
Pseudocheirus occidentalis	AAGGACAAACCAGTAGTCTGCTCTAGTTGCCCTACGGAGG	1477
Pseudocheirus peregrinus	AAGGACAAACCAGTAGTCTGCTCTAGTTGCCCTACGGAGG	1463
Petauroides volans	AAGGACAAACCAGTAGTTTGCTCTAATTGCCCTTCGGAGG	1449
Tarsipes rostratus	AGGGACAAACTAGAAGTAGGCTCTGGTTGCCCTTCGAAGG	1420
Vombatus ursinus	AGGGACAAGCTAGCAGTAAGCTCTGGTTGCCCTTTGAAGG	1479

Species	1490 · 1500 · 1510 · 1520	
Petaurus breviceps	GGGCCCGATGGTGTCGATTTGCCAGCTACCTAAAAGGGCA	1479
Acrobates pygmaeus	GGGTGCAATAGTATCGATTTGTCAATTGCCTAAAAGGGCA	1511
Distoechurus pennatus	GGATGCAATAGTGTCGATTTGCCAACTGCCTAAAAGGGCA	1504
Dactylopsila trivirgata	GGGTGCGATAATGTCGATTTACCAACTACCTAAAAGGGCA	1518
Macropus giganteus	GGGTGCGATAGTGTCGATTTGCCAACTACCTAACAGGGCA	1518
Pseudochirops archeri	GGGTGCGATGGTGTCGATTTGCCAACTACCTAAAAGGTCA	1520
Phascolarctos cinereus	GGGTGCACTGGTGTCGATCTGCCAACTACCTAACAGGGCA	1498
Pseudochirops corinnae	GGGTGCGATGGTGTCGATTTGCCAACTACCTAAAAGGTCA	1520
Pseudochirops cupreus	GGGTGCGATGGTGTCGATTTGCCAACTACCTAAAAGGTCA	1518
Phalanger gymnotis	GGGTGCGATGGTGTCGATTTGCCAACTACCTAACAGGGCA	1501
Pseudocheirus occidentalis	GGGTGCGATGGTATCGATTTGCCAACTACCTAAAAGGGCA	1517
Pseudocheirus peregrinus	GGGTGCGATGGTATCGATTTGCCAACTATCTAAAAGGGCA	1503
Petauroides volans	GGGTGCGATGGTGTCGATTTGTCAACTACCTAAAAGGGCA	1489
Tarsipes rostratus	GGGTGCGATGCTGTCGATTTGCCAACTACCTAACAGGGGA	1460
Vombatus ursinus	GGGTGCGCTGGTGTCGATTTGCCAACTACCTAACAGGGCA	1519

Species	1530 · 1540 · 1550 · 1560	
Petaurus breviceps	GGGTCTCTTAGACCCTCTCGGTCCCAGAGACCCTTTGGAA	1519
Acrobates pygmaeus	GCGTCTCTGGCACTCTCTCGATCGCAGAGACCCTTTGGAA	1551
Distoechurus pennatus	GGGTCTTAGGGACCCTCTCGATC-------CCCTTTGAAA	1537
Dactylopsila trivirgata	GGGTCTCTGGGACAGAGAGGGTCTAAGAGACTCTTTGGAA	1558
Macropus giganteus	GGCTCTCTTAGACCCTCTCGGTCCTAGAGACCCTTTGGAA	1558
Pseudochirops archeri	GGGTCTCTTAGAACCTCTCTGTCCCAGAGACCCTCTGGAA	1560
Phascolarctos cinereus	GGGTCTCTTGGACCCTCTCGGTCCCAGAGACACTTTGGAA	1538
Pseudochirops corinnae	GGATCTCTTAGACCCTCTCTGTCCCAGAGACCCTTTGGAA	1560
Pseudochirops cupreus	GGGTCTCTTAGACCCTCTCTGTCCCAGAGACCCTTTGGAA	1558
Phalanger gymnotis	GAGTCTCTTAGACCCTCTCGGTCCCAGAGACCCTTTGGAA	1541
Pseudocheirus occidentalis	GGGTCTCTTAGACCCTCTCGGTCCCAGAGACCCTTTGGAA	1557
Pseudocheirus peregrinus	GGGTCTCTTAGACCCTCTCGGTCCCAGAGACCCTTTGGAA	1543
Petauroides volans	GGGTCTCTTAGACCCTCTCGGTCCCAGAGACCCTTAGGAA	1529
Tarsipes rostratus	GCGTGCCTTAGA--CTCTCGGACCCAGAGACCCTTTGGAA	1498
Vombatus ursinus	GGGTCTCTTGGACCCTCTCGGTCCCAGAGACGCTTTGGAA	1559

```
                              1570      1580      1590      1600
Petaurus breviceps            CACAAATTTGGAAAGGAGGGAGCTAGGTAGCAGAGAAAGG  1559
Acrobates pygmaeus            CACAAGTTTGGAAAGGAGGGAACTGGGTAGCAGAGAAGGG  1591
Distoechurus pennatus         CACAAGTTTGGAAAGGAGGGAACTGGGTAGCAGAGAAGGG  1577
Dactylopsila trivirgata       CACAAGTTTGGAAAGGAGGAAGCAGGGTAGCATAGAAGGG  1598
Macropus giganteus            CACAAGTTTGTTAAGGAGGGAGCTGGGTAGCAGAAAAAGG  1598
Pseudochirops archeri         CACAAGTTTGGAAAGGAGGGAGCTGGATAGCAGAGAAGGG  1600
Phascolarctos cinereus        CACAAATTTGGAAAGGAGGGAGCTGGGTAACAGAGAAGGG  1578
Pseudochirops corinnae        CACAAGTTTGGAAAGGAGGGAGCTGGGTAGCAGAGAAGGG  1600
Pseudochirops cupreus         CAC--GTTTGGAAAGGAGGGAGCTGGGTAGCAGAGAAGGG  1596
Phalanger gymnotis            CACAAGTTTGGAAAGGAGAGAGCTGGATAGTAGAGAAGGG  1581
Pseudocheirus occidentalis    CACAAGTTTGGAAAGGAGGGAGCTGGGTAGCAGAGAAGGG  1597
Pseudocheirus peregrinus      CACAAGTTTGGAAAGGAGGGAGCTGGGTAGCAGAGAAGGG  1583
Petauroides volans            CACAAGTTTGGAAAGGAGGGAGCTGGGTAGCAGAGAAGGG  1569
Tarsipes rostratus            CACAAGTTTGGAATGGAGGGAGCTGGGTAGCGGAGAAGGG  1538
Vombatus ursinus              CTCAGATTTGGAAAGGACAGATCTGGGTAGCAGAGAAGGG  1599
```

```
                              1610      1620      1630      1640
Petaurus breviceps            GAGGGGACAGCTCAAAGAGTTTACTCTTCGCCTAGGAGAC  1599
Acrobates pygmaeus            GAGGGAACAGCTCTCAGAGTTTACTCTTCGCCTAGGAGAC  1631
Distoechurus pennatus         GAGGGGACAGCTCTCAGAGTTTACTCTTCGCCTAGGAGAC  1617
Dactylopsila trivirgata       GAGGAGACACCTCAAAGAGTTTACTCTTAGACTAGGAGAC  1638
Macropus giganteus            GAGGGGACAGCTCTCAGAGTTTACTCTTCGCCTAGGAGAC  1638
Pseudochirops archeri         GAGGGGACAGTTCCAAGAGTTTACTCTTTGCCTAGGAGAC  1640
Phascolarctos cinereus        GAGGGGACATCTCTGAGAGTTTACTCTTCGCCTAGGAGAC  1618
Pseudochirops corinnae        GAGGGGACAGCTCTAAGAGTTTACTCTTCGCCTAGGAGAC  1640
Pseudochirops cupreus         GAAGGGACAGCTCTAAGAGTTTACTCTTCGCCTAGGAGAC  1636
Phalanger gymnotis            GAGGGGACAGCTCTCAGAGTTTACTCTTCGCCTAGGAGAC  1621
Pseudocheirus occidentalis    GAGGGGACGGCTCAAAGAGTTTACTCTTCGCCTAGGAGAC  1637
Pseudocheirus peregrinus      GAGGGGACGGCTCAAAGAGTTTACTCTTCGCCTAGGAGAC  1623
Petauroides volans            GAGGGGACAGCTCAAAGAGTTTACTCTTCGCCTAGGAGAC  1609
Tarsipes rostratus            GAGGGGACAGCTCAGAGAGTTTACTCTTCCTCTAGGAGAC  1578
Vombatus ursinus              GAGGGTACATCTCTCAGAGTTTACTCTTCGCCTAGGAGAC  1639
```

```
                              1650      1660      1670      1680
Petaurus breviceps            AACATTAAGTTTTGCCCTCAGGAGGGGGAGGGGGATGGAG  1639
Acrobates pygmaeus            AACATTAAGTTTTGCCCCCGGGAGGGGGAGGGGGATGGAG  1671
Distoechurus pennatus         AACATTAAGTTTTGCCCCCGGGAGGTGGAGGGGGATGGAG  1657
Dactylopsila trivirgata       AACCTTAAGTTTTGCCCTCAGGAGGGGGAGGGGGATAGAG  1678
Macropus giganteus            AACATTTAGTTTTGCCCTCAGGAGGGGGAGGGGGATGGAG  1678
Pseudochirops archeri         AACATTAAGTTTTGCCCTCAGGAGGGGGATGGGGACGGAG  1680
Phascolarctos cinereus        AACATTAAGTTTTGCCCTCAGGAGGGGGATGGGGATGGAG  1658
Pseudochirops corinnae        AACATTAAGTTTTGCCCTTAGGAGGGGGATGGGGATGGAG  1680
Pseudochirops cupreus         AACATTAAGTTTTGCCCTCAGGAGGGGGATGGGGATGGAG  1676
Phalanger gymnotis            AACATTAAGTTTTGCCCTCAGGAGGGGGAGGGGGACGGAG  1661
Pseudocheirus occidentalis    AACATTAAGTTTTGCCCTCAGGAGGGGGATGGGGATGGAG  1677
Pseudocheirus peregrinus      AACATTAAGTTTTGCCCTCAGGAGGGGGATGGGGATGGAG  1663
Petauroides volans            AACATTAAGTTTTGCCCTCAGGAGGGGGATGGGGATGGAG  1649
Tarsipes rostratus            AACATTAAGTTTTGCCCTCAGGAGGGGGAGGGGGGTGAAG  1618
Vombatus ursinus              AACATTAAGTTTCCCCCTCAGGAGGGGGAGGGGGATGGAG  1679
```

	1690 1700 1710 1720	
Petaurus breviceps	AAATATAATAGTTTCCCCCACCCTCACCCCAAGCCAGCCC	1679
Acrobates pygmaeus	AAATATAATAGTTTCCCCCACCCTCACCCCAAGCCAGCCC	1711
Distoechurus pennatus	AAATATAATAGCTTCCCCCACCCTCACCCCAAGCCAGCCC	1697
Dactylopsila trivirgata	AAATATAATAGTTTCCCCCACCCTCACCCCAAGCCAGCCC	1718
Macropus giganteus	AAATATAATAGTTTCCCCCACCCTCACCCCAAGCCAGCCC	1718
Pseudochirops archeri	AAATATAATAGTTTCCCCCACCCTCACCCCAAGCCAGCCC	1720
Phascolarctos cinereus	AAATATAATAGTTTCCCCCACCCTTACCCCAAGCCAGCCC	1698
Pseudochirops corinnae	AAATATAATAGTTTCCCCCACCCTCACCCCAAGCCAGCCC	1720
Pseudochirops cupreus	AAATATAATAGTTTCCCCCACCCTCACCCCAAGCCAGCCC	1716
Phalanger gymnotis	AAATATAATAGTTTCCCTCACCCTTACCCCAAGCCAGCCC	1701
Pseudocheirus occidentalis	AAATATAATAGTTTCCCCCACCCTCACCCCAAGCCAGCCC	1717
Pseudocheirus peregrinus	AAATATAATAGTTTCCCCCACCCTCACCCCAAGCCAGCCC	1703
Petauroides volans	AAATATAATAGTTTCCCCCACCCTCACCCCAAGCCAGCCC	1689
Tarsipes rostratus	AAATATAATAGTTTCCCCCACCCTCACCCCAAGCCAGCCC	1658
Vombatus ursinus	AAATATAATAGTTTCCCCCACCCTCACCCCAAGCCAGCCC	1719

	1730 1740 1750 1760	
Petaurus breviceps	CCTTTAGCCCTCCCCATGAGTCCTACTCCCTCCTCCCCCG	1719
Acrobates pygmaeus	CCTTTAGCCCTCCCCATGCGTCCCACTCCCTCCTCCCCCA	1751
Distoechurus pennatus	CCTTTAGCCCTCCCCATGCGTCCCACTCCCTCCTCCCTTG	1737
Dactylopsila trivirgata	CCTTTAGCCCTCCCCATGCGTCCTACTCCCTCCTCCCCCG	1758
Macropus giganteus	CCTTTAGCCCTCCCCATGCTTCCCACTCCCTCCTCCCCCG	1758
Pseudochirops archeri	CCTTTAGCCCTCCCCATGCGTCCTGCTCCCTCCTCCCCCG	1760
Phascolarctos cinereus	CCTTTAGCCCTCCCCATGCGTCCCATTCCCTCCTCCCCCG	1738
Pseudochirops corinnae	CCTTTAGCCCTCCCCATGCGTCCTACTCCCTCCTCCCCCG	1760
Pseudochirops cupreus	CCTTTAGCCCTCCCCATGCGTCCTACTTCCTCCTCCCCCG	1756
Phalanger gymnotis	CCTTTAGCCCTCCCCATGCGTCCCACTCCCTCCT-CCCCA	1740
Pseudocheirus occidentalis	CCTTTAGCCCTCCCCATGCGTCCTACTCCCTCCTCCCCCG	1757
Pseudocheirus peregrinus	CCTTTAGCCCTCCCCATGCGTCCTACTCCCTCCTCCCCCG	1743
Petauroides volans	CCTTTAGCCCTCCCCATGCGTCCTACTCCCTCCT-CCCCG	1728
Tarsipes rostratus	CCTTTAGCCCTCCCCATGCGTCCTCCTCCCTCCTCCCCCG	1698
Vombatus ursinus	CCTTTAGCCCTCCCCATGCGTCCCACTCCCTCCTCCCCCG	1759

	1770 1780 1790 1800	
Petaurus breviceps	GGCTCAAGTCTGGAATGAGAAGCCGCGGAGCGCGCGGGAG	1759
Acrobates pygmaeus	GACTCAAGGCTGGAATGAGAAACGGCGGAGCGCGCGAGAG	1791
Distoechurus pennatus	GGCTCAAGGCTGGAATGAGAAACGGCGGAGCGCGCGGGAG	1777
Dactylopsila trivirgata	GGCTCAAGTCTGGAATGAGAAGCGGCGGAGCGCGCGGGAG	1798
Macropus giganteus	GGCTCATGTCTGGAATGAGAAGCCGCGGAGCGCGCGGGAG	1798
Pseudochirops archeri	GGCTCAAGTCTGGAATGAGAAGCGGCGGAGCGCGCGGGAG	1800
Phascolarctos cinereus	GGCTCAAGTCTGGAATGAGAAGCGGCGGAGCGCGCGGGAG	1778
Pseudochirops corinnae	GGCTCAAGTCTGGAATGAGAGGCGGCGGAGCGCGCGGGAG	1800
Pseudochirops cupreus	GGCTCAAGTCTGGAATGAGAAGCGGCGGAGCGCGCGGGAG	1796
Phalanger gymnotis	GGCTCAAGTCTGGAATGAGAAGCGGCGGAGCGCGCGGGAG	1780
Pseudocheirus occidentalis	AGCTCGAGTCTGGAATGAGAATCGGCGGAGCGCGCGGGAG	1797
Pseudocheirus peregrinus	AGCTCGAGTCTGGAATGAGAATCGGCGGAGCGCGCGGGAG	1783
Petauroides volans	GGCTCAAGTCTGGAATGAGAAGCGGCGGAGCGCGCGGGAG	1768
Tarsipes rostratus	GGCTCAAGTCTGGAATGAGAAGCGGCGGAGCGCGCGGGAG	1738
Vombatus ursinus	GGCTCAATTCTGGAATGAGAAGCGGCGGAGCGCGCGAGAG	1799

Species	1810	
Petaurus breviceps	GAATCTGTGCTCTCCTAC	1777
Acrobates pygmaeus	GAATCTGTGCTCTCCTAC	1809
Distoechurus pennatus	GAATCTGTGCTCTCCTAC	1795
Dactylopsila trivirgata	GAATCTGTGCTCTCCTAC	1816
Macropus giganteus	GAATCTGTGCTCTCCTAC	1816
Pseudochirops archeri	GAATCTGTACTCTCCTAC	1818
Phascolarctos cinereus	GAATCTGTGCTCTCCTAC	1796
Pseudochirops corinnae	GAATCTGTACTCTCCTAC	1818
Pseudochirops cupreus	GAATCTGTACTCTCCTAC	1814
Phalanger gymnotis	GAATTTGTGCTCTCCTAC	1798
Pseudocheirus occidentalis	GAATCTGTGCTCTCCTAC	1815
Pseudocheirus peregrinus	GAATCTGTGCTCTCCTAC	1801
Petauroides volans	GAATCTGTGCTCTCCTAC	1786
Tarsipes rostratus	GAATCTGTGCTCTCCTAC	1756
Vombatus ursinus	GAATCTGTGCTCTCCTAC	1817

GAR 13585

	10 20 30 40	
Petaurus breviceps	ACTCTACGCGGGTTTCCAGAAAGAGGCACGCGGATTCTAT	40
Acrobates pygmaeus	ACTCTATGCGGGTTTCCTGGAGGAGGCTCGCGGGTTCTAT	40
Distoechurus pennatus	ACTCTATGCGGGTTTCTTGGAAGAGGCACGCGGATTCTAT	40
Dactylopsila trivirgata	ACTCTATGCGGGTTTCCAGAAAGAGGCATGCAGATTCTAT	40
Macropus giganteus	ACTCTATGCGGGTTTCCAGGAAGAGGCACGCAGATTTTAT	40
Pseudochirops archeri	ACTCTATGCGGGTTTCCAGAAAGAGGCACGAGGATAAATA	40
Phascolarctos cinereus	ACTCTATGCGGGTTTCCAGGAAGAAGCACGCGGATTCTAT	40
Pseudochirops corinnae	ACTCTATGCGGGTTTCCAGAAAGAGGCACGAGGATTCTAT	40
Pseudochirops cupreus	ACTCTATGCGGGTTTCCAGAAAGAGGCACGAGGATTCTAT	40
Phalanger gymnotis	ACTCTATGCGGGTTTCCAGGAAGAGGCACGCGGATTCTAT	40
Pseudocheirus occidentalis	ACTCTATGCGGGTTTCCAGAAAGAGGCACGCGGATTCTAT	40
Pseudocheirus peregrinus	ACTCTATGCGGGTTTCCAGAAAGAGGCACGCGGATTCTAT	40
Petauroides volans	ACTCTATGCGGGTTTCCAGAAAGAGGCGCGCGGATTCTAT	40
Vombatus ursinus	CCTCTATGCGGGTTTCCAGGAAAAAGCACGCGAATGTTAT	40

	50 60 70 80	
Petaurus breviceps	ATCCA----------------ACATTTTCGAACTGAGAGA	64
Acrobates pygmaeus	TTCCACTTAATAGGCGCTCACACTTTTTCGAACTGAGAGA	80
Distoechurus pennatus	TTCCACATATTACGCGCACACACATTTCCGAACTGAGAGA	80
Dactylopsila trivirgata	TTCCACATACTATGCGCGCACACATTTTCGAACTGAGAGA	80
Macropus giganteus	TTCCACAGATTACGTGCATACACCTTTTCGAACTGGGAGA	80
Pseudochirops archeri	GACCACATATCATGCGCGCACACATTTTCGAACTGAGAGA	80
Phascolarctos cinereus	TTCCACATATTACTTGTATACACCTTTTCGAACTGAGAGA	80
Pseudochirops corinnae	TTCCACATATCATGCGTGCACACATTTTCGAACTGAGAGA	80
Pseudochirops cupreus	TTCCACATATCATGCGCACACATATTTTCGAACTGAGAGA	80
Phalanger gymnotis	TTCCACTTATTACTTGCGTACACCT-------GAGAGAAG	73
Pseudocheirus occidentalis	TTCCACGTATCATGCGCGCACACATTTTCGAACTGAGAGA	80
Pseudocheirus peregrinus	TTCCACGTATCATGCGCGCACACATTTTCGAACTGAGAGA	80
Petauroides volans	TTCCACATATCATGCGCGCACACATTTTCGAACTGAGAGA	80
Vombatus ursinus	TTCCACATATTACGTGTATACACCTTTTCGAACTGAGAGA	80

	90 100 110 120	
Petaurus breviceps	AGAGAGCTTCGGGAGAC-GGGAGAGTTGCAGGCAATGAGA	103
Acrobates pygmaeus	AGAGAGCTTCGGCAGACTGGGAGAATTGCAGGTGATGAGA	120
Distoechurus pennatus	AGAGAGCTTCGGCAGACTGGGAGAACTGCGGGCGATGAGA	120
Dactylopsila trivirgata	AGAGAGCTTCGGGAGACCGGGAGGGCTGCAGGC-------	113
Macropus giganteus	AGAGAGATTTGGAAGACGGGGAGTGTTGCAGGGGATGAGA	120
Pseudochirops archeri	AGAGAGCTTCGGGATATAGGGAGAGTTGCAGGCGATGACA	120
Phascolarctos cinereus	GGAAAGCTTCGAGAGAC-GGGAGAGTTGCAGGCGATGATA	119
Pseudochirops corinnae	AGAGAGCTTCGGAACATAGGGAGAGTTGCAGGCGATGATA	120
Pseudochirops cupreus	AGAGAGCTTCGGGATATAGGGAGAGTTGCAGGCGATGCTA	120
Phalanger gymnotis	AGAGAGCTTCGGAAGATGGGGAGAGTTGCAGGCGATGAGA	113
Pseudocheirus occidentalis	AGAGAACTTCGGCATACCGGGAGAGTTGCAGGCAATGACA	120
Pseudocheirus peregrinus	AGAGAACTTCGGCATACCGGGAGAGTTGCAGGCAATGACA	120
Petauroides volans	AGAGCGCTTCGCACGACCGGGAGAGTTGCAGGCGATGACA	120
Vombatus ursinus	AGACAGCTTCGAGAGACGGGGAGAGTTGCAGGCAATGAGA	120

130 140 150 160

Petaurus breviceps	TGGAAGAGGGGTTACGGAATGAGGTTCAGGGAGAAACTGC	143
Acrobates pygmaeus	TGGAAGAGGAGCTACGGAGTGAAGTTCAGGGAGAAACTCC	160
Distoechurus pennatus	TGGAAGGGGGGCTACGGAGTGGAGTTCAGGAAGAAACTCC	160
Dactylopsila trivirgata	------------------GTGAGGTTCAGGGAGAAACTGC	135
Macropus giganteus	TGGAAGAGAGACTACGGAGTGAGATTCGGAGAGAAACTG-	159
Pseudochirops archeri	AGGAAGAGGGGCTACGGAGCGAGGTTCAGGGAGAAACTGC	160
Phascolarctos cinereus	TGGAAGAGGAGGTACGGAGTGAGGTTCAGGGAGAAACTGC	159
Pseudochirops corinnae	TGGAAGAGGGGCTACGGAGCGAGGTTCAGGGAGAAACTGC	160
Pseudochirops cupreus	TGGAAGAGGGGCTACGGAGCGAGGTTCAGGGAGAAACTGC	160
Phalanger gymnotis	TGGAAGAGGGGCAACAGAGTGAGGTTCAGGGAGAAACCAC	153
Pseudocheirus occidentalis	TGGAAGAGGGGCTACGGAGCGAGGTTCAGGGAGAAACTGC	160
Pseudocheirus peregrinus	TGGAAGAGGGGCTACGGAGCGAGGTTCAGGGAGAAACTGC	160
Petauroides volans	TGGAAGAGGGGCTACGCAGCGAGGTTCAGGGAGAAACTGC	160
Vombatus ursinus	TGGAAGAGGGGGTACGGAGTGAGGTTCAGGGAGAAACTGC	160

170 180 190 200

Petaurus breviceps	CCCTTGGCTAGCCTCCCTCTCCTCCCCTCAAGAAATACGT	183
Acrobates pygmaeus	CCCTTGGCTAGTCCGCCTCCCCTCCCCTCAAGGAATACGT	200
Distoechurus pennatus	CCTTTGGCTAGTCCTCCTCCCCTCCCCTCAAGGAATACGT	200
Dactylopsila trivirgata	CCCTTGGGTAGCCCCCCTCCTCTCCCCTCAAGAAATACGT	175
Macropus giganteus	----------------TTCTCTCCCCGCCAAGGAATACAT	183
Pseudochirops archeri	CCCTTGCCTAGCCCTCCTCCCCTCCCCTCAAGGAATACGT	200
Phascolarctos cinereus	CCCTTGGCTAGTC-----CCCCTCCCCCTAAGGAATATGT	194
Pseudochirops corinnae	CCCTTGCCTAGCCCTCCTCCCCTCCCCTCAAGGAATACGT	200
Pseudochirops cupreus	CCCTTGCCTAGCCCTCCTCCCCTCCCCTCAAGGAATACGT	200
Phalanger gymnotis	CCCTCGGCTAGTCCCCCTTCCCTCCCCCCAAGGAATACGT	193
Pseudocheirus occidentalis	CCCTTGCCTAGCCCCCCTCCCCTCCCTTCAAGGAATACAT	200
Pseudocheirus peregrinus	CCCTTGCCTAGCCCCCCTCCCCTCCCTTCAAGGAATACGT	200
Petauroides volans	CCCTTGCCTAGCCCTCCTCCCCTCCCCTCAAGGAATACGT	200
Vombatus ursinus	CCCTTGGCTAGTC-----CCCCTCCCACTAAGAAATACGT	195

210 220 230 240

Petaurus breviceps	TCTTGCCTGGGAGAAAAAGGGAACCGGGGACACCAATATC	223
Acrobates pygmaeus	TCTTGCCTGCGAGAAGAAGGGAACCTATGACACCCATATC	240
Distoechurus pennatus	TCTTGCCTGGGAGAAGAAGGGAACCTCAAATACCAATATC	240
Dactylopsila trivirgata	TCTTGCCTGGGAGAAAAAGGGAACCTGGGACACCAATATC	215
Macropus giganteus	TCCTGCCTGGGAGAAGAATGGAACCTGGGACACCAGTATC	223
Pseudochirops archeri	TCTTGCCTGGGAGAAGAAGGGAACCTGGGACACCAAGATC	240
Phascolarctos cinereus	TCTTGCCTGGGAAAAGAATGGAACCTGGGACACCGTTATC	234
Pseudochirops corinnae	TCTTGCCTGGGAGAAGAAGGGAACCTGGG--ACCAAGATC	238
Pseudochirops cupreus	TCTTGCCTGGGAGAAGAAGGGAACCTGGGACACCAAGATC	240
Phalanger gymnotis	TCTTGCCTGAGAGAAGAAGGGAATCTGGGACGCCAATATC	233
Pseudocheirus occidentalis	TCTTCCCTGGGAGAAGAAGGGAACATGGGACACCAATATC	240
Pseudocheirus peregrinus	TCTTCCCTGGGAGAAGAAGGGAACATGGGACACCAATATC	240
Petauroides volans	TCTTGCCTGGGAGAAGAAGGGAACCTGGGACACCAATATC	240
Vombatus ursinus	TCTTGCCTGGGAGAAGAATGGAACCTGGGACACCGATATC	235

	250 260 270 280	
Petaurus breviceps	TCCTTTTCCCTCCTCCGAGCT-------ACTTCACTACTT	256
Acrobates pygmaeus	TCCTTTTCCCGCCTCCGATCTAATACTAACTTTACTACTT	280
Distoechurus pennatus	TCCTTTTCTCGCCTCCGATCTAAAACTAACT--ACTACTT	278
Dactylopsila trivirgata	TCCTTTTCCCGCCTCCGAGCTAATACTAACTTCACTACTT	255
Macropus giganteus	TCCCTTTCCTGCTTCCGATCTAATACTGAATTCACTA---	260
Pseudochirops archeri	TCCTTTTCCCGCCTCCGAGCTAATACTAACTTCACTACTT	280
Phascolarctos cinereus	TCCTTTCCCCGCCTCAGATGTAATACTAACTTCACTACTT	274
Pseudochirops corinnae	CCCTTTTTCCGCCTCCCAGCTAATACTAACTTCACTACTT	278
Pseudochirops cupreus	TCCTTTTTCCGCCTCCGAGCTAATACTAACTTCACTACTT	280
Phalanger gymnotis	TCCTTTCCCCACCTCCGATCTAATACTAA-TTCATTACTT	272
Pseudocheirus occidentalis	TCCTTTTCCCGCCTCCGAGCTAATACTAACTTCACTACTT	280
Pseudocheirus peregrinus	TCCTTTTCCCGCCTCCGAGCTAATACTAACTTCACTACTT	280
Petauroides volans	TCCTTTTCCCGCCTCCGAGTTAATACTAACTTCACTATTT	280
Vombatus ursinus	TCCTTTCCCCGCCTCAGATGTAATAGTAACTTCACTATTT	275

	290 300 310 320	
Petaurus breviceps	TGTCTCGGTAACAGTTGGACAAAACGTACTGTAATGCAGA	296
Acrobates pygmaeus	TGTCTCGGTAATAGTTGGACGAAAAGGCACAGGCAGAAGA	320
Distoechurus pennatus	TGTCTCGGTAATAGTTGGACAAAATGGCACAGACAGAAGA	318
Dactylopsila trivirgata	TGTCTCGGTAATAGTTGGATAAAACGGCACAGACTGAAGA	295
Macropus giganteus	TGTCTTGGTAATAGTTGGACAAAACGGCGCAGACAGCAGA	300
Pseudochirops archeri	TGTCTTGGTAATAGTTGAACAAAAAGGCACAGACGGCAGA	320
Phascolarctos cinereus	GTTCTCG------GTTGGAGAAAACGGCGCAGGTAGTAGA	308
Pseudochirops corinnae	TGTCTTGGTAATAGTTGAACAAAAAGGCACAGACGGCAGA	318
Pseudochirops cupreus	TGTCTTGGTAATAGTTGAACAAAAAGGCACAGACGGCAGA	320
Phalanger gymnotis	TGTTTCGGTAATAGCTGGACACAACGGCGCAGACAACAGA	312
Pseudocheirus occidentalis	TGTCTTGGTAGTAGTTGGACAAAAAGGCACAGACGGCCGA	320
Pseudocheirus peregrinus	TGTCTTGGTAATAGTTGGACAAAAAGGCACAGACGGCCGA	320
Petauroides volans	TGTCTTGGTAATAGTTGGACAAAAAGGCACAGACGGCAGA	320
Vombatus ursinus	GTTCTCGGTAATAGTTGGACAAAACGGCGCAGGTAGCAGA	315

	330 340 350 360	
Petaurus breviceps	GAACTTTAGTTATCCGAAACTGGGATGCCACGTTCCTGCG	336
Acrobates pygmaeus	GAACTTTAGTTCTCCGAAGCTAGGATGCCAAGTTCCTGCG	360
Distoechurus pennatus	GAACTTGAGTTCTCCGAAACTAGGATGCCAAGTTCCTGCG	358
Dactylopsila trivirgata	GAACTTTAATTATCCGAAACTGGAATGCCACGTTCCTGCG	335
Macropus giganteus	GAACTTTAGTTATCCGAAACTGGAATGCCACGTTCCTGCG	340
Pseudochirops archeri	GAACTTTAGTTATCCGAAACTGGGATGCCACGTTCCTGCG	360
Phascolarctos cinereus	GAACTTTAGTTATCCGAAACTGGGCTGCCACGTTCGTGCC	348
Pseudochirops corinnae	GAACTTTAGTTATCCGAAACTGGGATGCCACGTTCCTGCG	358
Pseudochirops cupreus	GAACTTTAGTTATCCGAAACTGGGATGCCACGTTCCTGCG	360
Phalanger gymnotis	CAACTTTAGTTATCCGAAACTGGGATGCCACGTTCCTGCG	352
Pseudocheirus occidentalis	GAACTTTAGTTATCCGAAACTGGGATGCCACGTTCCGCGG	360
Pseudocheirus peregrinus	GAACTTTAGTTATCCGAAACTGGGATGCCACGTTCCGCGG	360
Petauroides volans	AAACTTTAGTTATCCGAAACTGGGATGCCACGTTCCTGCG	360
Vombatus ursinus	GAACTTTAGTTATCCGAAACTGGGCTGCCACGTTCCTGCG	355

```
                                     370       380       390       400
Petaurus breviceps            TTCTTGAGCCCTTTGCTGCCCCTGTGGCTCACGCACAGGA 376
Acrobates pygmaeus            TTCTTGAGCCCTTTGCAGCCCCTGGGGCTCATGCATAGGG 400
Distoechurus pennatus         TTCTTGAGCTCTTTGCAGCCCC-GGGGCTCATGCACAGGG 397
Dactylopsila trivirgata       TTCTTGAGCCCTTTGCTGCCCCTGTGGCTCACGCACAGGG 375
Macropus giganteus            TTCCTGCGCCTTTCGCTGCCCCTGGGGCTCGCACTCAGAG 380
Pseudochirops archeri         TTCTTGAGCCCTTTGCTGCCCCTGTGGCTCACGCAAAGGG 400
Phascolarctos cinereus        TTCTTGCGCCTTT--CTGCCCCTGTGGTTCGCGCACTGGG 386
Pseudochirops corinnae        TTCTTGAGCCCTTTGCTGCCCCTGTGGCTCATGCAAAGGG 398
Pseudochirops cupreus         TTCTTGAGCCCTTTGCTGCCCCTGTGGCTCACGCAAAGGG 400
Phalanger gymnotis            TTCCTGAGCCCTTTGCTGCCCCTGTGGCTCGTGCACAGGG 392
Pseudocheirus occidentalis    TTCTTGAGCCCTTTGCTGCCCCTGTGGCTCACGCACAGGG 400
Pseudocheirus peregrinus      TTCTTGAGCCCTTTGCTGCCCCTGTGGCTCACGCACAGGG 400
Petauroides volans            TTCTTGAGCCCTTGGCTGCCCCTGTGGCTCACGCACAGGG 400
Vombatus ursinus              TTCTTGCGCCTTTTG-----CCCGTGGCTCGCGCAATGGG 390
```

```
                                     410       420       430       440
Petaurus breviceps            ACATGACCCGTCCTCTGGTCTTTGGCCGCTCCTCAACTAT 416
Acrobates pygmaeus            ACATGACGGTTCCTCTGGTCTTTGGCCGCTCCTCAACTAT 440
Distoechurus pennatus         ACTTGACCGGTCGTCTGGTCTTTGGCCGCTCCTCAACTCT 437
Dactylopsila trivirgata       ACATGACCCGTCCTCTGGTCTTTGGCCGCTCCTCAACTAT 415
Macropus giganteus            ACTTGACCGGTCCTCTGGTCTTTGACCGCTCCTCAACTAT 420
Pseudochirops archeri         ACATGACCGGTCCTCTGGTCTTTGGCCGCTCCTCAACTAT 440
Phascolarctos cinereus        ACATGACCGGTCCTCTGGTCTTTGGCCGCTCTTCAACTAC 426
Pseudochirops corinnae        ACACGACCGGTCCTCTGGTCTTTGGCCG---CTCAACTAT 435
Pseudochirops cupreus         ACATGACCGGTCCTCTGGTCTTTGGCCGCTCCTCAACTAT 440
Phalanger gymnotis            ACATGACCGATCCTCTGGTCTTTGGCCGCTCCTCAACTAC 432
Pseudocheirus occidentalis    ACATGACGGGTCCTCTGGTCTTTGGCCGCTCCTCAACTAT 440
Pseudocheirus peregrinus      ACATGACGGGTCCTCTGGTCTTTGGCCGCTCCTCAACTAT 440
Petauroides volans            ACATGACCAGTCCTCTGGTCTTTGGCCGCTCCTCAACTAT 440
Vombatus ursinus              ACATGACCGGCCCTCTGGTCTTTGGCCGTTCCTCAACTAT 430
```

```
                                     450       460       470       480
Petaurus breviceps            TGACCGGGAGGCGTGGCCGTTGCGCCAGAGAAGCGACTGG 456
Acrobates pygmaeus            TGACCGGGAGGCGTGGCCGTTGAGCCCGAGAAGTGACTGG 480
Distoechurus pennatus         CGACCGGGAGGCGTGGCCGTTGCGCCGGAGAAGTGACTGG 477
Dactylopsila trivirgata       TGACCGGGAGGCGTGGCCGTTGCGCCAGAGAAGTGACTGG 455
Macropus giganteus            TGGCCTGGAGGCGTGGCCCTTGTGGCCGAGAAGTAACTGC 460
Pseudochirops archeri         TGACCGGGAGGCGTGGCCGTTGCGCCAGAGAAGTGACTGG 480
Phascolarctos cinereus        TGGCCTGGAGGCGTGGCCGGTGCAGCCGAGAAGTGACTGG 466
Pseudochirops corinnae        TGACCGGGAGGCGTGGCCGTTGCGCCAGAGAAGTGACTGG 475
Pseudochirops cupreus         TGACCGGGAGGCGTGGCCGTTGCGCCAGAGAAGTGACTGG 480
Phalanger gymnotis            TGGCCTGGAGGCGTGGCGGTTGCGGCCGAGAAGTGACTGG 472
Pseudocheirus occidentalis    TGACCGGGAGGCGTGGCCGTTGCGCCAGAGAAGTGACTGG 480
Pseudocheirus peregrinus      TGACCGGGAGGCGTGGCCGTTGCGCCAGAGAAGTGACTGG 480
Petauroides volans            TGACCGGGAGGCGTGGCCGTTGCGCCAGAGAAGTGACTGG 480
Vombatus ursinus              TGGCCTGGAGGCGTGGCTGGTGCAGCAGAGAAGTGACTGG 470
```

	490 · 500 · 510 · 520	
Petaurus breviceps	CTCAGGGAGTGCGCCTTGTTCTCTATCTCAAAAGAGGAGG	496
Acrobates pygmaeus	CTCCGAGAGTGAGCCTTGTTCTCTATCTCAAAAGAGGAGG	520
Distoechurus pennatus	CTCCGGGAGTGAGCCTTGTTCTCTCTCTCAAAAGAGGAGG	517
Dactylopsila trivirgata	CTCAGGGAGTGAGCCTTGTTCTCTATCTCAAAAGAGGAGG	495
Macropus giganteus	CTCCGGGAGTGAGTCTTGTTCTCTAACTCAAAAGAGGAGG	500
Pseudochirops archeri	CTCGGGGAGTGAGCCTTGTTCTCTATCTCAAAAGAGGAGG	520
Phascolarctos cinereus	C---GGGAGTGAGCCTTGTTCTCTATCTCAAAAGAGGAGG	503
Pseudochirops corinnae	CTCGGGGAGTGAGCCTTGTTCTCTATCTCAAAAGAGGAGG	515
Pseudochirops cupreus	CTCGGGGAATGAGCCTTGTTCTCTATCTCAAAAGAGGAGG	520
Phalanger gymnotis	CTCCGGGAGTGAGCCTTGTTCTCTATCTCAAAAGAGGAGG	512
Pseudocheirus occidentalis	CTCGGGGAGTGAGCC-TGTTCTCTATCTCAAAAGAGGAGG	519
Pseudocheirus peregrinus	CTCGGGGAGTGAGCC-TGTTCTCTATCTCAAAAGAGGAGG	519
Petauroides volans	CTCGGGGAGTGAGCCTTGTTCTCTGTCTCAAAAGAGGAGG	520
Vombatus ursinus	C---GGGAGTGAGTCTTGTTCTCTATCTCAAAAGAGGAGG	507

	530 · 540 · 550 · 560	
Petaurus breviceps	GGAAAACGAAGGATAATTTGTTTAAAGTGACCTTATGTCT	536
Acrobates pygmaeus	GGAAGAGGAAGGACAATTTGTTTAAAGTGACTTTCAGTCT	560
Distoechurus pennatus	GGAAAAGGAAGGATAATTTGTTTAAAGTGACCTTATGTCT	557
Dactylopsila trivirgata	GGTAAACCAAGGATAATTTGTTTAAAGTGACCTTATGTCT	535
Macropus giganteus	AGAAAACGAAGAATAATTTGTTTAAAGTGACCTTATGTCT	540
Pseudochirops archeri	GGAAAACGAAGGATAATTTGTTTAAAGTGACCTTATGTCT	560
Phascolarctos cinereus	GGAAAACGAAGGATAATTTGTTTAAAGTGACCTTATGTCT	543
Pseudochirops corinnae	GGAAAACAAAGGATAATTTGTTTAAAGTGACCTTATGTCT	555
Pseudochirops cupreus	GGAAAACGAAGGATAATTTGTTTAAAGTGACCTTATGTCT	560
Phalanger gymnotis	GGAAAACGAAGGATAACTTGTTTACAGTGACCTTATGGTT	552
Pseudocheirus occidentalis	AGAAAACGAAGGATAATTTGTTTAAAGTGACCTTATGTCT	559
Pseudocheirus peregrinus	AGAAAACGAAGGATAATTTGTTTAAAGTGACCTTATGTCT	559
Petauroides volans	GGAAAACGAAGGATAATTTGTTTAAAGTGACCTTATGTCT	560
Vombatus ursinus	GGAAAACGAAGGATAATTTGTTTAAAGTGACCTTATGTCT	547

	570 · 580 · 590 · 600	
Petaurus breviceps	ATTATTCAAGCACATCAGCTTCCCGGGTTTTTGTGCAGAT	576
Acrobates pygmaeus	GTTACTGAAGTACACCAGCTTCTCGGGTTTTTGTGCAGAT	600
Distoechurus pennatus	GTTATTGAAGTACACCAGCTTCTCGGGTTTTTGTGCAGAT	597
Dactylopsila trivirgata	ATTATCCAAGCACATCAGCTTCCCGGGTTTTTGTGCAGAT	575
Macropus giganteus	ATTATACAAGTACACTAGCTTCCCAGGCTTTTGTGCGGAT	580
Pseudochirops archeri	ATTATTCAAGTACACCAGCTTCCCGGGTTTTTGTGCAGAT	600
Phascolarctos cinereus	GTTATGCAAGTACACCAGCTTCCCGGTTTTTTGTGCAGAT	583
Pseudochirops corinnae	ATTATTCAAGTACACCAGCTTCCCGGGTTTTTGTGCAGAT	595
Pseudochirops cupreus	ATTATTCAAGTACACCAGCTTCCCGGGTTTTTGTGCAGAT	600
Phalanger gymnotis	ATTACGCAAGTACACCAGCTTCCCGGGTTTTTGTGCAAAT	592
Pseudocheirus occidentalis	ATTATTCAAGTACACCAGCTTTCCGGGTTTTTGTGCAGAT	599
Pseudocheirus peregrinus	ATTATTCAAGTACACCAGCTTTCCGGGTTTTTGTGCAGAT	599
Petauroides volans	ATTATTCAAGTACACCAGCTTCCCGGGTTTTTGTGCAGAT	600
Vombatus ursinus	ATTATGCAAGTACACCAGCTTCCCGGATTTTTGTGCAGAT	587

```
                                     610       620       630       640
Petaurus breviceps           CACAGAAAATCCACCCCGCTGGAGCTGACTGAGTTAGCCC  616
Acrobates pygmaeus           CACAGAAAATCCATCCCGCCGGAGCTGACTGATTTAGCTC  640
Distoechurus pennatus        CACAGAAAATCCACCCCGCCGGAG----CTGATTTAGCTC  633
Dactylopsila trivirgata      CACAGACAATCTACCCCGCTGGAGCTGACTGAGTTAGCTC  615
Macropus giganteus           GACAGAAAATCTACCACGCCGGAGCTGACTGATTTAGCTC  620
Pseudochirops archeri        CACAGAAAATCCACCCCGCTGGAGCTGACTG----AGCCC  636
Phascolarctos cinereus       CACAGAACATCCACCACGCCGGAGCTGACTGATTTAGCTC  623
Pseudochirops corinnae       CACAGAAAATCCACACCGCTGGAGCTGACTGAGTTAGCCC  635
Pseudochirops cupreus        CACAGAAAATCCACACCGCTGGAGCTGACTGAGTTAGCCC  640
Phalanger gymnotis           CACAGAAAATCCATCACGTTGGAGCTGACTGATTTAGCTC  632
Pseudocheirus occidentalis   CACAGAAAATCCACCCCGCTGGAACTGACTGAGTTAGCCT  639
Pseudocheirus peregrinus     CACAGAAAATCCACCCCGCTGGAACTGACTGAGTTAGCCC  639
Petauroides volans           CACAGAAAATCCACCCCGCTGGAACTGACTGAGTTAGCCC  640
Vombatus ursinus             CACAGAAAGTCCACCACGCCAGAGCTGACTGATTTAGCTC  627
```

```
                                     650       660       670       680
Petaurus breviceps           AGAAATCTGTCCACACACACCGTTGCAGGGTGGGGTTTGA  656
Acrobates pygmaeus           TGAAATCTGTCCGCACACACAGTCCCAGGGTGGGGTTTGA  680
Distoechurus pennatus        TGAAATCTGTCGGCACACACAGTCCCAGGGTGGGGTTTGA  673
Dactylopsila trivirgata      AGAAATCTGTCCACACACACCGTCGCAGGGTGGGATTTGA  655
Macropus giganteus           TGAAATCTGTCGGCACACACCGTCGCAGGGTGGGGTTTAA  660
Pseudochirops archeri        CGAAATCTGTCTG------------CAGAGTGGGGTTTGA  664
Phascolarctos cinereus       TGAAATCTGTCCACACACACCGTCGCAGGGTGGGGTTTGA  663
Pseudochirops corinnae       CGAAATCTGTCTG------------CAGAGTGGGGTTTGA  663
Pseudochirops cupreus        CGAAATCTGTCTG------------CAGAGTGGGG-TTGA  667
Phalanger gymnotis           TGAAATCTGTTCGCACACACCGTCGCAAGGTGGGGTTTGA  672
Pseudocheirus occidentalis   CGAAATCTGCCTGCACACACCGTTGCAGGGTGGAGTTTGA  679
Pseudocheirus peregrinus     CGAAATCTGCCTGCACACACCGTTGCAGGGTGGGGTTTGA  679
Petauroides volans           CGAAATCTGTCTGCACACACCGTTTCAGGGTGGGGTTTGA  680
Vombatus ursinus             TTAAATCTGTCCGTACACACCATCGCAGGGTGGGGTTTGA  667
```

```
                                     690       700       710       720
Petaurus breviceps           ACTCTTGGCAGCAAGTATAAAGAACTGGGAGTCGGTGGGT  696
Acrobates pygmaeus           ACTCTTGGCAGCAAGTATAAAGAACTGGGGGTCGGTGGGT  720
Distoechurus pennatus        ATTCTTGGCAGCAAGTGTAAAGAACTGGGGGTCGGTGGGT  713
Dactylopsila trivirgata      ACTTTTGGCAGCAAGTATAAAGAACTGGGAGTCGTTGGGT  695
Macropus giganteus           ACGCTTGGGACCAAGTATAAAGAACTGGGGGTCGGTGGGT  700
Pseudochirops archeri        ACTCTTGGCAGCAAGTATAAAGAACTGGGGGTCGGTGGGT  704
Phascolarctos cinereus       ACTCTTGGCAGAAAGTATAAAGAACTGGGATTAGGTGGGT  703
Pseudochirops corinnae       ACTCTTGGCAGCAAGTATAAAGAACTGGGAGTCGGTGGGT  703
Pseudochirops cupreus        ACTCTTGGCAGCAAGTATAAAGAACTGGGGATCGGTGGGT  707
Phalanger gymnotis           ACTCTTGGCAGCAAGTATAAAGAACTGGGGGTCGGTGGGT  712
Pseudocheirus occidentalis   ACTCTTGGCAGCAAGTATAAAAAACTGG--------GGGT  711
Pseudocheirus peregrinus     ACTCTTGGCAGCAAGTATAAAAAACTGG--------GGGT  711
Petauroides volans           ACTCTTGGCAGCAAGTATAAAGAACTGGGGGTCGGTGGGG  720
Vombatus ursinus             ACTCTTGGCAGAAAGCATAAAGAACTGGGAGTCGGTGGGT  707
```

	730 740 750 760	
Petaurus breviceps	TAAGGGTGAGGAGGATGTGTACTTTTTTTTTTTGGGTCCT	736
Acrobates pygmaeus	TAAGGGTAAGGAGGAGGTGTACCTTTTGGGGGGGGGGTCA	760
Distoechurus pennatus	TAAGGGTAAGGAGGAGGTGTACCTTGGGGGGGGGGGTCAT	753
Dactylopsila trivirgata	TAAGGGTGGGGAGGATGTGTACCTTTGGGGGGGGGGTCCT	735
Macropus giganteus	TAAGGGTAAGGAGGATGTGTATCTTTTTTTT----GTCGT	736
Pseudochirops archeri	TAAGGGTGAGAAGGCTGTGTATCTTTTTTTTTTTGGTCCT	744
Phascolarctos cinereus	TAAGGGTAAGGAGGA---GTGTATCTTTTTTTTTTGTCTT	740
Pseudochirops corinnae	T---------AAGGCTGTGTATC---TTTTTTTTGGTTCT	731
Pseudochirops cupreus	T---------AAGGCTGTGTATCTTTTTTTTTTTGGTCCT	738
Phalanger gymnotis	TAAGGGTGAGGAGGATGTGTCTTTTTTTTTTTTTGGTCGT	752
Pseudocheirus occidentalis	TAAGGGTGAGAAGGCTGTGTATCTTTTTTTTTTTGGTCCT	751
Pseudocheirus peregrinus	TAAGGGTGAGAAGGCTGTGTATCTTTTTTTTTTTGGTCCT	751
Petauroides volans	TAAGGGTGAGAAAGCTGTGTATCTTTTTTTTTTTTGTCCT	760
Vombatus ursinus	TAAGGGTAAGGAGGATGTGTATCTTTTTTTTTTTCGTCGT	747

	770 780 790 800	
Petaurus breviceps	TGAAAGAGGCTACTTCCCCTTCTCTCTCTCCTAATCCACC	776
Acrobates pygmaeus	TTGAGAAAGCTATCTTCCCTTCTCTCCCTCCTCCTACACC	800
Distoechurus pennatus	TGAGAGAGGCTACCTTTCCTTGTCTCCCTCCTCCCACACC	793
Dactylopsila trivirgata	TGAGAGAGGCTACTTCCCCTTCTCTCTCTCCTCATCCACC	775
Macropus giganteus	TGAGAGAGGCTACCTCCCCTTCTTTCCCTCCTCCTCCACC	776
Pseudochirops archeri	TGAGAGAGGCTACTTCCCCTTCTCTCCCTCCTCATCCACC	784
Phascolarctos cinereus	TGGGAGAGACTACCTCCCCTTCTCTCCCTCCTCCTCCACC	780
Pseudochirops corinnae	TGAGAGAGGCTACTTCCCCTTCTCTCCCTCCTCATCCACC	771
Pseudochirops cupreus	TGAGAGAGGCTACTTCCCCTTCTCTCCCTCCTCATCCACC	778
Phalanger gymnotis	TGAGAGAGGCTGCCTCTCCTTCTCGGCCTCCTCATCCATC	792
Pseudocheirus occidentalis	TGAGAGAGGCTACTTCCCCTTCTCTCCCTCCTCATCCACC	791
Pseudocheirus peregrinus	TGAGAGAGGCTACTTCCCCTTCTCTCCCTCCTCATCCACC	791
Petauroides volans	TGAGAGAGGCTACTTCCCCTTCTCTCCCTCCTCATCCATC	800
Vombatus ursinus	TGGGAGAGGCTACCTCCCCTTCTCTCCCTCCTCCTCCACC	787

	810 820 830 840	
Petaurus breviceps	CACCCTTGTTCAATCTCTGGGCATCTGGGCTCTAGAGGGG	816
Acrobates pygmaeus	CACCCTTGTTCAATCTCTGGGCATCTGGGCTCTAGAGGGG	840
Distoechurus pennatus	CACCCTTGTTCAATCTCTGGTCCTCTGGGCTCTAGAGGGG	833
Dactylopsila trivirgata	CACCCTTGTTCAATCTCTGGGCATCTGGGCTCTAGAGGGG	815
Macropus giganteus	CACCC-TGTTCAATCATTGGGCATCTGGGTTCTAGAAGGG	815
Pseudochirops archeri	CACCCTTGTTCAATCTCTGGGCACCTGGGCTCTAGAGGGA	824
Phascolarctos cinereus	CACCCTTGTTCAATCGCTGGGCATCTGGACTCTAGAGGGG	820
Pseudochirops corinnae	CACCCTTGTTCAATCTCTGGGCATCTGGGCTCTAGAGGGA	811
Pseudochirops cupreus	CACCCTTGTTCAATCTGTGGGCATCTGGGCTCTAGAGGGA	818
Phalanger gymnotis	CACCGTTGTTCGGTCGCTGGGTATCTGGGCTCTAGAGGGA	832
Pseudocheirus occidentalis	CACCCTTGCTCAGTGTCTGGACATCTGTGCTCTAGAGGGG	831
Pseudocheirus peregrinus	CACCCTTGCTCAATGTCTGGACATCTGTGCTCTAGAGGGG	831
Petauroides volans	CACCCTTGTTCAATCTCTGGGCATCTGGGTTCTAGAGGGG	840
Vombatus ursinus	CACCCTTGTTCAATCGCTGGGCATCTGGGCTCTAGAGGGG	827

	850 860 870 880	
Petaurus breviceps	GTAGGAGGAGGTGTATGGAAGAATAAAAGTCAGCTATTTC	856
Acrobates pygmaeus	GTAGGAGGAGGTGTATGGAAGGGAAAAAGTCAGCTGTTTC	880
Distoechurus pennatus	GTAAGAGGAGGTGTATGGAAGGATAAAAGTCAGCTGTTTC	873
Dactylopsila trivirgata	GTAGGAGAAGGTGTATGGAAGAATAAAAGTCAACTATTTC	855
Macropus giganteus	GTAGGAGAAGGTGTATAGAAGGATAAAAGTCAGCTGTTTC	855
Pseudochirops archeri	GTAGCAGGAGGTGTATGGAAGGATTAAAGTCAGCTATTTC	864
Phascolarctos cinereus	GTGGGGGGAGGTGTATGCAA--------------------	840
Pseudochirops corinnae	GTAGGAGGAGGTGTATGGAAGGATAAAAGTCAGCTATTTC	851
Pseudochirops cupreus	GTAGGAGGAGGTGTATGGAAGGATAAAAGTCAGCTATTTC	858
Phalanger gymnotis	GTAGGAGGTGGTATATGGAAGGATAAAAGTTACCTGTTTC	872
Pseudocheirus occidentalis	--AGGAGGAGGTGTATGGAAGGATAAAAGTCAGCTATTTA	869
Pseudocheirus peregrinus	--AGGAGGAGGTGTATGGAAGGATAAAAGTCAGCTATTTA	869
Petauroides volans	GTAGGAGAAGGTGTATGGAAGGATAAAAGTCAGCTATTTC	880
Vombatus ursinus	GTGGGGGGAGGTGTAAGGAAGGATAAAAGTCAGCTA-TTC	866

	890 900 910 920	
Petaurus breviceps	GTG---GCACTGCTTAGAACTCTCAGAAAAGCAGGTCGTT	893
Acrobates pygmaeus	GTGCACGAACTGCTTAGAACTCTCTGAAGAGCAGTTTGTT	920
Distoechurus pennatus	ACGGATGGACAGCTTAGAACTCTCAGAAGAGCACTTC-TT	912
Dactylopsila trivirgata	TTGGATGGACTGCTTAGAACTCTCAGAAAAGCAGGTCGTT	895
Macropus giganteus	CTGGACGGACTGCTTAGAACTCTCTCAAAAGCCGGTCTCT	895
Pseudochirops archeri	GTGAATGGACGGCTTAGAACTCTGAGAAAAGCAGGTCGTT	904
Phascolarctos cinereus	--GGATGGACTAGTTAGAACTCT--GAAGAACTGGTCGCT	876
Pseudochirops corinnae	GTGAATGGACTGCTTAGAACTCCGAGAAAAGCAGGTCGTT	891
Pseudochirops cupreus	GTGAATGGACTGCTTAGAACTCTGAGAAAAGCAGGTCGTT	898
Phalanger gymnotis	GTGGATGGTCTGCTTAGAACCCTCAGACGAGCAGGTCGCT	912
Pseudocheirus occidentalis	GTGAACGGACGGCTTAGAA--CTTAGAAAAGCAGGTCGTT	907
Pseudocheirus peregrinus	GTGAACGGACGGCTTAGAA--CTTAGAAAAGCAGGTCGTT	907
Petauroides volans	GTGAATGAACTGCTTAGAACTCTCAGAAAAGCAGGTCGTT	920
Vombatus ursinus	GTGGATGGACTGGTTAGAACTCT--GAAGAGCAGGTCGCT	904

	930 940 950 960	
Petaurus breviceps	CCAAACTCCCTTTGTGGTTTTGTGCCCGTGAGACCACTAG	933
Acrobates pygmaeus	CCAAACTTCCTTTGTGGTTTTGTGCCCGTGAAACCACGAG	960
Distoechurus pennatus	CCAAACTTTCTTTGTGGTTTTGTGACGGTGAGACCACGAG	952
Dactylopsila trivirgata	CCAAATTCCCTTTGTGGTTTTGTGCCCGTGGGACCACTAG	935
Macropus giganteus	CCAAACTTCCTTTGTAGTTCTGTGCCCATGAGACCACTAG	935
Pseudochirops archeri	CCAAACTCTCTTTGTGGTTTTGTGCCCGTGATACCACTAG	944
Phascolarctos cinereus	CCAAACTTCCTTTGTGGGTTTGTGCCCGTGAGACCACTAG	916
Pseudochirops corinnae	CCAAACTCTCTTTGTGGTTTTGTGCCCGTGATACCACTAG	931
Pseudochirops cupreus	CCAAACTCTCTTTGTGGTTTTGTGCCCGTGACACCACTAG	938
Phalanger gymnotis	CCAAACTTCC--------TTTGTGCCTGTGTGACCGCTAG	944
Pseudocheirus occidentalis	TCAAACT--CTTTGTGGTTTTGTGCTCGTGAGACCACTAG	945
Pseudocheirus peregrinus	TCAAACT--CTTTGTGGTTTTGTGCTCGTGAGACCACTAG	945
Petauroides volans	CCAAACTCTCTTTGTGGTTTTGTGTCTGTGAGACCACTAG	960
Vombatus ursinus	CCAAACTTCCTTTGTGGGTTTGTGCCCGTGAGACCACTAG	944

	970 980 990 1000	
Petaurus breviceps	GCGGCGCTGCTCACACACCAATCTGGCTCCTCTGTTAGTA	973
Acrobates pygmaeus	GCGGCGCTGCTCACACACCAATCTGGCTCCTCTCTTCGTA	1000
Distoechurus pennatus	GCGGCGCTGCTCACACACCAATCTGGTTCCTCTCTTAGTA	992
Dactylopsila trivirgata	GCGGCGCTGCTCACACACCAATCTGGCTCCTCTGTTAGTA	975
Macropus giganteus	GCGGCGCTGCTCACACGCCAATCTGGCTCCTCTGTTAGTA	975
Pseudochirops archeri	GCGGCGCTGCTCACACATCAATCTGGCTCCTCTATTAGTA	984
Phascolarctos cinereus	GGGGCGCTGCTCACACACCAATCTGGCTCTTCTGTTAGTA	956
Pseudochirops corinnae	GCGGCGCTGCTCACACACCAATCTGGCTCCTCTATTAGTA	971
Pseudochirops cupreus	GCGGCGCTGCTCACACACCAATCTGGCTCCTCTATTAGTA	978
Phalanger gymnotis	GCGGCGCTGCTCACACACCAATCTGGCTTCTCTGTCAGTA	984
Pseudocheirus occidentalis	GCGGCGCTGCTCACACACCAATCTGGCTCCTCTGTTAGTA	985
Pseudocheirus peregrinus	GCGGCGCTGCTCACACACCAATCTGGCTCCTCTGTTAGTA	985
Petauroides volans	GCGGCGCTGCTCACACACCAATCTGGCTCCTCTGTTAGTA	1000
Vombatus ursinus	GCGGCGCTGCTCACACACCAATCTGGCTCGTCTGTTAGTA	984

	1010 1020 1030 1040	
Petaurus breviceps	AGGTATTCCCGCAGAGTCCCCTCATTCTCAGCGAAGCGAG	1013
Acrobates pygmaeus	AGGTATTTCTACAGAATCGCCTCAGTCGCAGCGAAGCGAG	1040
Distoechurus pennatus	AGGTATTCCTGCAAAATCGCCTCACTCGCAGAGAAGCGAG	1032
Dactylopsila trivirgata	AGGTATTCCCGCAAAGTCCCCTCAGTCTCTGGGAAGCGAG	1015
Macropus giganteus	AGTTATTACCTCAGAGTCGCCTCAGTCCAAGAGAAGCGAG	1015
Pseudochirops archeri	AGGTTTTCCCGCAGAGTCACCTCAGTCGCAGCGAAGCGAG	1024
Phascolarctos cinereus	AGGTACTCCAGCACAGCCGCCTCAGTCGCAGAGAAGCGTA	996
Pseudochirops corinnae	AGGTTTTCCCGCAGAGTCGCCTCAGTCGCAAGGAAGCGAG	1011
Pseudochirops cupreus	AGGTTTTCCTGCAGAGTGGTCTCAGTCGCAGGGAAGCGAG	1018
Phalanger gymnotis	AGATATTCCCGCAGAGTCGCCTCAGCCGCAGAGAACCCAG	1024
Pseudocheirus occidentalis	AGGTTTTCCAGC--AGTCCCCTCAGTCGCCTCGAAGCGAG	1023
Pseudocheirus peregrinus	AGGTTTTCCAGC--AGTCCCCTCAGTCGCCTCGAAGCGAG	1023
Petauroides volans	AGGTTTTCCCTCAGAGTGGCCTCAGTCGCAGTGAAGCGAG	1040
Vombatus ursinus	AGGTATTCCAGCAGAGCAGCCTCAGTCGCAGAGAAGCGGG	1024

	1050 1060 1070 1080	
Petaurus breviceps	GTGGGGTGGGGGGGCATTCATTTATTTGTATACCACAAGA	1053
Acrobates pygmaeus	GTGAGGTGGGGGGGCGTTCATTTATTTGTGTGCCACAAAG	1080
Distoechurus pennatus	GTGAGGTGGGGGGGTATTCATTTATTTGTGTGCCACAAGG	1072
Dactylopsila trivirgata	GTGGGGTGGGGGGGTATTCATTTATTTGTATACTACAAGA	1055
Macropus giganteus	GTGGAGTGGGGGGGTATTCATTTATTTGTGTGCCACAAGG	1055
Pseudochirops archeri	GTGGGGTGGGGGGGCATTCATTTATTTGTATACCACAAGG	1064
Phascolarctos cinereus	ATGAGGCGGTAGGGCATTCATTTATTTGAGTGCCAACAGG	1036
Pseudochirops corinnae	GTGGGGTGGGGGGGCATTCATTTATTTGTATACCACAAAG	1051
Pseudochirops cupreus	GTGGGGTGGGGGAGCATTCATTTATTTGTATACCACAAGG	1058
Phalanger gymnotis	GTGGAGT-GGGGAGTATTCATTTATTTGTGTGCCACAAGG	1063
Pseudocheirus occidentalis	GTGGGGT-GGGGGGCATTCATTTATTTGTATACCAGAAAG	1062
Pseudocheirus peregrinus	GTGGGGT-GGGGGGCATTCATTTATTTGTATACCAGAAAG	1062
Petauroides volans	GTGGGGTGGAGGGGCATTAATATATTTGTATACCACAAGG	1080
Vombatus ursinus	ATGGAGTAGTGGGGCATTCGTTTATTTGCGTGCCATCAGG	1064

	1090 1100 1110 1120	
Petaurus breviceps	GGAAAAAATGTCCGGTTTTCTTGTACATACCGTCCCTCTC	1093
Acrobates pygmaeus	AAGAAAAAAATCCAGTTTTAGTATTCGTACCGTCCCTCTC	1120
Distoechurus pennatus	A--AAAAAAGTCCAGTTTTCGTATTCTTATCGTCCCTCTC	1110
Dactylopsila trivirgata	GAAAAAAATGTCCAGTTTT-GTGTTCGTACCGTCCCTCTC	1094
Macropus giganteus	AAAAAAAACTTCCAGTTTTCGTGTTCGTAGCGTCTCTCTC	1095
Pseudochirops archeri	GGGAAAAATGTCCAGTTTTCGTGTTCGTACCGTCCCTCTC	1104
Phascolarctos cinereus	A--AATAATTTCCCGTTTTCTTGCTTGTACCATCCCTCTC	1074
Pseudochirops corinnae	GGGAAAAATGTCCAGTTTTCTTGTTCGTACCGTCCCTCTC	1091
Pseudochirops cupreus	GGGGAAAATGTCCAGTTTTCGCGTTTGTACCGTCCCTCTC	1098
Phalanger gymnotis	AAATTTTTTTTCCAGTTTTCGTGTGCATACCATCCCTCTC	1103
Pseudocheirus occidentalis	GGGAAAAATGTCCAGTTTTCGTGTTCGTACGGCTCCCCTC	1102
Pseudocheirus peregrinus	GGGAAAAATGTTCAGTTTTCGTGTTCGTACGGCTCCCCTC	1102
Petauroides volans	GGGAAAAATGTCCAGTTTTCGTGTTCGTACAGTCCCTCTC	1120
Vombatus ursinus	AAAAATAATTTACAATTTTCGTGTTCGTACCGTTCCTCTC	1104

	1130 1140 1150 1160	
Petaurus breviceps	CATGTGTACTGAGAAACTAAGAAAGTAAGAAACAGTCTAG	1133
Acrobates pygmaeus	TATGTGTGCTGAGAAACTAAGAAAGCAGAAAACAATCTAA	1160
Distoechurus pennatus	TATGTGTGCTGAGAAACTAAGAAAGTAGGAAACAATCTAG	1150
Dactylopsila trivirgata	CATGTGTGCTGAGAAACTAAGAAAGTAGGAAACAGTATAG	1134
Macropus giganteus	CATGTGTGCTGAGAAACTAAAAAAGTGGGAAACAATCTAG	1135
Pseudochirops archeri	TATGTGTGCTGAGAAACTAAGAAAGTAGGAAGCAATCTAG	1144
Phascolarctos cinereus	TATGTGTGCTGCGAAACTAAGAAAGTAGGAAACAATCTAG	1114
Pseudochirops corinnae	TATGTGTGCTGAGAAACTAAGAAAGTAGGAAGCAATCTAG	1131
Pseudochirops cupreus	TACGTGTGCTGAGAAACTAAGAAAGTAGGAAGCAATCTAG	1138
Phalanger gymnotis	CATGTGTGCTGACAAAGTAAGAAAGTAGGAAACAATCTAG	1143
Pseudocheirus occidentalis	CATGTGTGCTGAGAAACTAAGAAAGTAGGAAGCAATCTAG	1142
Pseudocheirus peregrinus	CATGTGTGCTGAGAAACTAAGAAAGTAGGAAGCAATCTAG	1142
Petauroides volans	CATGTATGCTGAGAAACTAAGAAAGTAGGAAGCAATCTAG	1160
Vombatus ursinus	TGTGAGTGGTGAGAAACTAAGAAAGTAGGAAACAATCTAG	1144

	1170 1180 1190 1200	
Petaurus breviceps	TGAAATACAAACAATTGATACCTGTATTCATGGAAGACCT	1173
Acrobates pygmaeus	TGAAATGCAAACAATTGATACTTTCATTTATGAAAGACCT	1200
Distoechurus pennatus	TGAAACGCAAACAATTGATACCTTCATTCATGGAAGACAT	1190
Dactylopsila trivirgata	TGAAATACAAACAATTGATACCTTTATTGACGGAAGACCT	1174
Macropus giganteus	TGAAATTCAGACAATTGTTATCTTTATTCATGTTAGACTT	1175
Pseudochirops archeri	TGAAATACAAACAATTGATACCTTTATTCACGGAAGACCT	1184
Phascolarctos cinereus	TGAAATGCAAACAGTTGATACCTTTATTTACGGAAGACCT	1154
Pseudochirops corinnae	TGAAATACAAACAGTTGATACCTTTATTCACGGAAGACCT	1171
Pseudochirops cupreus	TGAAATACAAACAATTGATACCTTTATTCACGGAAGACCT	1178
Phalanger gymnotis	TGAAATGCAAACAATTGATACCTTTATTCACGGAAGACCT	1183
Pseudocheirus occidentalis	CAATCTACAAACAATTGATACCCTTATTCATGGAAGACCT	1182
Pseudocheirus peregrinus	CAATCTACAAACAATTGATACCTTTATTCATGGAAGACCT	1182
Petauroides volans	TGAAATACAAACAATCGATACCTTTATTCACGGAAGACCT	1200
Vombatus ursinus	TGAAATGCAAACAATTGATACCTTTATTTACAGAAGACCT	1184

	1201–1240	
Petaurus breviceps	TAGGCTAAATTCACCATTTAGGAATCCTAGATACATCCCA	1213
Acrobates pygmaeus	TTGGTTAATTTCACCATTTGGGAGTTCTAGATATATCCTA	1240
Distoechurus pennatus	TTGGCTAATTTCACCATTTGGGAGTTCTAGATATATCCCA	1230
Dactylopsila trivirgata	CAGGCTAATTTCACCATTTGGGAGTCCTAGATACATCCCA	1214
Macropus giganteus	CTGCCTAATTTCACCATTTGGGAGTTCTAGATAGATCCCA	1215
Pseudochirops archeri	CTGGTTAATTTCACTGTTTAGGAGTCCTCGATATATCCCA	1224
Phascolarctos cinereus	CTGGCTAATTTCACCATTTGGGAGTTCTAGATACATCGAA	1194
Pseudochirops corinnae	CTGGTTAATTTCACCGTTTGGGAGTCCTCGATATATCCCA	1211
Pseudochirops cupreus	CTGGTTAATTTCACCGTTTGGGAGTCCTCGATATATCCCA	1218
Phalanger gymnotis	CTGGCTAATTTCACCATTTGGGAGTCCTAGATATATTCCA	1223
Pseudocheirus occidentalis	CTGGCTAATTTCACCATTTGGGAGTCCTCGATATATCCCA	1222
Pseudocheirus peregrinus	CTGGCTAATTTCACCATTTGGGAGTCCTCGATATATCCCA	1222
Petauroides volans	CTGACTAATTTTACCGTTTGGGAGTCCTTGATATATGCCA	1240
Vombatus ursinus	CTGGCTAATTTCACCATTTGGAAGTCCTAGATACATCGCA	1224

	1241–1280	
Petaurus breviceps	GGCTACACACACTCCATTTTTTAATACCCATATTTCAGAT	1253
Acrobates pygmaeus	GTAAATGCACACTTCATTTTTTAATACCCGTATTTTAGAT	1280
Distoechurus pennatus	GTCAATGCACACTTCATTTTTTAATACCCGTATTCCAGAT	1270
Dactylopsila trivirgata	GTCGACGCACACTCCATTTTTTAATACCCATATTTCAGAT	1254
Macropus giganteus	GTCAGAGCACACTTCATTTTTCAATGCACGTATTCCAGAT	1255
Pseudochirops archeri	GTCTACGCACACTTCATTTTTTAATACCCATATTTCAGAT	1264
Phascolarctos cinereus	GTCGATGCACACTTCATCTTTTAATACCCGTATTCCAGAT	1234
Pseudochirops corinnae	GTCGACGCACACTTCATTTTTTAATACCCATATTTCAGAT	1251
Pseudochirops cupreus	GTCGACGCACACTTCATTTTTTAATACCCATATTTCAGAT	1258
Phalanger gymnotis	GTCAATGCACACTTCATTTTTTAATACTCGTATTCCAGAT	1263
Pseudocheirus occidentalis	GTCGACGCACACTTCATTTTTTAATACCCATATTTCAGAT	1262
Pseudocheirus peregrinus	GTCGACGCACACTTCATTTTTTAATACCCATATTTCAGAT	1262
Petauroides volans	GTCGACGCACACTTCA-TTTTTAATACCCATATTTCAGAT	1279
Vombatus ursinus	GTCGATGCACACTTCATCTTTCAATACCCGTATTCCAGAT	1264

	1281–1320	
Petaurus breviceps	TTATAGCACGCCCTCAGCACACTGTGACTCGTTCTATATA	1293
Acrobates pygmaeus	TTATAGTATGCCATCAGCACACTGTGACTCGTTCTATATA	1320
Distoechurus pennatus	TTATAGCATGCCATCAGCACACTATGACTCGTTCTATATA	1310
Dactylopsila trivirgata	TTATAGCATGCCATCAACACACTGTGACTCGTTCTATATA	1294
Macropus giganteus	TTATAGTATGCCACCAGCACACTGTGAGTCGTTCTATATA	1295
Pseudochirops archeri	TTATAGCATGCCATCAGTACACTGTGATTCGTTCTATATA	1304
Phascolarctos cinereus	TTATAGCATGCCATCAATACTCTATGACTCGTTCTATATA	1274
Pseudochirops corinnae	TTATAGCATGCCATCAGTACACTGTGATTCGTTCTATATA	1291
Pseudochirops cupreus	TTATAGCATGCCATCAGTACACTGTGATTCGTTCTATATA	1298
Phalanger gymnotis	TTGTAGCACAGCATCAGCACCCTGTGACTCGTTCTGTATA	1303
Pseudocheirus occidentalis	TTATAGCATGCCATCAGTACACTGTGATTCGTTCTATATA	1302
Pseudocheirus peregrinus	TTATAGCATGCCATCAGTACACTGTGATTCGTTCTATATA	1302
Petauroides volans	TTATAGCATGCCATGAGTACACTGTGATTCGTTCTATATA	1319
Vombatus ursinus	TTATAGCATGCCATCAATACTCTATGACTCGTTCTATATA	1304

	1330 1340 1350 1360	
Petaurus breviceps	GCTAGAGGTGAGCCCGGAGCTTGAGTCTCTAAATGCCTCA	1333
Acrobates pygmaeus	GCTAGAGGTGAGCCTGGAGTTTAAGTCTCTAAATGCCTCA	1360
Distoechurus pennatus	GCTAGAGGTGAGCCTGGAGCTTGAGTCTCTAAATGCCTCA	1350
Dactylopsila trivirgata	GCTA-AAGTGAGCTCGGAGCTTGAGTCTCTAAATGCCTCA	1333
Macropus giganteus	GCTAGAGGTGAGCCCTGAGTTTGAGTCCCTAAATGTCTC-	1334
Pseudochirops archeri	GCTA------------GAGCTTGAGTCTCTAAATGCCTCA	1332
Phascolarctos cinereus	GCTACAGGTGAGCCCTGAGTTTGCGTCTCTAAATGCCTCA	1314
Pseudochirops corinnae	GCTAGAGGTGAGCCCGGAGCTCGAATCTCTAAATGCCACA	1331
Pseudochirops cupreus	GCTAGAGGTGAGCCCGGAGCTTGAGTCTCTAAATGCCACA	1338
Phalanger gymnotis	GCCAGAGGTGAGCCCGCAGTTTGAATCTCTAAATGCCTCA	1343
Pseudocheirus occidentalis	GCTAGAGGCGACCCCGGAGCT------------TGCCTCA	1330
Pseudocheirus peregrinus	GCTAGAGGCGACCCCGGAGCT------------TGCCTCA	1330
Petauroides volans	GCTAGAGGTGTGCCCGGAGCTTG--------AATGCCTCA	1351
Vombatus ursinus	GCTAGAGGTGAGCCCTGAATTTGAGTCTCTAAATGCCTCA	1344

	1370 1380 1390 1400	
Petaurus breviceps	ATTAGTCAATCCTGAAATAAATGTATATTAGACACGAGGT	1373
Acrobates pygmaeus	ATT----AATCCTGACATAAATGTATATTAGACATTAGGT	1396
Distoechurus pennatus	ATTAATCAATCCTGACATAAATGCGTATTAGACACTAGGT	1390
Dactylopsila trivirgata	ATTAATCAATCCTGACATAAATGTATATTAGACACGAGGT	1373
Macropus giganteus	---AATCAATCCTGACATAAATGTATATTAGACACAAGGT	1371
Pseudochirops archeri	ATTAATCAATCCTGACATAAATGTCTA--ATACACGAGGT	1370
Phascolarctos cinereus	ATTAATCAATCTTGACATACATGTATATTAGACACAAGGT	1354
Pseudochirops corinnae	ATTAATCAATCCTGACATAAATGTATATTAGACACGAGGT	1371
Pseudochirops cupreus	ATTAATCAATCCTGACATAAATGTATATTAGACACGAGGT	1378
Phalanger gymnotis	ATTAATCAATCCTGACATAAACGTATATTAGACACAAGGT	1383
Pseudocheirus occidentalis	ATTAATCAATCCTGACATAAATGTATATTAGACACGAGGT	1370
Pseudocheirus peregrinus	ATTAATCAATCCTGACATAAATGTATATTAGACACGAGGT	1370
Petauroides volans	ATTAATCAATCCTGACATAAATGTATATTAGACACGAGGT	1391
Vombatus ursinus	ACTAATCAATCCTGACATAGATGTTTATTAGACACAAGGT	1384

	1410 1420 1430 1440	
Petaurus breviceps	ACTTCTGGTCTGCATCATACCATATGTTACATACACCAAA	1413
Acrobates pygmaeus	GCTTTGAATCTGCATCATACCATATGTTACATACACCAAA	1436
Distoechurus pennatus	GCTTCGAGTCTGCATCATACCATATGTTACATACACCAAA	1430
Dactylopsila trivirgata	GCTTCGCGTCTGCATCATACCATATGCTACCTACACCGAA	1413
Macropus giganteus	GCTTCCCGTCTGCATCATTCCATATGCTACATACACCAAA	1411
Pseudochirops archeri	GCTTCGCCTCTGCATCATACTATATGTTACGTACACCAAA	1410
Phascolarctos cinereus	GCTTCGCTTCTGCACTATGCCATATGTTACATACACCAAA	1394
Pseudochirops corinnae	GCTTCGCCTCTGCATCATACTATATGTTACATACACCAAA	1411
Pseudochirops cupreus	GCTTCGCCTCTGCATCATACTATATGTTACATACACCAAA	1418
Phalanger gymnotis	GCTTCGCGCCTGCATCATACCATATGTTACATACACCAAA	1423
Pseudocheirus occidentalis	GCTTCGAGTCTGCATCATACTACATGTTACATA---CAAA	1407
Pseudocheirus peregrinus	GCTTCGAGTCTGCATCATACTACATGTTACATA---CAAA	1407
Petauroides volans	GCTTCGCGTCTGCATCATACCATATGCTACATACACTAAA	1431
Vombatus ursinus	GCTTCGCGTCTGCGCTATGCCGTATGTTACATACACCAAA	1424

	1450 · 1460 · 1470 · 1480	
Petaurus breviceps	TATTGCCCTTGGTAACAATTCTTCGGGCAAGAAAGAGCTC	1453
Acrobates pygmaeus	TATTGCCCTTGATAACAATTCTTCGGGCAAGAAAGAGTTC	1476
Distoechurus pennatus	TATTGTCCTTGATAACAATTCTTCGGGCAAGAAAGGGATG	1470
Dactylopsila trivirgata	TATTGCCCTTGATAACAATTCTTCCGGCAAGAAAGAGCTC	1453
Macropus giganteus	TATTGCCCTTAATAACAATTCTTCGGGCAAGAAAGAGCTC	1451
Pseudochirops archeri	TATTGCCCTTGATAACAATTCTTCGGGC----AAGAGCTC	1446
Phascolarctos cinereus	TACAGCCCTTGATTACAATTCTTCCGGCAAGAAAGAGTTC	1434
Pseudochirops corinnae	TATTGCCCTTGATAACAATTCTTCGGGCAAGAAAGAGCTC	1451
Pseudochirops cupreus	TATTGCCCTTGATAACAATTCTTCGGGCAAGAAAGAGCTC	1458
Phalanger gymnotis	TATTGCCCTTGATAACAATTCTCCGAGCAAGAAAGAGCTC	1463
Pseudocheirus occidentalis	TATTGGCCTTGATAACAATTCTTCGGGCAAGAAAGAGCTC	1447
Pseudocheirus peregrinus	TATTGGCCTTGATAACAATTCTTCGGGCAAGAAAGAGCTC	1447
Petauroides volans	TATTGCCCTTGATAACAATTCTTCGGGCAAGAAAGAGCTC	1471
Vombatus ursinus	TATAGCCCTTGATAACAATTCTTCGGGCAAGAAAGAGTTT	1464

	1490 · 1500 · 1510 · 1520	
Petaurus breviceps	GCTTCCACGTAGACCAGATCTGGGAAAAGGAAGGAAATGG	1493
Acrobates pygmaeus	GCTTCCATGTAGCCCAGATCTGGGAAAAGGAAGGAAATGG	1516
Distoechurus pennatus	ACTTCCATGTAGCCCAGATCTGGGAAAAGGAAGGAAACGG	1510
Dactylopsila trivirgata	GCTTCCATGTAGCCCAGATCTGGGAAAAGGAAGGAAATGG	1493
Macropus giganteus	GCTTCCATGTAGCCCAGATCTGGGAAAAGGAAGGAGATGG	1491
Pseudochirops archeri	TCTTCCATGTAGCCCAGATCTGGGAAAAGGAAGGAAATGG	1486
Phascolarctos cinereus	GCTTCCATGTAGCCCAGATCTGGGAAAAGGAAGGAAATGG	1474
Pseudochirops corinnae	TCTTCCATGTAGCCCAGATCTGGGAAAAGGAAGGAAATGG	1491
Pseudochirops cupreus	TCTTCCATGTAGCCCAGATCTGGGAAAAGGAAGGAAATGG	1498
Phalanger gymnotis	GCTTCCATGTAGCCTAGATCTGGGAAAAGGCAGAAAATGA	1503
Pseudocheirus occidentalis	GCTTCCATGTCGCCCAGATAAGGGGAAAGGAAGGAAATGG	1487
Pseudocheirus peregrinus	GCTTCCATGTCGCCCAGATAAGGGGAAAGGAAGGAAATGG	1487
Petauroides volans	GCTTCCACGTAGCCCAGATATGGAAAAAGGAAGCAAATGG	1511
Vombatus ursinus	GCTTCCGTGTAGCCCAGATCTGGGAAAAGGAAGGAAATGG	1504

	1530 · 1540 · 1550 · 1560	
Petaurus breviceps	AAAAACAAAACAAGATCACGTTGAAAAATCCCACCCTGGC	1533
Acrobates pygmaeus	AAAGACAAAACAAGATCATGTTCAAGAATCCAACCCTGGG	1556
Distoechurus pennatus	AAAGACAAAACAAGATCATCTTCAAGAATCCCACCCCCGG	1550
Dactylopsila trivirgata	AAAAGCAAAACAAGATCACGTTAAAGAATCTCACCCTGGG	1533
Macropus giganteus	AAAAACAAAACAAGATCACGTTCAAGAATCCCACCCTGGG	1531
Pseudochirops archeri	AAAAACAAAACGAGATCACGTTAAAGAATCCCACCTTGGG	1526
Phascolarctos cinereus	AAAAACAAAACAAGATCACGTTCTGGAATCCCACCCTGGG	1514
Pseudochirops corinnae	AAAAACAAAACAAGATCACGTTAAAGAATCCCACCTTGGG	1531
Pseudochirops cupreus	AAAAACAAAACAAGATCACGTTAAAGAATCCCACCTTGGG	1538
Phalanger gymnotis	AAAAAAAAAACAAGATCACGTTCAGGAATCCCACCGTGGG	1543
Pseudocheirus occidentalis	AAAAACAAAACAACATCATGTTAAAGAATCCCACCTTGGG	1527
Pseudocheirus peregrinus	AAAAACAAAACAACATCATGTTAAAGAATCCCACCTTGGG	1527
Petauroides volans	AAAAACGAAACAAGACCACGTTAAAGAATCCCACCTTGGG	1551
Vombatus ursinus	AAAAACAAAACAGGATCATGTTCTGGAATCCCACACTGGG	1544

Species	1570 · 1580 · 1590 · 1600	
Petaurus breviceps	AATCTTGTTCGAAGACATCTCGGAAACCTTGACTAGGCTG	1573
Acrobates pygmaeus	AATCTTGTCTGAAGACATCTCGGAAACCTTGACTAGGCTG	1596
Distoechurus pennatus	AATCT--TTTGAAGACATCTCGGAAACCTTGACTAGGCTG	1588
Dactylopsila trivirgata	AATCTTGTTTGAAGACATCTTGGAAACCTTGACTAGGCTG	1573
Macropus giganteus	AATCCTGTTTGAAGACATCTCGGAAACCTTGCCTGGGCTG	1571
Pseudochirops archeri	AATCTTGTTTGAAGACATCTCGGAAACCTTGACTAGGCTG	1566
Phascolarctos cinereus	AATCTTGTTTGAGGACATGTCGGAAACCTAGGCTAGGGAG	1554
Pseudochirops corinnae	AATCTTGTTTGAAGACATCTCGGAAACCTTGACTAGGCTG	1571
Pseudochirops cupreus	AATCTTGTTTGAAGACATCTCGGAAACCTTGACTAGGCTG	1578
Phalanger gymnotis	AATCTTGTTTGAAGACATCTCGGAAACCTTGGCTAGGCTG	1583
Pseudocheirus occidentalis	AATCTTGTTTGAAGACATTTCGGAAACCTTGACTAGGCTG	1567
Pseudocheirus peregrinus	AATCTTGTATGAAGACATCTCGGAAACCTTGACTAGGCTG	1567
Petauroides volans	AATCTTGTTTGAAGACATCTCGGAAACCTTGACTAGGCTG	1591
Vombatus ursinus	AATCTTGTTTGAGGACATCTCGGAAACCTAGACTAGGGAG	1584

Species	1610 · 1620 · 1630 · 1640	
Petaurus breviceps	CTTCCCTCTTCTCACTGCCTTGTTAGACGTGCTCCATGGT	1613
Acrobates pygmaeus	TTT---TCTTCCTAATGCCTTGTTAGACGTGCTCCATGGT	1633
Distoechurus pennatus	TTTCCCTCTTCCCACTGCC-TGTTAGACGTGCTCCATGGT	1627
Dactylopsila trivirgata	-------CTTCCCATTGCCTTGTTAGACGTGCTCCATGGT	1606
Macropus giganteus	CTTCCCTCTTCCCACTGCCTTGTTAGATGTGCTCCATAGT	1611
Pseudochirops archeri	CTTCCCTATTCCCACTGCCTTGTCAGACGTGCTCCATGGT	1606
Phascolarctos cinereus	ATTCCCTCTTCCCATAGCCTTGTTAGAAGTGCTCCATGGT	1594
Pseudochirops corinnae	CTTCCCTCTTCCCACTGCCTTGTCAGACGTGCTCCATGAT	1611
Pseudochirops cupreus	CTTCCCTCTTCCCACTGCCTTGTCAGACGTGTACCATGGT	1618
Phalanger gymnotis	CTTCCCTCTTCCCACCGCCTTGTTAGACGTGCTCCAAGGT	1623
Pseudocheirus occidentalis	CTTCCCTCTTCCCACTGCCTTGTTAGACGTGCTCCATGGT	1607
Pseudocheirus peregrinus	CTTCCCTCTTTCCACTGCCTTGTTAGACGTGCTCCATGGT	1607
Petauroides volans	CTTCCCTCTTCTCACTGCCTTGTTAGACGTGCTCCATGGT	1631
Vombatus ursinus	ATTCCATCTTCCCACAGCCTTTTTAGAAGTGCTCCGTGGT	1624

Species	1650 · 1660 · 1670 · 1680	
Petaurus breviceps	TTGTAAAGGAGTGACCAAGAACTATGGCACTGGTCCCAGA	1653
Acrobates pygmaeus	TTGTA-----------AAGAACTATAGCACTGTCTTGAAC	1662
Distoechurus pennatus	TTGTA-----------AAGAACTAAAGCACCGGTCCCAGA	1656
Dactylopsila trivirgata	TTGTAAAGGAGTGACCAAGAACTATGGCACTGGTCCCAGA	1646
Macropus giganteus	TT---AAGGAGTGACCAAGAACTAGAGGACTGGTCTTGCC	1648
Pseudochirops archeri	TTATAAAGGAGTGACCAAGAACTATGGCATTGGTCCCAGA	1646
Phascolarctos cinereus	TTGTAAAGGAGTGACCAAGAATTACAGCACTGGTCCCAGA	1634
Pseudochirops corinnae	TTATAAAGGAGTGACCAAGAACTATGGCACTGGTCCCAAA	1651
Pseudochirops cupreus	TTATAAAGGAGTGACCAAGAACTATAGCACTGGTCCCAGA	1658
Phalanger gymnotis	TTGTAAA-GAGTGACCAAGAACTAGAGCACTAGTCCCAGA	1662
Pseudocheirus occidentalis	TTATAACGGAGTGACCAA--------GCACTGGTCCCAGA	1639
Pseudocheirus peregrinus	TTATAACGGAGTGACCAA--------GCACTGGTCCCAGA	1639
Petauroides volans	TTATAAAGGAGTGACCAAGAACTATGGCACTGGTCCCAGA	1671
Vombatus ursinus	TTGTAAAGGAGTGACCAAGAATTACAGCACTGGTCCCAGA	1664

```
                              1690      1700      1710      1720
Petaurus breviceps            AAAGACTACCTCAAGCCAGGGTCTACCCTTTGCCTTGAAC  1693
Acrobates pygmaeus            AATATT----------------------------------  1668
Distoechurus pennatus         AAAGACTACCTCAAGCCAGGTTCGACCCTTAGACTTGAAC  1696
Dactylopsila trivirgata       AAAGACTACCTCAAGCCAGGGTCGACCCTTTGCCTTGAAC  1686
Macropus giganteus            TTGAACAATCTTAA--------------------------  1662
Pseudochirops archeri         AAAGACTACCTCAAGCCAGGGTCGACCCTTTGCTTTGAAC  1686
Phascolarctos cinereus        AAAGACTACCTCAAGCCACGGTCTACCCTTTGCCTTGAAA  1674
Pseudochirops corinnae        ACAGACTACCTCAGGCCACGGTCGACCCTTTGCTTTGAAC  1691
Pseudochirops cupreus         AAAGACTACCTCAGGCCAGGGTCGACCCTTTGCTTTGAAC  1698
Phalanger gymnotis            AAAGA----CTCAAGCCAAGGTCGACCCTTTGCCTTGAAC  1698
Pseudocheirus occidentalis    AAAGACTACTTCAAGCCAGGGTCGACCCTTTGCCTTGAAC  1679
Pseudocheirus peregrinus      AAAGACTACTTCAAGCCAGGGTCGACCCTTTGCCTTGAAC  1679
Petauroides volans            AAAGACTACCTCAAGCCAGGGTCGGTCCTTTTCCTTGAAC  1711
Vombatus ursinus              AAAGACTACCTCAAGCCAGGGTCGACCCTTTGCCTTGAAA  1704

                              1730
Petaurus breviceps            AATCTTTTAA  1703
Acrobates pygmaeus            ----------  1668
Distoechurus pennatus         AATATTTTAA  1706
Dactylopsila trivirgata       AATCTTTTAA  1696
Macropus giganteus            ----------  1662
Pseudochirops archeri         AGTCTTTGAA  1696
Phascolarctos cinereus        A---TCTCAA  1681
Pseudochirops corinnae        AGTCTTTGAA  1701
Pseudochirops cupreus         AGTCTTTGAA  1708
Phalanger gymnotis            AATCTT--AA  1706
Pseudocheirus occidentalis    AGTGTTTGAA  1689
Pseudocheirus peregrinus      AGTGTTTGAA  1689
Petauroides volans            AGTCTTTGAA  1721
Vombatus ursinus              A---TCTCAA  1711
```

GAR 16519

	Positions 1–40 (10, 20, 30, 40)	
Petaurus breviceps	CCCCCAAATTGTTCAAATCCCAGTTCTGCTACTTCCATCC	40
Acrobates pygmaeus	CCCCCAAATTGTTCAAATCTCAGTTCTGCTACTTACAGCC	40
Distoechurus pennatus	CCCCCAAATTGTTCGAATCCCAGTTCTGCTACTTACAGCC	40
Dactylopsila trivirgata	CCCCCAAATTGTTCAAATCCCAGTTCTGCTACTTACATCC	40
Macropus eugenii	ACCCCAAATTGTTCCAATCCAAATTCTGCTACTTACAGCC	40
Macropus fuliginosus	ACCCCAAATTGTTCCAATCCAAATTCTGCTACTTACAGCC	40
Macropus giganteus	ACCCCAAATTGTTCCAATCCAAATTCTGCTACTTACAGCC	40
Pseudochirops archeri	CCCCCAAATTGTTCTTACCCCAGTTCTACTACTTACATCC	40
Phascolarctos cinereus	CCCCCAAATCTTCAGAATCCCAGGTGTG----GTACAGCC	36
Pseudochirops corinnae	CCCCCAAATTGTTCTTACCCCAGTTCTGCTACTTACATCC	40
Pseudochirops cupreus	CCCCCAAATTGTTCTTACCCCAGTTCTGCTACTTACATCC	40
Phalanger gymnotis	CCCCTAAATTGTTCGGATCCCAGTTCAGCTCCTTACAACC	40
Pseudocheirus occidentalis	CCCCCCAATTGTTCTTATCCCAGTTCTGTTACTTACATCC	40
Pseudocheirus peregrinus	CCCCCCAATTGTTCTTATCCCAGTTCTGTTACTTACATCC	40
Petauroides volans	CCCCCAAATTGTTCTTATCCCAGTTCTGCTACTTACATCC	40
Tarsipes rostratus	CCCCCAAATTGTTCGAACTCCAGTTCTGCTATTTTGATCC	40
Vombatus ursinus	---------------AATCCCAGCTCAG----GTACAGCC	21

	Positions 41–80 (50, 60, 70, 80)	
Petaurus breviceps	TATGTGACTTTAGGCAAATTACTTCCCCTTTTCTGAGA--	78
Acrobates pygmaeus	TATGTGACTTTAGGCAAATCATTTCCCCTTTTCTGAGACT	80
Distoechurus pennatus	TATGTGACTTTAGGCAAATCACTTTCCCTTTTCTGAGACT	80
Dactylopsila trivirgata	TGTGTGACTTTAGGCAAATTACTT-CCCTTTTCTGAGACT	79
Macropus eugenii	TATGTGACTTTAGGCAAGTCAGTTCCCCTTTTCTGAGGCT	80
Macropus fuliginosus	TATGTGACTTTAGGCAAGTCACTTCCCCTTTTCTGAGGCT	80
Macropus giganteus	TATGTGACTTTAGGCAAGTCACTTCCCCTTTTCTGAGGCT	80
Pseudochirops archeri	ACTGTGACTTTAGGTAAATCACTTCTCCTTTTCTGAGACT	80
Phascolarctos cinereus	TATGTGACTTTAGGCGAGTCACTTCCCCTTTTCTGAGACT	76
Pseudochirops corinnae	TGTGTGATATTAGGCAAATCACTTCCCCTTTTCTGAGACT	80
Pseudochirops cupreus	TATGTGACATTAGGCAAATCACTTCCCCTTTTCTGAGACT	80
Phalanger gymnotis	TGTGTGACTTTAGGCAAGTCAATTCCCCTTTTCTGAGACT	80
Pseudocheirus occidentalis	TGTATGACTTTAGGCAAATCACTTCCCCTTTTCTGAGACT	80
Pseudocheirus peregrinus	TGTATGACTTTAGGCAAATCACTTCCCCTTTTCTGAGACT	80
Petauroides volans	TATATGACTTTAGGCAAATCACTTCCCCTTTTCTGAGATT	80
Tarsipes rostratus	TATGTAACTTTAGGCAAATCACTTCCCTTTTTCTGAGACT	80
Vombatus ursinus	TATGTGACTTTAGGCAAGTCACTTCCCCGTTTCTGAGACT	61

	90 100 110 120	
Petaurus breviceps	CTATTTCGTCATCTGCTAAATTAAGTTTAACTGGATTACC	118
Acrobates pygmaeus	CAATTTCCACATCTGCTAAATTAAGTTTAACTAGATTA-C	119
Distoechurus pennatus	CAATTTCCACATCTCCTAAATTAAGTTTAACTAGATTACC	120
Dactylopsila trivirgata	CTATTTCCTCATCTGCTAAATTAAGTTTAACTAGATTACC	119
Macropus eugenii	CCATTTTCTCATGTGCCAAATTAAATTTAACTAGATTACC	120
Macropus fuliginosus	CCATTTTCTCATCTGCCAAATTAAGTTTAACTAGATTACC	120
Macropus giganteus	CCATTTTCTCATCTGCCAAATTAAGTTTAACTAGATTACC	120
Pseudochirops archeri	CTATTTCCTCATCTGCTGAATTAAGTTTAACTAGATTACC	120
Phascolarctos cinereus	CAATTTCCTCACCTGCCAAA-GAGGTTTACCTAGATGACC	115
Pseudochirops corinnae	CTATTTCCTCATCTGCTAAATTAAGTTTTACTAGATTACC	120
Pseudochirops cupreus	CTATTTCCTCATCTGCTAAATTAAGTTTAACTAGATTACC	120
Phalanger gymnotis	CAATTTCTTCATCTGCTAAATTAAGTTTAACTAGATTACC	120
Pseudocheirus occidentalis	CTATTTCCTCATCTGCTAAATTAAGTTTAACTAGACTACC	120
Pseudocheirus peregrinus	CTATTTCCTCATCTGCTAAATTAAGTTTAACTAGACTACC	120
Petauroides volans	CTATTTCCTTGTCTGCTAAATTAAGTTTAACTAGACTACC	120
Tarsipes rostratus	CTATTTCCTCAACTGCTAAATTAATTTTAACTAGATTACC	120
Vombatus ursinus	CAATTTTCTCATCTGCCAAATGAGGTTTAACTAGATGACC	101

	130 140 150 160	
Petaurus breviceps	CTTAGGCACGACCTGTCAAATCACCCTTTATTATAAAGAG	158
Acrobates pygmaeus	CTTAGGCACGATCTGTCCAATCACACATTATTATAAAGGG	159
Distoechurus pennatus	TTAG----------GTCCAATCACCGTTTATTATAAAGAT	150
Dactylopsila trivirgata	CTTAGGCAAGACCTGTCCAATCACCCTTTATTATAAAGGG	159
Macropus eugenii	TTTAGCCATGACCTGTCCAATCACCCTTTATTATAAAGAG	160
Macropus fuliginosus	CTTAGCCATGACCTGTCCAGTCACCCTTTATTATAAAGAG	160
Macropus giganteus	CTTAGCCATGACCTGTCCAGTCACCCTTTATTATAAAGAG	160
Pseudochirops archeri	CTTAGGCACGACCTGTCCAATCACCCTTTATTATAAAGGG	160
Phascolarctos cinereus	CTTAGGCATGACCTGTCCAATGAC----------------	139
Pseudochirops corinnae	CTTAGGCACGACCTGTCCAATCACCCTTTATTATAAAGGG	160
Pseudochirops cupreus	CTTATGCACGACCTGTCCAATCACCCTTTATTATAAAGGG	160
Phalanger gymnotis	CTTAGGCATGACCTCTCCAATCAGCCTTTATTATAAAGGG	160
Pseudocheirus occidentalis	CTCAGGCACGACCTGTCCAATCACCCTTTATTATAAAGGG	160
Pseudocheirus peregrinus	CTCAGGCACGACCTGTCCAATCACCCTTTATTATAAAGGG	160
Petauroides volans	CTTAGGCACGACCTGTCCAATCACCCTTTATTATAAAGGG	160
Tarsipes rostratus	CTTAGGCACGACCTGTCCGATCACCCTTTATTATAAAGGG	160
Vombatus ursinus	CTTAGGCATGACCGGTCCAACGGAC---------------	126

	170 / 180 / 190 / 200	
Petaurus breviceps	AACCCTTCCCTTCTTCAAACTCTACTCCCTCCCCCATAGT	198
Acrobates pygmaeus	GACCCTTTTCTTC-TCAAACTCTATCCCTTCCCCCATAGT	198
Distoechurus pennatus	GACCCTTCTCTTTTTCAAACTCTACCCCTTCCCCCTTAGT	190
Dactylopsila trivirgata	AACCCTTCCCTTCTTCAAACTCAACTCCCTCCCCCATAGT	199
Macropus eugenii	GACTCTTCCCTTCTTCAAATTCTACCCCCTCCCCCGTAAT	200
Macropus fuliginosus	GACTCTTCCCTTCTTCAAATTCTACCCCCTCCCCCGTAAT	200
Macropus giganteus	GACTCTTCCCTTCTTCAAATTCTACCCCCTCCCCCGTAAT	200
Pseudochirops archeri	AACCCTTCCCTTCTTCAAACTCTATCCCTTCCCCCATAGT	200
Phascolarctos cinereus	-------CCCTTCTTCAAACTCTACCCCCTCACCCGTAGT	172
Pseudochirops corinnae	AACCCTTCCCTTCTTCCAACTCTATTCCTTCCCCCATAGT	200
Pseudochirops cupreus	AGCCCTTCCCTTCTTCAAACTCTATTCCTTCCCCCATAGT	200
Phalanger gymnotis	GACCCTTCCCTTCTTCAAACTCTACCCCCTCCCCCATAGT	200
Pseudocheirus occidentalis	AAGCCTTCCCTTCTTCAGCCTCTATTCCCTCCCTGATAGT	200
Pseudocheirus peregrinus	AAGCCTTCCCTTCTTCAGCCTCTATTCCCTCCCTGATAGT	200
Petauroides volans	AACCCTTCCCTTCTTCAAACTCTATTCCCTCCCCCATAGT	200
Tarsipes rostratus	AACCCTTACCTTCTCCAAACTCTACTCCCTCCCCCATAGT	200
Vombatus ursinus	-------CCCTTCTTCAAACTCTACCCCCTCAGCCATAGT	159

	210 / 220 / 230 / 240	
Petaurus breviceps	TATAGGAAATAATGAATAACTGTTAAGCCACAAGGTTCTT	238
Acrobates pygmaeus	TATAGGAAATAATGAATATATGCAAAGTCACAAGGTTCTT	238
Distoechurus pennatus	TACAGGAAATAATGAATAAGTGAAAAGTCACAAGGTTCTT	230
Dactylopsila trivirgata	TATAGGAAATAATGAATAACTGCCAAGCCACATGGTTCTT	239
Macropus eugenii	TATGGGAAACCATGTATAACTGCAAAGTCATAGGGTTTTT	240
Macropus fuliginosus	TATGGGAAATAATGTATAACTGAAAAGTCATAGGGTTTTT	240
Macropus giganteus	TATGGGAAATAATGTATAACTGAAAAGTCATAGGGTTTTT	240
Pseudochirops archeri	TATAGGAAATAATGAATAACTGCAGAGTCACAAGGTTCTT	240
Phascolarctos cinereus	TATAGGAAATAATGAATAACTGCAAAGCCATGAGATTCTT	212
Pseudochirops corinnae	TATAGGAAATAATGAATAACTGCAGAATCACAAGGTTCCT	240
Pseudochirops cupreus	TATAGGAAATAATGAATAACTGCAGAGTCACAAGGTTCTT	240
Phalanger gymnotis	TATAGGAAATAATGAATAACCTCAAAGTCACAAGGTTCTT	240
Pseudocheirus occidentalis	TATAGGAAATTATGAATAACTGCAAAGTCACAAGGTTCTT	240
Pseudocheirus peregrinus	TATAGGAAATTATGAATAACTGCAAAGTCACAAGGTTCTT	240
Petauroides volans	TATAGGAAATTATGAATAACTGCAAAGTCACAAGGTTCTT	240
Tarsipes rostratus	TATAGGAAATAATGAATAACTTCAAAGTCTCAAGGTTCTT	240
Vombatus ursinus	TATAGGAAATAATGAATAACTGCAAAGCCATAAGATTCTT	199

	250 260 270 280	
Petaurus breviceps	AGATTCATCTGAATTTAGGGTATGAACTAAATTCTTTTGT	278
Acrobates pygmaeus	AGATCCACCTGAGTTTAGGGTATCAACTGAATTCTTTTGT	278
Distoechurus pennatus	AGATCTATTTGAGTTTAGGGTATCAACTGAATTCTTTTGT	270
Dactylopsila trivirgata	AGATTCATTTGAGTTTAGGGTATGAACTGAATTCTTTTGT	279
Macropus eugenii	AGATCCATTTGAATATAGGTTATGAACTGAATTCTTTTGT	280
Macropus fuliginosus	AGATCCATTTGAATATAGGGTATGAACTGAATTCTTTTGT	280
Macropus giganteus	AGATCCATTTGAATATAGGGTATGAACTGAATTCTTTTGT	280
Pseudochirops archeri	AGATTCATTTGAGTTTAGGGTATGAACTGAATTCTTTTGT	280
Phascolarctos cinereus	AGCTCCATTTGAATTTAGGGTATGAACTGAATTCTTTTGT	252
Pseudochirops corinnae	AGATTCATTTGAGTTTAGGGTATGAACTGAATTCTTTTGT	280
Pseudochirops cupreus	AGATTCATTTGAGTTTAGGGTATGAACTGAATTCTTTTGT	280
Phalanger gymnotis	AGATCCGTTTGAGTTTAGCGTATGAACTGAATTCCTTTGT	280
Pseudocheirus occidentalis	AGATTCATTTGAGTTTAAGGTATGAACTGAATTCTTTTGT	280
Pseudocheirus peregrinus	AGATTCATTTGAGTTTAAGGTATGAACTGAATTCTTTTGT	280
Petauroides volans	AGATTCATTTGAGTTTAGGGTATGAACTGAATTCTTTTGT	280
Tarsipes rostratus	AGATTCATCTGAGTTTAGGGTATGAACTGAATTCATTTGT	280
Vombatus ursinus	AGATCCATTTGAGTTTAGGGTATGAACTGAATTCTTTTGT	239

	290 300 310 320	
Petaurus breviceps	AAACCAAAGAATAAAGCTACAACAATCTAACAGGGAAGGT	318
Acrobates pygmaeus	AAACCAAAGAATAAAACTACAACACCCTAACAGGGAAGGT	318
Distoechurus pennatus	AAACCAAAGAATAAAACTACAACAACCTAACAGGGAAGGT	310
Dactylopsila trivirgata	AAAGCAAAGAATAAAGCTACAACAACCTAACAGGGAAGGT	319
Macropus eugenii	AAACCAAGGAATAAAACTACAGCAACCTAACAGGGAAGGT	320
Macropus fuliginosus	AAACCAAGGAATAAAACTGCAGTAACCTAACAGGGAAGGT	320
Macropus giganteus	AAACCAAGGAACAAAACTGCAGTAACCTAACAGGGAAGGT	320
Pseudochirops archeri	AAACCAAAGAATAAAGCCACAACAACCTAACAGGGAAGGT	320
Phascolarctos cinereus	AAACCAAAGAATAAAACTACAACAACCTACCAGGGAAGGT	292
Pseudochirops corinnae	AAACCAAAGAATAAAGCCACAACAACCTAACAGGGAAGGT	320
Pseudochirops cupreus	AAATCAAAGAATAAAGCCACAACAACCTAACAGGGAAGGT	320
Phalanger gymnotis	AAACCAAAGAATAAAACTACAACAACCTAACAGGGAAGGT	320
Pseudocheirus occidentalis	AAACCAAAGAATAAAGCTACAACAACCTAAGAGGGAAAGT	320
Pseudocheirus peregrinus	AAACCAAAGAATAAAGCTACAACAACCTAAGAGGGAAAGT	320
Petauroides volans	AAACCAAAGAATAAAGCTACAACAACCTAACAGGGAAGAT	320
Tarsipes rostratus	AAACCAGAGAATTAAACCACAACAACCTAACAGGGATGGT	320
Vombatus ursinus	AAACCAAAGAATAAAATTACAACAACCTAACAGGGAAGGT	279

	330 340 350 360	
Petaurus breviceps	CTCTTAGGAGAGTTTAATTCAAATGTGGACCCCAAGTTCA	358
Acrobates pygmaeus	CTCTT---AGAGCTTGATTCAAATGTGGAATTCAAGTTCA	355
Distoechurus pennatus	CCCTTAGGAGAGCTTAATTCAAATGTGGAACTCAAGTTCA	350
Dactylopsila trivirgata	CTCTTAGGAGAGTTTAATTTAAATGTGGACCACAAGTTCA	359
Macropus eugenii	CTCTTGGGAGAGCTTAATTCAAATGTGGACCCCAAGTTCG	360
Macropus fuliginosus	CTCTTGGGAGAGCTTAATGCAAATATGGACCCCAAGTTCA	360
Macropus giganteus	CTCTTGGGAGAGCTTAATGCAAATATGGACCCCAAGTTCA	360
Pseudochirops archeri	CTCTTAGGAGAGTTTAATTCAAATGTGGACCCCAAGTTCA	360
Phascolarctos cinereus	CTCTTAGGACAGCTTAATTCAAATGTGGACCCCAAGTTCA	332
Pseudochirops corinnae	CTCTTAGGAGAGTTTAATTCAAATGTGGACCCTAAGTTCA	360
Pseudochirops cupreus	CTCTTAGGAGAGTTTAATTCAAATGTGGACCCCAAGTTCA	360
Phalanger gymnotis	CTCTTAGGAGAGCTTAATTCAAATGTGGACCCCAAGTTCA	360
Pseudocheirus occidentalis	CTCTTAGGAGAGTTTAATTCAAATGTGGACCCCAAGTTCA	360
Pseudocheirus peregrinus	CTCTTAGGAGAGTTTAATTCAAATGTGGACCCCAAGTTCA	360
Petauroides volans	CTCTTAGGAGAGTTTAATTCAAATGTGGACCCCAAGTTCA	360
Tarsipes rostratus	CTCTTAGGAGAGTTTAATTCAAATGTGGACCCCAAGTTCG	360
Vombatus ursinus	CTCTTAGGAGAGCTTAATTCAAATGTGGACCCCATATTCA	319

	370 380 390 400	
Petaurus breviceps	GGTTACCATCAAGCAAAGAGCACAGATGGTTGTCAACCGC	398
Acrobates pygmaeus	GGTTAGCATCAAGCAAAGAGTACACATGGTTGTCAACCGC	395
Distoechurus pennatus	GGTTACCATCAAGCAAAGAGCACAAATGGTTGTCAACCGC	390
Dactylopsila trivirgata	GGTTACCATCAAGCAAAGAGCACAAATGGTTGTCAACCGC	399
Macropus eugenii	GGTTACCTTCAAGCAAGGAGCACTAATGGTTGCCAACCGC	400
Macropus fuliginosus	GGTTACCTTCAAGCAAGGAGCACTAATGGTTGCCAACCGC	400
Macropus giganteus	GGTTACCTTCAAGCAAGGAGCACTAATGGTTGCCAACCGC	400
Pseudochirops archeri	TGTTACCATCAAGCAAAGAGCACAAATGGTTGTCAACCGC	400
Phascolarctos cinereus	GGTTACCATCAAGGAAGGAGAACAAATGGTTGTCAACCGC	372
Pseudochirops corinnae	TGTTACCATCAAGCAAAGAGCACAAATGGTTGTCAACCGC	400
Pseudochirops cupreus	TGTTACCATCAAGCAAAGAGCACAAATGGTTGTCAACCGC	400
Phalanger gymnotis	GGTTACCATCAAGGAAAGAGCACAAATGGTTGTCAACCGC	400
Pseudocheirus occidentalis	GGTTACCATCAAGCAAAGAGCACAAATGGTTGTCAACCGC	400
Pseudocheirus peregrinus	GGTTACCATCAAGCAAAGAGCACAAATGGTTGTCAACCGC	400
Petauroides volans	GGTTACCATCAAGCAAAGAGCACAAATGGTTGTCAACCGC	400
Tarsipes rostratus	CGTTATCCTCAAATAAAGAGCACAAATGGTTGTCAGCCGC	400
Vombatus ursinus	GGTTACCATCAAGCAAGGAGCACAAATGGTTGTCAACCGC	359

	410 420 430 440	
Petaurus breviceps	CATCTAGTGGCTACAAAGAAATTCCCTGGAAAAGGGATTT	438
Acrobates pygmaeus	CATCTAGTGGCTACAAAGAAATTCCGTGGAAAAGGGATTT	435
Distoechurus pennatus	CATCTAGTGGCTACAAAGAAATTCCCTGGAAAAGGGATTT	430
Dactylopsila trivirgata	CATCTAGTGGCTACAAAGAAATTCCCTGGAAAAGGGATTT	439
Macropus eugenii	CATCTAGTGGCTACAAATGAAACTCCCTGGAAAAGGGACT	440
Macropus fuliginosus	CATCTAGTGGCTACAAATGAAACTCCTTGGAAAAGGGACT	440
Macropus giganteus	CATCTAGTGGCTACAAATGAAACTCCTTGGAAAAGGGACT	440
Pseudochirops archeri	CATCTAGTGGCTACAAAGAAATTCCCTGGAAAAGGGATTT	440
Phascolarctos cinereus	CATCTAGTGGCTACAAATGAAACTCCCTGGAAATGGATTT	412
Pseudochirops corinnae	CATCTAGTGGCTACAAAGAAATTCCCTGGAAAAGGGATTT	440
Pseudochirops cupreus	CATCTAGTGGCTACAAAGAAATTCCCTGGAAAAGGGATTT	440
Phalanger gymnotis	CATCTAGTGGCTACAAAAAAATTCCCTGGAAAAGGGATTT	440
Pseudocheirus occidentalis	CATCTAGTGGCTACAAAGAAATTCCCTGGAAAAGGGATTT	440
Pseudocheirus peregrinus	CATCTAGTGGCTACAAAGAAATTCCCTGGAAAAGGGATTT	440
Petauroides volans	CATCTAGTGGCTACAAAGAAATTCCCTGGAAAAGGGATTT	440
Tarsipes rostratus	CATCTAGTGGCTACAAAGAAATTCCCTGGAAAAGGGATTT	440
Vombatus ursinus	CATCTAGTGGCTACAAATGAAACTCCCTGGAAATGGATTT	399

	450 460 470 480	
Petaurus breviceps	GGAAGCTATTCTCCAGATTAGAATTTTAAAGGCTCACCCC	478
Acrobates pygmaeus	GGAAGCTGTTCTCCAGATTCGAATTTTAGAGGTTCTCCCC	475
Distoechurus pennatus	GGAAGCTGTTCTCCAGATTAGAATTTTAGAGGCTCGCCCC	470
Dactylopsila trivirgata	AGAAACTATTCTCCAGATTAGAATTTTAGAGGCTCGCCCC	479
Macropus eugenii	GGAAGCTCTTCTCCAGATTCGAATTTTAGAGGCTCGCCCC	480
Macropus fuliginosus	GGAAGCTCTTCTCCAGATTCGAATTTTAGAGGCTCGCCCC	480
Macropus giganteus	GGAAGCTCTTCTCCAGATTCGAATTTTAGAGGCTCGCCCC	480
Pseudochirops archeri	GGAAGCTATTCTCCAGATTAGAATTTTAGAGGCTCGCCCC	480
Phascolarctos cinereus	GGAAGCTATTCTCCATATTAGAATTTTAGAGGCTCACCCC	452
Pseudochirops corinnae	GGAAGCTATTCTCCAGATTAGAATTTTAGAGGCTCGCCCC	480
Pseudochirops cupreus	GGAAGCTATTCTCCAGATTAGAATTTTAGAGGCTCGCCCC	480
Phalanger gymnotis	CGAAGCTATTCTCCAGATTAGAATTTTAGAGGCTCGCCCC	480
Pseudocheirus occidentalis	GGAAGCTATTCCCTGGATTAGAATTTTAGAGGCTAGCCCC	480
Pseudocheirus peregrinus	GGAAGCTATTCCCTGGATTAGAATTTTAGAGGCTAGCCCC	480
Petauroides volans	GGAAGCTATTCTCCAGATTAGAATTTTAGAGGCTCGCCCC	480
Tarsipes rostratus	GGAGGCTATTCTCCAGATTAGAATTTTAGAGGCTCGCCCC	480
Vombatus ursinus	GGAAGCTATTCTCCAGATGAGAATTTTAGAGGCTCGCCCC	439

	490 500 510 520	
Petaurus breviceps	AAGGCTCTGTACATTTGGGCATCTTCCAGCCTTGCTCTCG	518
Acrobates pygmaeus	AAGGCGCGGTACATTTGGGCATCTTGCAGCCTAGCTCTCG	515
Distoechurus pennatus	AGGGCGCTGTATATTTGGGCATCTTCCAGCCTTGCTCTCG	510
Dactylopsila trivirgata	AAGGCTCTGTACATTTGGGCATCTTCCAGCCTTGCTCTCG	519
Macropus eugenii	AAGGCGCTGCACATTTGGACA---TCCAGCCTTGCTGTCG	517
Macropus fuliginosus	AAGGCGCTGCACATTTGGGCA---TCCAGCCTTGCTCTAG	517
Macropus giganteus	AAGGCGCTGCACATTTGGGCA---TCCAGCCTTGCTCTAG	517
Pseudochirops archeri	AAGGCGCTGTACATTTGGGCATCTTCCAGCCTTGCTCTCG	520
Phascolarctos cinereus	AAGGCGCTGTACGTTTGGGCATCTTCCATCCTTGCTCTCG	492
Pseudochirops corinnae	AAGGCGCCGTACATTTGGGCATCTTCCAGCCTTGCTCTCG	520
Pseudochirops cupreus	AAGGCGCTGTACATTTGGGCATCTTCCAGCCTTGCTCTCG	520
Phalanger gymnotis	AAGGCGCTGTACATTTGGGCATCTTCCAGCCTTGCTCTCG	520
Pseudocheirus occidentalis	AAGGCGCTGTACATTTGGGCATCTTCCAGGCTTGCTCTCG	520
Pseudocheirus peregrinus	AAGGCGCTGTACATTTGGGCATCTTCCAGGCTTGCTCTCG	520
Petauroides volans	AAGGCGCTGTACATTTGGGCATCTTCCAGCCTTGCTCTCG	520
Tarsipes rostratus	AAGGCGCTGTACATCTGGGCATCTTCCAGCCTTCCTCTCG	520
Vombatus ursinus	AAGGCGCTGTACGTTTGGGCATCTTCCAGCCTTGCTCTCG	479

	530 540 550 560	
Petaurus breviceps	GGGAAAGAGCATGCCGCGGGATGCGCCAAAGAGCCCGCGC	558
Acrobates pygmaeus	GGGAAAGTGCATGCGGCACGATGCGCCAAAGAGCCGGCGC	555
Distoechurus pennatus	GGGAAAGTGCTTGCGGCGGGATGCGCCAAAGAGCCCGCGC	550
Dactylopsila trivirgata	GGGAAAGAGCATGCCGCGTGATGCGCCTAAGAGCCCGCGC	559
Macropus eugenii	GGCTAAGTGCGTGCGGCGGGATGCGTCAAAGAGCCCGCGC	557
Macropus fuliginosus	GGCTAAGTGCGTGCCGCGGGATGCGTCAAAGAGCCCGCGC	557
Macropus giganteus	GGCTAAGTGCGTGCCGCGGGATGCGTCAAAGAGCCCGCGC	557
Pseudochirops archeri	GGGAAAGAGCATGCCGCGGGATGCGCCAAAGAGCCCGCGC	560
Phascolarctos cinereus	GGGAAAGTGCGTGTCGCGGGATGCGCTAAAGAGCCCGCGG	532
Pseudochirops corinnae	GGGAAAGAGCATGCCGCGGGATGCGCCAAAGAGCCCGCGC	560
Pseudochirops cupreus	GGGAAAGAGCATGCCGCGGGATGCGCCAAAGAGCCCGCGC	560
Phalanger gymnotis	GGGAAAGTGCGTGCCGCGGGATGCGCCAAAGAGGCCGAGC	560
Pseudocheirus occidentalis	GGGAAAGAGCATGCCGCGGGATGCGCCAAAGAGCCGGCGC	560
Pseudocheirus peregrinus	GGGAAAGAGCATGCCGCGGGATGCGCCAAAGAGCCCGCGC	560
Petauroides volans	GGGAAAGAGCATGCCGCGGGATGTGCCAAAGAGTCCGCGC	560
Tarsipes rostratus	GGGAAAGAGCATGCCGCGGGATGCGCTAAAGAGCCCGCAC	560
Vombatus ursinus	GGGAAAGTGCGTGCCGCGGGATGCGCCAAAGAGCCTGCGC	519

	570 580 590 600	
Petaurus breviceps	CCAAGCTAAGAAGCCTCCAAGACGGAAAGCGACAGCAAGC	598
Acrobates pygmaeus	CCAATCTAAGAAGC------------AAGAGGCGGCAAGC	583
Distoechurus pennatus	CCAAGCTAAGAAGCCTCCAAGACGGAAAGAGACAGCAAGC	590
Dactylopsila trivirgata	CCAAGCAAAGAAGCCTCCAAGACGGAAAGAGACAGCAAGC	599
Macropus eugenii	CCAAGCTAAGAAGCCTCCAAGACCGAGAGAGACAGCAGGC	597
Macropus fuliginosus	CCAAGCTAAGAAGCCTCCAAGACCGAGAGAGACAGCAGGC	597
Macropus giganteus	CCAAGCTAAGAAGCCTCCAAGACCGAGAGAGACAGCAGGC	597
Pseudochirops archeri	CCAAGCTAAGAAGCCTCCAAGACGGAAAGAGACAGCAGGA	600
Phascolarctos cinereus	CCAGGCTAAGAAACCTCTAAGA-GGAGAGAGACAGCAATC	571
Pseudochirops corinnae	CCAAGCTAAGAAGCCTCCAAGACGGAAAGAGACAGCAGGA	600
Pseudochirops cupreus	CCAAGCTAAGAAGCCTCCAAGACGGAAAGAGACAGCAGGA	600
Phalanger gymnotis	CCAAGCTAAGAAGCCTCCAAGACG----GAGACAGCAAGC	596
Pseudocheirus occidentalis	TCAAGCTAAGAAGCCTCCAAGACGGAAAGAGACAGCAAGA	600
Pseudocheirus peregrinus	TCAAGCTAAGAAGCCTCCAAGACGGAAAGAGACAGCAAGA	600
Petauroides volans	CCAAGTTAAGAAGCCTCCAAGACGGAAAGAGACAGCAAGA	600
Tarsipes rostratus	CCATCCTAAGAAGCCTCCCAGACGGAAAGAGACAGCAAGC	600
Vombatus ursinus	CCAAGCTAAGAAGCTTCTAAGACGAAGAGAGACAGCAAGC	559

	610 620 630 640	
Petaurus breviceps	GGGTTTAGCTCTGGGTACAGACAGGTAGGATGAGGGAGAA	638
Acrobates pygmaeus	GGGGTTAGCTCTGGGGACCGCCAGGTAGGATGAGGGAGCA	623
Distoechurus pennatus	GGGTTTAGCTCTGGGGACAGACAGGTAGGATGAGGGAGAA	630
Dactylopsila trivirgata	GGGTTTAGCTCTGGGTACAGACAGGTAGGATGAGGGAGAA	639
Macropus eugenii	GGGTTTAGCTCGGGGTACAGACAGGTAGGATGAGGGAGAA	637
Macropus fuliginosus	GGGTTTAGCTCTGGGTACAGACAGGTAGGATGAGGGAGAA	637
Macropus giganteus	GGGTTTAGCTCTGGGTACAGACAGGTAGGATGAGGGAGAA	637
Pseudochirops archeri	GGGCTTAGCTCTGGGTACAGACAGGTAGGACGAGGGAGAA	640
Phascolarctos cinereus	GGGTTTAGCTCTGGGTACAGACAGGTAGGATGGGGGAGAA	611
Pseudochirops corinnae	GGGCTTAGCTCTGGGTACAGACAGGTAGGATGAGGGAGAA	640
Pseudochirops cupreus	GGGCTTAGCCCTGGGTACAGACAGGTAGGATGAGGGAGAA	640
Phalanger gymnotis	GGTTTTAGCTCTGGGTACAGACAGGTAGCATGGGGGAGAA	636
Pseudocheirus occidentalis	GGGTTTAGCTCTGGGTACAGACAGGTAGGATGAGGGAGAA	640
Pseudocheirus peregrinus	GGGTTTAGCTCTGGGTACAGACAGGTAGGATGAGGGAGAA	640
Petauroides volans	GGGTTTAGCTCTGGGTACAGACAGGTAGAAGGAGGGAAAA	640
Tarsipes rostratus	GAGTTTAGTTCTGGGTACACACAGGTAGGACGAGGGAGAA	640
Vombatus ursinus	GGGTTTAGCTCTGGGTACAGACAGGTAGGATGGGGGAGAA	599

	650 660 670 680	
Petaurus breviceps	ATCCTCGCCTGCACGCAGCGTTTGTGCTCTAAAAGGGTAA	678
Acrobates pygmaeus	AGCCTTGCTACACCGGTGCATTAGTGCTCTAA-----CGG	658
Distoechurus pennatus	ATCCTTGCCTACGCGGAGCATTTGTGCTCTAA-AGGGTAA	669
Dactylopsila trivirgata	ATCCTCGACTACACGCAGCGTTTGTGCTTTAAAAGGGTAA	679
Macropus eugenii	ATCCTTGCCTGCGCGCAGCATTTGTGGTCTAAAAGGGTAA	677
Macropus fuliginosus	ATCCTTGCCTGCGCGCAGCATTTGTGGTCTAAAGGGGTAA	677
Macropus giganteus	ATCCTTGCCTGCGCGCAGCATTTGTGGTCTAAAGGGGTAA	677
Pseudochirops archeri	ATCCTTGCCTACACGCAGCATTTGTGCTCTAAGAGGGTAA	680
Phascolarctos cinereus	ATCCTTGCCTACACGCAGCATTTGTGCTCTAAAAGGGTAA	651
Pseudochirops corinnae	ATCCTTGCCTACACGCAGCGTTTGTGCTCTAAGAGGGTAA	680
Pseudochirops cupreus	ATCCTTGCCTACACGCAGCGTTTGTGCTCTAAGAGGGTAA	680
Phalanger gymnotis	ATCCTTGCCTACAC---GCATTTGTGCTCTAAAAGG----	669
Pseudocheirus occidentalis	ATCCTTGCTTACACGCAGCGTTTGTGCTCTAAGAGGGTAA	680
Pseudocheirus peregrinus	ATCCTTGCTTACACGCAGCGTTTGTGCTCTAAGAGGGTAA	680
Petauroides volans	ATCCTTGCCTACACGCAGCGTTTGTGCTCTAAGAGGGTAA	680
Tarsipes rostratus	ATCCTTGCCTACACACAGCATTTGTGCTCTAAAAGGGTAA	680
Vombatus ursinus	ATCCTTGCCTCCATGCAGCATTTGTGCTCTAAAAGGGTAA	639

	690 700 710 720	
Petaurus breviceps	GTAAGTAGCAGGCTGCAGGCTGCTCGCTTTCCCATCGGAT	718
Acrobates pygmaeus	GTAAGTAGCAGGCTGCAGGATGCTCGCTTTCCCAAT-GAT	697
Distoechurus pennatus	GT-ATTAGCAGGCTGCAGGATGCTCGCTTTCCCATCGGAT	708
Dactylopsila trivirgata	GTAAGTAGCAGGCTGCAGGCTGCTTGCTTTCCCATCAGAT	719
Macropus eugenii	GTAAGTAGCAGGCTGCGGGTTGCTCGCTTTCCCATCGGAT	717
Macropus fuliginosus	GTAAGTAGCAGGCTGCGGGTTGCTCGCTTTCCCATCGGAT	717
Macropus giganteus	GTAAGTAGCAGGCTGCGGGTTGCTCGCTTTCCCATCGGAT	717
Pseudochirops archeri	GTAAGTAGCAGGCTGCAGGCTGCTCGCTGTCTCTTCGGAT	720
Phascolarctos cinereus	GTAAGTAGCAGGCTACAGGCTGCTTGCTTTCCAATCGGAT	691
Pseudochirops corinnae	GTAAGTAGCAGGCTGCAGGCTGCTCGCTT--------GAT	712
Pseudochirops cupreus	GTAAGTAGCAGGCTGCAGGCTGCTCGCTTTCTCTTCGGAT	720
Phalanger gymnotis	GTAAGTAGCAGGCTGCAGGCTGCTGCCTTTCCTTTCGGTT	709
Pseudocheirus occidentalis	GTAAGCAGCAGGCTGCAGGCTGCTCGCTTTCCCATCGGAT	720
Pseudocheirus peregrinus	GTAAGCAGCAGGCTGCAGGCTGCTCGCTTTCCCATCGGAT	720
Petauroides volans	GTAAGTAGCAGGCTGCAGGCTGCTCGCTTTCCCATCGGAT	720
Tarsipes rostratus	GTAAGTAGCAAGCTGCAGGCTGCTCGCTTTCCTATCGGAT	720
Vombatus ursinus	GTAAGTAGCAGGCTACAGGCTGCACGCTTTCCCACCGGAT	679

	730 740 750 760	
Petaurus breviceps	CGTTCCCTGCGTGCGTCTGGACCGGCCCGACCGGCCCTTC	758
Acrobates pygmaeus	CGATCCCTGCGTGCGTCTAGACCCGCCCGACCAGCCTCTC	737
Distoechurus pennatus	CGTTCCCTGCGTGCGTCTAGACCCGCCCGACCAGCCTCTC	748
Dactylopsila trivirgata	CTTTCCCTGCGTGCGTCTAGACCCGCCCGACCGGCCCTTC	759
Macropus eugenii	CGTTCCCTGTGTGCGTCTAGACCGGCCCGACCCGCCCCTC	757
Macropus fuliginosus	CGTTCCCTGTGTGCGTCTAGATCGGCCCGACCCGCCCCTC	757
Macropus giganteus	CGTTCCCTGTGTGCGTCTAGATCGGCCCGACCCGCCCCTC	757
Pseudochirops archeri	CGGTCCCGGCGTGCGTCTGGACACGCCCGACCGGCCCCTC	760
Phascolarctos cinereus	CGTTCCGTGCGTGCGTCT--ACCGGCCCGAGCGGCCCCTC	729
Pseudochirops corinnae	CGGTCCCGGCGTGCGTCTAGACACGCCCGACCGGCCCCTC	752
Pseudochirops cupreus	CAGTCCCGGCGTGCGTCTAGACACGCCCGACCGGCCCCTC	760
Phalanger gymnotis	CGTTCCCTGCGTGCCCCTAGACCGGCCCGACCGGCCCCTC	749
Pseudocheirus occidentalis	CGGTCCCTGCGTGCGTCTAGACACGCCCGACCGGCCCCTC	760
Pseudocheirus peregrinus	CGGTCCCTGCGTGCGTCTAGACACGCCCGACCGGTCCCTC	760
Petauroides volans	CGGTCCCTGCGTGCGTCTAGACACGCCCGACCGGCCCCTC	760
Tarsipes rostratus	CGTTCCCCGAGTGCGTCTAGACCCGCCCGACCGGCCTCTC	760
Vombatus ursinus	CGTTACCTGAGTGCGTCTAGACCCGCCCGACTGGTCTTTC	719

	770 780 790 800	
Petaurus breviceps	CAGGCCCTGCCTTAAGCCGGAGGAGCAGGAATCGCTGGGT	798
Acrobates pygmaeus	CAGGACCTGCCTTAAGCGCGGGGAGCAAGAATCGCGGGGT	777
Distoechurus pennatus	CAGGCCCTGCCTTAAGCTGGGGGAGCGGGAATTGCTGGGT	788
Dactylopsila trivirgata	CAGGCCCTGCCTTAAGCCGGAGGAGCAGGAATCGCTGGGT	799
Macropus eugenii	CAGGCTCTGTCTTAAACAGAAGGAACAGGGATCGCCGGGT	797
Macropus fuliginosus	CAGGCTCTGTCTTAAACAGAGGGAACAGGGATCGCCGGGT	797
Macropus giganteus	CAGGCTCTGTCTTAAACAGAGGGAACAGGGATCGCCGGGT	797
Pseudochirops archeri	CAGGCCCTGCCTTAAGCCGGAGAAGCAGAAATCGCTGGGT	800
Phascolarctos cinereus	CAGGCCCAGCCTTCAGGTGGGGGAGCAGGAATCTCTGGGT	769
Pseudochirops corinnae	CAGGCCCTGCCTTAAGCCAGAGAAGCAGGAATCGCTGGGT	792
Pseudochirops cupreus	CAGGCCCTGCCTTAAGCCAGAGAAGCAGGAATCGCTGGGT	800
Phalanger gymnotis	CAGGTCCTGCGTTAAGCAGAGGGAGGAGGGATCACTGGGT	789
Pseudocheirus occidentalis	CAGGCCCTGCTTTAAGCCGGAGTAGCGGGAATCGTTGCGT	800
Pseudocheirus peregrinus	CAGGCCCTGCTTTAAGCCGGAGTAGCGGGAATCGTTGCGT	800
Petauroides volans	CAGGCCCTGCCTTAAGCCGAAGGAGCGAGAATCGCTGGGG	800
Tarsipes rostratus	CA-GCCCTGCTTTAAGCCGGAGGAACAGGAATCGCTGGGT	799
Vombatus ursinus	CAGGCCCAGTCTTAAGCTGAGGGAGCAGGAATCGCTGGGT	759

	810 · 820 · 830 · 840	
Petaurus breviceps	CTTGGCTGTGTTTGCGCCAAGGGAGCAGGCTGTCCTTGGA	838
Acrobates pygmaeus	CTTGGCTGCGTTTGCGCCAGGGGAGCAGGCTGTACTTGGA	817
Distoechurus pennatus	CTTAGCTGCGTTTGCGCCAGGGGAGCAGGCTGTACTTGGA	828
Dactylopsila trivirgata	TTTGGCTGTGTTTGCGCCAGGGGAGCAGGCTGTCCTTGGA	839
Macropus eugenii	CTTGGCTGCATTTGCGCCAGGGGAGTAGGCTGTCCTTGGA	837
Macropus fuliginosus	CTTGGCTGCATTTGCGCCAGGGGAGTAGGCTGTCCTTGGA	837
Macropus giganteus	CTTGGCTGCATTTGCGCCAGGGGAGTAGGCTGTCCTTGGA	837
Pseudochirops archeri	CTTGGCAGCATCTGCGCCAGGGGAGCAGGCTGTCCTTGGA	840
Phascolarctos cinereus	CTTGGCTGCCTTTGGGGCAGTGGAGCAGGCTGTCCTTGGA	809
Pseudochirops corinnae	CTTGGCAGCGTCTGCCCCAGGGGAGCAGGCTGTCCTTGGA	832
Pseudochirops cupreus	CTTGGCAGCGTCTGCGCCAGGGGAGCAGGCTGTCCTTGGA	840
Phalanger gymnotis	CTTGGCTGCATTTGAGCCAGGAGAGCAGGCTGT-------	822
Pseudocheirus occidentalis	CTTGCCAGTGTCTGCGCTAGAGGAGCAGGCTGTCTTTGGA	840
Pseudocheirus peregrinus	CTTGCCAGTGTCTGCGCTAGAGGAGCAGGCTGTCTTTGGA	840
Petauroides volans	CTTGGCAGTGTCTGCGCTAGGGGAGCAGGCTGTCCTTGGA	840
Tarsipes rostratus	CTTGGCAGTGTTTGTGCCAGGGGAGCAGGCTGTCCTTGGA	839
Vombatus ursinus	CTTGGCTGTGTTTGCGCCAGGGGAGCAGGCTGTCCTTGGA	799

	850 · 860 · 870 · 880	
Petaurus breviceps	GGGGACCCACGTTTAGCCCACCTTTGGCTGGGAGGGAAAT	878
Acrobates pygmaeus	GGGGACCCACGTTTAGCCCCGCTTTGGCGGGGAGGGGAAC	857
Distoechurus pennatus	GGGGACCCACGTTTAGCCCACCTTTGGTAGGGAGAGGAAT	868
Dactylopsila trivirgata	GGGGACCCACGTTTAGCTCACCTTTGGCTGGGAGGGAAAT	879
Macropus eugenii	GGGGACCCACGTTTAGCCCACCTTTGTCGGGGAGGGAAAT	877
Macropus fuliginosus	GGGGACCCACGTTTAGCCCACCTTTGTCGGGGAGGGAAAT	877
Macropus giganteus	GGGGACCCACGTTTAGCCCACCTTTGTCGGGGAGGGAAAT	877
Pseudochirops archeri	GGGGACCCACGTTTAGCCCACCTTTGGCTGGGAGGGAACT	880
Phascolarctos cinereus	GGGGACCCACGTTTAACCCACCTTTGGCGGGGAGGGGAAT	849
Pseudochirops corinnae	GGGGACCCACGTTTAGCCCACCTTTGGCTGGGAGGGAACT	872
Pseudochirops cupreus	GGGGACCCACGTTTAGCCCATGTTTGGCTGGGTGGGAACT	880
Phalanger gymnotis	------CCGTGTTTGGTCGACCTTTGGCGGGGAGGGGAAT	856
Pseudocheirus occidentalis	GGGGACCCACGTTTAGCCCACCTTTGGCTGGGAGGGAACT	880
Pseudocheirus peregrinus	GGGGACCCACGTTTAGCCCACCTTTGGCTGGGAGGGAACT	880
Petauroides volans	GGGGACCCACGTTTAGCCCACCTTTGGCTGGGAGGGAACT	880
Tarsipes rostratus	GGGGACCCACATTTAGCCCA-CTTTGGCGGGGAGGGAAGG	878
Vombatus ursinus	AGGGACCCACGTTTAGCCCACCTTTGGCGGGGAGGGGAAT	839

	890 900 910 920	
Petaurus breviceps	ACACACAACCCCTGAACTCCTGCATCCGTCGGTGCGCAGT	918
Acrobates pygmaeus	ACACACAATCCCTGCCCTCCTCCATCCATGCGTGCGAAGG	897
Distoechurus pennatus	ATACACAATCCCTGCCCTCCTCCATCCATCGGTGCGAAGT	908
Dactylopsila trivirgata	ACACACAACCCCTGAACTCCTCCATCCATCAGTGCGCAGT	919
Macropus eugenii	ACACACAA----------CCCCCATCCATCGGTGCGAGAT	907
Macropus fuliginosus	ACACACAA----------CCCCCATCCATCGGTGCGCAGT	907
Macropus giganteus	ACACACAA----------CCCCCATCCATCGGTGCGCAGT	907
Pseudochirops archeri	ACACACAACCTCTACACTCCTCCGTCCATCGGTGCGCAGT	920
Phascolarctos cinereus	ACACATAAACTCTGAACTCCTCCATCCATCGGTGCTCAGT	889
Pseudochirops corinnae	ACACACAACCCCTACACTCCTCCATCCATGGGTGCGCAGT	912
Pseudochirops cupreus	ACACACAACCCCTACACTCCTCCATCCATGGGTGCGCCGT	920
Phalanger gymnotis	ACACACAACCCCAGCGGTCCTCCATCCATCGGTGCGCAGT	896
Pseudocheirus occidentalis	ACACACAACCCCTACACTCCTCCATCCATCGGTGCGCGGT	920
Pseudocheirus peregrinus	ACACACAACCCCTACACTCCTCCATCCATCGGTGCGCGGT	920
Petauroides volans	ACACACAACCCCTACACTCCTCCATCCGTCGGTGTGCAGT	920
Tarsipes rostratus	ACACACAACCCCTGCACTCCTCCATCCATCGGTGCGCAGT	918
Vombatus ursinus	ACACACAAACCTTGAACTCCTCCATCCATCGGTGCTCAGT	879

	930 940	
Petaurus breviceps	ACCTTAATGAGGGTCCATTTGGGGTTGGG	947
Acrobates pygmaeus	ATCTTAGCAAGGGGCCATTTGGGGTCGGG	926
Distoechurus pennatus	ATCTTAGTAAGGGGCTATTTGGGGTTGGG	937
Dactylopsila trivirgata	ATCTTAATAAGGGGCCATTTGGGGTTGGG	948
Macropus eugenii	ATCTCAGTAAGGAACCCTTTGGGGTTAGG	936
Macropus fuliginosus	ATCTCAGTAAGGAACCCTTTGGGGTTACG	936
Macropus giganteus	ATCTCAGTAAGGAACCCTTTGGGGTTAGG	936
Pseudochirops archeri	ACCTTAAGAAGGGGCCATTTGGGGTTGGG	949
Phascolarctos cinereus	ATTTCAGTAAGGGGTCCTTTGGGAATGGG	918
Pseudochirops corinnae	ATCTTAAGAAGGGGCCATTTGGGGTTGGG	941
Pseudochirops cupreus	ATCTGAAGAAGGGGCCGTTTGGGATTGGG	949
Phalanger gymnotis	ATCTCACTAAGGGGCCATTTGGGGTTGGG	925
Pseudocheirus occidentalis	ATCTTAAGAAGGGGCCATTTGGGCTTGGG	949
Pseudocheirus peregrinus	ATCTTAAGAAGGGGCCATTTGGGCTTGGG	949
Petauroides volans	ATCTAAAGAAGGGGCCATTTGGGGTTGGG	949
Tarsipes rostratus	GTCTTCATAAGGGGCCATTTG--------	939
Vombatus ursinus	ATCTCAGTAAGGGGTCCTTTGGGGTTGGG	908

GAR 32020

	10 · 20 · 30 · 40	
Acrobates pygmaeus	ACCTGTCTGGGTTCCAAATCTGTCATCCGTAAAATGAAAG	40
Distoechurus pennatus	ACCTATCTGGGTTCCAAATTTCTCATGTGTAAAATGAAAG	40
Macropus eugenii	GTAAGACT--------------------------------	8
Macropus fuliginosus	GTAAGACT--------------------------------	8
Macropus giganteus	GTAAGACT--------------------------------	8
Pseudochirops archeri	ACCTGTCTGGGTCCCAGTTCTCTCACCTGTAAAATGAAAG	40
Phascolarctos cinereus	ACCTCTCTGGGTCCCAATTCTCTC---------ATGAAAG	31
Pseudochirops corinnae	ACCTGTCTAGGTCCCAGTTCTCTCATCTGTAAAATGAAAG	40
Pseudochirops cupreus	ACCTGTCTGGGTCCCAGTTCTCTCACCTGTAAAATGAAAG	40
Phalanger gymnotis	ACCCATTTGAGTGCCAACTCTCTCATATGTCCAATGAAAG	40
Pseudocheirus occidentalis	GCCTGTCTGGGTTCCAGTTATCTCACCTGTAAAATGAAAG	40
Pseudocheirus peregrinus	GCCTGTCTGGGTTCCAGTTATCTCACCTGTAATGTGAAAG	40
Petauroides volans	ACCTGTCTGGGTTCCAACTCTCTCACCTGTAAAATGAAAG	40
Vombatus ursinus	ACCTGTCTGGGTTCCAATTCTCTCATCTGTAAAATGAAAG	40

	50 · 60 · 70 · 80	
Acrobates pygmaeus	AGTTGGACTTAAGGACTAAGTATTCCTCTAGCTCTAAGGT	80
Distoechurus pennatus	AGCTGGACTTGAGGACTAAGTATTCCTCTAGCTCTAAGGT	80
Macropus eugenii	-------------AAGAAAGTATTCCTCTAGCTCTAAGGT	35
Macropus fuliginosus	-------------AAGAAAGTATTCCTCTAGCTCTAAGGT	35
Macropus giganteus	-------------AAGAAAGTATTCCTCTAGCTCTAAGGT	35
Pseudochirops archeri	AGTTGGACTTGAAAACTAAGTATTCCTCTAGCTCTAAGGT	80
Phascolarctos cinereus	AGGCGGACTTGAGGACTGAGAGTTGCTCTAGCTCTGAGGT	71
Pseudochirops corinnae	AGTTGGACTTGAAAACTAAGTATTTCTCTAGCTCTGAGGT	80
Pseudochirops cupreus	AGTTGGACTTGAAAACTAAGTATTCCTCTAGCTCTAAGGT	80
Phalanger gymnotis	AGTTGGACTTGAGGACTAAGTATTCCTCTAGCTCTAAGGT	80
Pseudocheirus occidentalis	AGTTGGACTTGAAAACTAAGTATTCCTCTAGCTCGAAGGT	80
Pseudocheirus peregrinus	AATTGGACTTGAAAACTAAGTATTCCTCTAGCTCGAAGGT	80
Petauroides volans	AGCTGGACTTGAAAATGAAGTATTCCTCTAGCTCTAAGGT	80
Vombatus ursinus	AGGTGAACTTGAGGACTAAGTATTACTCTGGCTCTAAGGT	80

	90 · 100 · 110 · 120	
Acrobates pygmaeus	T------------CGCTTTTAGTCTTGTGACC--------	100
Distoechurus pennatus	T------------CACTTTTAGTCAGGTGGCC--------	100
Macropus eugenii	TCTATTTTCCTGC--CTTTTAGTCATGTGGCCCTGGAGAA	73
Macropus fuliginosus	TCTATTTTCCCGC--CTTTTAGTCATGTGGCCCTGGAGAA	73
Macropus giganteus	TCTATTTTCCCGC--CTTTTAGTCATGTGGCCCTGGAGAA	73
Pseudochirops archeri	TCTATTTTCCTGCCACTTTTAGTCATGTGGCCCTGGAGAA	120
Phascolarctos cinereus	TCTATTTTTCTGCCACTTTTGGTCGTGTGGCCTTGGAGAA	111
Pseudochirops corinnae	TCTATTTTCCTGCCACTTTTAGTCATGTAGTCCTGGAGAA	120
Pseudochirops cupreus	TCTATTTTCCTGCTACTTTTAGTCATGTGGCCCTGGAGAA	120
Phalanger gymnotis	TCTATTTCCCTGCCACTTTTAGTCATGCGGCCCTGGAGAA	120
Pseudocheirus occidentalis	TCTATTTTCCTGCCACTTTTTGTCATGTGGCCCTGGAGAA	120
Pseudocheirus peregrinus	TCTATTTTCCTGCCACTTTTTGTCATGTGGCCCTGGAGAA	120
Petauroides volans	TCTATTTTCTTGCCACTTTTAGTCATGCAGCCCTGGAGAA	120
Vombatus ursinus	TCTATTTTTCTGCCACTTTTAGTCATGTGACCTTGGAGAA	120

```
                                      130       140       150       160
Acrobates pygmaeus            ----CTTGGTCTCTCTATGCCTCATTGTTTCACATCTCTA 136
Distoechurus pennatus         ----CTTGGTCTCTCTATGCCTCATTGTTTCTCATCTCTA 136
Macropus eugenii              GTCACTTGGTCTCTCTATGCCTCATTTTTTCACATCTCTA 113
Macropus fuliginosus          GTCACTTGGTCTCTCTATGCCTCATTTTTTCACATCTCTA 113
Macropus giganteus            GTCACTTGGTCTCTCTATGCCTCATTTTTTCACATCTCTA 113
Pseudochirops archeri         GCTACTTGGTCTCTCTATGCCTCATTTTTTCACATCTCTA 160
Phascolarctos cinereus        GTCAGTTGGTCTCTCTGTGCCTCA-TTTTTCACATCTCTA 150
Pseudochirops corinnae        GCTACTTGGTCTCTCTATGCCTCATTTTTTCACATCTCTA 160
Pseudochirops cupreus         GCTACTGGGTCTCTTTATGCCTCATTTTTTCACATCTCTA 160
Phalanger gymnotis            GACACTTGGTCTCGCTATGCCTCATTTTTTCACATCTCTA 160
Pseudocheirus occidentalis    GCCACTTGGTCTCTCTATGCCTCATTTTTTCACATCTCTA 160
Pseudocheirus peregrinus      GCCACTTGGTCTCTCTATGCCTCATTTTTTCACATCTCTA 160
Petauroides volans            GCCACTTGATCTCTCTATGCCTCATTTTTTCACATCTCTA 160
Vombatus ursinus              GTCACTTGGTCTCTCTATGCCTCA-TTTTTCACATCTCTA 159
```

```
                                      170       180       190       200
Acrobates pygmaeus            AAATATGGATTGGCTTTACAGCTCTGCAGAATCATATGAA 176
Distoechurus pennatus         AAATAGGGATGATGTTACATGCTCTGCAGAATCAGATGAA 176
Macropus eugenii              AAACAGGGATAATATTACATGCTTTGCAGAATCAGATGAA 153
Macropus fuliginosus          AAACAGGGATAATATTACATGCTTTGCAGAATCAGATGAA 153
Macropus giganteus            AAACAGGGATAATATTACATGCTTTGCAGAATCAGATGAA 153
Pseudochirops archeri         AAATAGGGATAATATTACATGCTCTGCAGAATCAGATGAA 200
Phascolarctos cinereus        AAATAGGGATAAAATTACATGCTCTGCAGAATCAGATAAA 190
Pseudochirops corinnae        AAATAGGGATAATATTACATGCTCTGCAGAATCAGATGAA 200
Pseudochirops cupreus         AAATAGGAATAATAGTACATGCTCTGCAGAATCAGATGAA 200
Phalanger gymnotis            AAATAAGGATAATATTACATGCTCTGCAGGATCAGATGAA 200
Pseudocheirus occidentalis    AAATAGGGATAATATTACATGCTCTGCAGAATCAGATGAA 200
Pseudocheirus peregrinus      AAATAGGGATAATATTACATGCTCTGCAGAATCAGATGAA 200
Petauroides volans            AAATAGGGATAATATTACATGCTCTGCAGAATCAGATGAA 200
Vombatus ursinus              AAATAGGGATAAAATTACACACTCTGCAGAATCCGATGAA 199
```

```
                                      210       220       230       240
Acrobates pygmaeus            ATAAGTGGCTTCAGAAGTGGTAAAGTATTATACAAATGTA 216
Distoechurus pennatus         ATAAGTGGCTTCATAAGTGGTAAAGTATTATACAAATGTA 216
Macropus eugenii              ATGAGTGACTTCATAAGTGGTAAAGCATTATATAAATGCA 193
Macropus fuliginosus          ATAAGTGACTTCATAAGTGGTAAAGTATTATATAAATGTA 193
Macropus giganteus            ATAAGTGACTTCATAAGTGGTAAAGTATTATATAAATGTA 193
Pseudochirops archeri         ATAAGTGACTTCATAACTGGTAAAGTATTATACAAATGTA 240
Phascolarctos cinereus        ATAAGTGACTTCATAAGC------ATATTATACAAATGTA 224
Pseudochirops corinnae        ATAAGTGACTTCATAACTGGTAAAGTATTATACAAATGTA 240
Pseudochirops cupreus         ATAAGTGACTTCATAACTGGTAAAGTATTATACAAATGTA 240
Phalanger gymnotis            ATAAGTGGCTTCATAAGTGGTAAAGTATTATACAAACGTA 240
Pseudocheirus occidentalis    ATAAGTGACTTCATAAGTGGTAAAGTATTATACAAATGTA 240
Pseudocheirus peregrinus      ATAAGTGACTTCATAAGTGGTAAAGTATTATACAAATGTA 240
Petauroides volans            ATAAGTGACTTCATAAGTGGTAAAGTATTATACAAATGTA 240
Vombatus ursinus              ATAAGTGACTTCATAAGTGGTAAAGTATTATACAAATGTA 239
```

```
                              250       260       270       280
Acrobates pygmaeus            CATTTACAGCACTA-GGGAAGACTCACAGTATGCTGTAAA  255
Distoechurus pennatus         GATTTACAACACTA-AGGAAGAGTCATAGTATGTTGTAAA  255
Macropus eugenii              GATTTACAGCGCCAGGGGAAGAGTCACAGTACATTGTAAG  233
Macropus fuliginosus          GATTTACAGCACCAGGGGAAGAGTCACAGTATGTTGTAAA  233
Macropus giganteus            GATTTACAGCACCAGGGGAAGAGTCACAGTATATTGTAAA  233
Pseudochirops archeri         GATTTACAGCACCAGGGGAAGAGTCACAGTATGTTGTAAA  280
Phascolarctos cinereus        GATTTACAGCACCAGGGGAAGAGTCACAGTATGTTGTAAA  264
Pseudochirops corinnae        GATTTACAGCACCAGGAGAAGAGTCACAGTATGTTGTAAA  280
Pseudochirops cupreus         GATTTACAGCACCAGGGGAAGAGTCACAGTATGTTGTAAA  280
Phalanger gymnotis            GTTTTACAGAACCAGGGAAAGAGTCACAGTATGTTGTAAA  280
Pseudocheirus occidentalis    GATTTACAGCACCAGGGGTGGAGTCACAGTATGTTGTAAA  280
Pseudocheirus peregrinus      GATTTACAGCACCAGGGGTGGAGTCACAGTATGTTGTAAA  280
Petauroides volans            GATTTACAGCACCAGGGGAAGAGTCACAGTATGTTGTAAA  280
Vombatus ursinus              GATTTACAGCACCAGGGGAAGAGTCACAGTATGTGGTAAA  279

                              290       300       310       320
Acrobates pygmaeus            CATGGTAATAGGAAACATGCCTTCACAGTT----------  285
Distoechurus pennatus         CATGGTAATAGAAAACATGCCTTCACAGTTCAAGTGTTTC  295
Macropus eugenii              CATGGTAATAGGAAACATGACTTCACAGTTCAAGTGTTTC  273
Macropus fuliginosus          CATGGTAATAGGAAACATGACTTCACAGTTCAAGTGTTTT  273
Macropus giganteus            CATGGTAATAGGAAACATGACTTCACAGTTCAAGTGTTTC  273
Pseudochirops archeri         CATGGTAATAGGAAACATGCCTTCACAGTTCAAGTGTTTC  320
Phascolarctos cinereus        CATGGTAATAGGAAACATGCCTTCACAGTTCAAGTGTTTT  304
Pseudochirops corinnae        CATGGTAATAGGAAACATGCCTTCACAGTTCAAGTGTTTC  320
Pseudochirops cupreus         CATGGTAATAGGAAACATGCCTTCACAGTTCAAGTGTTTT  320
Phalanger gymnotis            CATGGTAATGGGAAACATGCCTTCACAGTCCAAGTGTTTT  320
Pseudocheirus occidentalis    CATGGTAATAGGAAACATGCCTTCACAGTTCAAGTGTTTC  320
Pseudocheirus peregrinus      CATGGTAATAGGAAACATGCCTTCACAGTTCAAATGTTTC  320
Petauroides volans            CATGGTAATAGGAAACATGCCTTCACAGTTCAAGTGTTTC  320
Vombatus ursinus              CATGGTAATAGGAAACATGCCTTCACAGTTCAAGTGTTTT  319

                              330       340       350       360
Acrobates pygmaeus            --GGGGATTGAAATTTTTAAAGAGCATATGTCCTGGCAAA  323
Distoechurus pennatus         TATGGGTTTGAAATTTTTAAAGAGCATATGTCCTGGCAAA  335
Macropus eugenii              GAGGGTTTTGAAATTTTTAAAGAGCATATGTCCTGGCAAA  313
Macropus fuliginosus          GAGGGGTTTGAAATTTTTAAAGAGCATATGTCCTGGCAAA  313
Macropus giganteus            GAGGGGTTTGAAATTTTTAAAGAGCATATGTCCTGGCAAA  313
Pseudochirops archeri         GAGGGGTTTGAAATTTTGAAAGAGCATATGTCCTGGCAAA  360
Phascolarctos cinereus        GAGGGGTTTGAAATTTTTGAAGAGCATATGTCCTGGCAAA  344
Pseudochirops corinnae        GAGGGGTTTGAAATTTTTAAAGAGCATATGTCCTGGCAAA  360
Pseudochirops cupreus         GAGGGGTTTGAAATTTTTAAAGAGCATATGTCCTGGCAAA  360
Phalanger gymnotis            GAGGGGTTTGAAATTTTAAAAGAGCATATGTCCTGGCAAA  360
Pseudocheirus occidentalis    GAGGGGTTTGAAATTTTTAAAGAGCATATGTCCTGGCAAA  360
Pseudocheirus peregrinus      GAGGGGTTTGAAATTTTTAAAGAGCATATGTCCTGGCAAA  360
Petauroides volans            GAGGGGTTTGAAATTTTTAAAGAGCATATGTCCTGGCAAA  360
Vombatus ursinus              GAGGGGTTTGAAATTTTTAAAGAGCATATGTCCTGGCAAT  359
```

	370 380 390 400	
Acrobates pygmaeus	TTAGCATTTTTCATCACATTTGTGTACCCCAAAACATCTT	363
Distoechurus pennatus	TAAGCATTTTTCATCACATTTGTGTATCCAAAAACATCTT	375
Macropus eugenii	GAAGCATTTTTCCTCACATTTGTGTACCCAAAAGCATCTT	353
Macropus fuliginosus	GAAGCATTTTTCCTCACATTTGTGTACCCAAAAGCATCTT	353
Macropus giganteus	GAAGCATTTTTCCTCACATTTGTGTACCCAAAAGCATCTT	353
Pseudochirops archeri	GAAGTATTTTTCATCACATTTGTGTACCCAAAAGCATCTT	400
Phascolarctos cinereus	GAAACATTTTTCATCACATTTGTGTACCCAAAAGCATCTT	384
Pseudochirops corinnae	GAAGTATTTTTCATCACATTTGTGTACCCAAAAGCATCTT	400
Pseudochirops cupreus	GAAGTATTTTTCATCACATTTGTGTACCCAAAAGCATCTT	400
Phalanger gymnotis	GAAGCATTTTTCATCACATTTGTGTACCCAAAAGCATCTT	400
Pseudocheirus occidentalis	GAAGCATTTTTCATCACATTTGTGTACCCAAAAGCATCTT	400
Pseudocheirus peregrinus	GAAGCATTTTTCATCACATTTGTGTACCCAAAAGCATCTT	400
Petauroides volans	GAAGCATTTTTCATCACATTTGTGTACCCAAAAGCATCTT	400
Vombatus ursinus	GAAACATTTTTCATCACATTTGTGTACCCAAAAGCATCTT	399

	410 420 430 440	
Acrobates pygmaeus	TGTAATTCAAGGCTTATCTATACAATGCCAATATAGCTTG	403
Distoechurus pennatus	TGTAATTCAAGGTTTATCTATACAGAGCCAATATAGCCTG	415
Macropus eugenii	TGTAATTCAAGTTTTGTCTACACAATGCCAATATTGTTAG	393
Macropus fuliginosus	TGTAATTCAAGTTTTATCTACACAATGCCAATATTGTTAG	393
Macropus giganteus	TGTAATTCAAGTTTTATCTACACAATGCCAATATTGTTAG	393
Pseudochirops archeri	TGTAATGCAAGTTTTATCTACACAATGCCAATATAGCCTG	440
Phascolarctos cinereus	TGTAATTCAAGTTTTATCTACACAATGCCAAAATAGCTTG	424
Pseudochirops corinnae	TGTAATTCAAGTTTTATCTACACAATGCCAGTAGAGCCTG	440
Pseudochirops cupreus	TGTAATTCAAGTTTTTTCTACACAATGCCAATATAGCCTG	440
Phalanger gymnotis	TGTGATTCAAGTTTTATCTACACAATGCCACTGTAGCCTG	440
Pseudocheirus occidentalis	TGTAATTCAAGTTTTATCTACACAATGCCAATATAGCCTG	440
Pseudocheirus peregrinus	TGTAATTCAAGTTTTATCTACACAGTGCCAATATAGCCTG	440
Petauroides volans	TGTAATTCAAGTTTTATCTCCATAATGCCAATATAGCCTG	440
Vombatus ursinus	TGTAATTTA--TTTTATCTACACAATGCCAAAATAGCTTG	437

	450 460 470 480	
Acrobates pygmaeus	TTGAAGAGTTAAAAAGTCAATCAATTGGCCAGGTATTTGG	443
Distoechurus pennatus	TTGAAGAGTTAAAAAGTCAATCAATTGGCCAGGTATTTGG	455
Macropus eugenii	TTGAAGAGTTAAATAATCAATCAATTGGTCAGGTATTTGG	433
Macropus fuliginosus	TTGAAGAGTTAAATAATCAATCAATTGGTCAGGTATTTGG	433
Macropus giganteus	TTGAAGAGTTAAATAATCAATCAATTGGTCAGGTATTTGG	433
Pseudochirops archeri	TTGAAGAGTTTAAAAATTGATCAATTGGCCAGGTATTTGG	480
Phascolarctos cinereus	TAGAAGAGTTAAAAAGTCGGTCAATTGGCCAGGTATTTAG	464
Pseudochirops corinnae	TTGAAGAGTTTAAAAATTGATCAATTGGCCAGGTATTTGG	480
Pseudochirops cupreus	TTGAAGAGTTTAAAAATTGATCAATTGGCCAGGTATTTGG	480
Phalanger gymnotis	TTCAAGAGTTAAAAAGTCAATCAATTACCTTGGTATTTGT	480
Pseudocheirus occidentalis	TTGAAGAGTTTAAAAGTTGATCAATTGGCCAGATATTTGG	480
Pseudocheirus peregrinus	TTGAAGAGTTTAAAAGTTGATCAATTGGCCAGATATTTGG	480
Petauroides volans	TTGAAGAGTTAAAAAGTTGATCAATTGGCCAGGTATTTGG	480
Vombatus ursinus	TAGAAGAGTTAAAAAGTCCATCAATTTGCCAGGTATTTGG	477

	490 · 500 · 510 · 520	
Acrobates pygmaeus	AGAGCATCAAACAATGATTTGACTGAA-------------	470
Distoechurus pennatus	AGAGCAACACCCAATGATTTGACTGAA-------------	482
Macropus eugenii	AAATTATCACACAATGATTTGACTGAATTCTCCACCCCCA	473
Macropus fuliginosus	AGATTATCACATAATGATTTGACTGAATTCTCCACCCCCA	473
Macropus giganteus	AGATTATCACATAATGATTTGACTGAATTCTCCACCCCCA	473
Pseudochirops archeri	AGAACATCACGCAATGATTTGACTGAATTCCCCACCTCCA	520
Phascolarctos cinereus	AGAACATCACACAATGATTTGACTGAATTCCCTGCCTCCA	504
Pseudochirops corinnae	AGAACATCACGCAATGATTTGACTGAATTCCCCACCTCCA	520
Pseudochirops cupreus	AGAACATCACGCAATGATTTGACTGAATTCCCCACCTCCA	520
Phalanger gymnotis	AGAACATCACACAACAATTAGACTGAATTCTCCACCCCCA	520
Pseudocheirus occidentalis	AGAACATCACACAATCATTTGACTGAAGTCCCCACCTCCA	520
Pseudocheirus peregrinus	AGAACATCACACAATCATTTGACTGAATTCCCCACCTCCA	520
Petauroides volans	AGAACATCACACAGTGATCTGACTGAATTCCCTACCTCCA	520
Vombatus ursinus	AGAACATCACACAATAATTTGACTGAATTCCCTGCCCCCA	517

	530 · 540 · 550 · 560	
Acrobates pygmaeus	-TTCCACTAAATTGACTCTTTATTATTCTTTCTTTCT--C	507
Distoechurus pennatus	-TTCTACTAAATTGACTCTTTCTTTCTTTTTCTTTCT---	518
Macropus eugenii	ATTCCACTAAATTGATTTCCTTCCTTCCTTCCTTCCT--T	511
Macropus fuliginosus	ATTCCACTAAATTAATTTTCTTCCTTCCTTCCTTCCT--T	511
Macropus giganteus	ATTCCACTAAATTAATTTTCTTCCTTCCTTCCTTCTT--T	511
Pseudochirops archeri	GTTCCACTAAATTGACTTTTTTTCTTTCTTTTTCTCT--C	558
Phascolarctos cinereus	GTTCTGCTGAATTGGCTTTTTCTTTCTTTCTTTCTTT--C	542
Pseudochirops corinnae	GTTCCACTAAATTGACTTTTTTTTCTTTCTTTTTCTC--T	558
Pseudochirops cupreus	GTTCCACTAAATTGACTTTTTTTTCTTTCTTTTTCTC--T	558
Phalanger gymnotis	GTTCCACTAAATTGATCCGTCCTTTCTTTCTTTCTTTCTT	560
Pseudocheirus occidentalis	GTTTCACTAAATTGACTTTCTTTCTTTCTTTTTATTT--C	558
Pseudocheirus peregrinus	GTTTCACTAAATTGACTTTCTTTCTTTCTTTTTCTTT--C	558
Petauroides volans	GTTCCACTAAATTGACTTTCTTTCTTTTTCTTTCT-----	555
Vombatus ursinus	ATTCTGCTGAATTGGCTTTTTCTTTCTTTCTTTTTCT--T	555

	570 · 580 · 590 · 600	
Acrobates pygmaeus	TGTCCTTCTTTCCTTTCTTTATCCCACCTTCTTT------	541
Distoechurus pennatus	TGCCCTTCCTTCCTTTCTTTATCCCACCTTCCTT------	552
Macropus eugenii	CTTCCTTCCTTCCTTCCTTCCTTCCTTCCTTCCTTCCTTC	551
Macropus fuliginosus	CTTCCTTCCTTCCTTCCTTCCTTCCTTCCTTCCTTCCTTC	551
Macropus giganteus	CTTCCTTCCTTCCTTCCTTCCTTCCTTCCTTCCTTCCTTC	551
Pseudochirops archeri	TTTCCTTCCTTCCTTCCTTCCTTCCTTCCTTCCTTCCTTT	598
Phascolarctos cinereus	TTTTCTTTCTTTCTTTCTTTCTTTCTTTCTTTCTTTCTTT	582
Pseudochirops corinnae	CTTCCTCCCTTCCTTTCTTCTTTCCTTCCTTCCTTCCTTT	598
Pseudochirops cupreus	CTCCCTTCCTTTCTTCTTTCCTTCCTTCCTTCCTTCCTTT	598
Phalanger gymnotis	TTTTCTTTCTTTCTTTCTTTCTTTGTCTTTCTTTTTTTCT	600
Pseudocheirus occidentalis	TTTCCTCCCTTCCTTTCTTCTTTCTTTCCTTCCTTCCTTT	598
Pseudocheirus peregrinus	TTTCCTCCCTTCCTTTCTTCTTTCTTTCCTTCCTTCCTTT	598
Petauroides volans	----CTTTCTCCCTTCCTTCCTTCCTTCCTTCATTCTTTC	591
Vombatus ursinus	TTTTCTTTCTTTCTTTCTTTCTTTCTTTCTTTCTTTCTTT	595

	610 … 640	
Acrobates pygmaeus	---------------------------------ATTCTCT	548
Distoechurus pennatus	---------------------------------TCTCTCT	559
Macropus eugenii	CTTCCTTTTTCTTCCCTTTTTCCTTTCTCTTCCTCTTTCT	591
Macropus fuliginosus	CTTCCTTTTTCTTCCCTTTTTCCTTTCTCTTCCTCTTTCT	591
Macropus giganteus	CTTCCTTTTTCTTCCCTTTTTCCTTTCTCTTCCTCTTTCT	591
Pseudochirops archeri	CTTTATCCTTGCCTTCCTTCCTCTCTTTCTTCCTTTCTTT	638
Phascolarctos cinereus	CTTTCTTTCTTTCTTCCCCTTTCTTTTTCTTCTTTCCTTT	622
Pseudochirops corinnae	CTTTATCCTTGCCTTCCTTCCTCTCTTTCTTCCTTTCTTT	638
Pseudochirops cupreus	CTTTATCCTTGCCTTCCTTCCTCCCTTTCTTCCTTTCTTT	638
Phalanger gymnotis	CTTTATCCCTGCCTTCCTTC----TTTCCTCTCTTTCTTC	636
Pseudocheirus occidentalis	CTTTATCCTTGCCTTCCTTCCTCTCTTTCTCCCTTTCTTT	638
Pseudocheirus peregrinus	CTTTATCCTTGCCTTCCTTCCTCTCTTTCTCCCTTTCTTT	638
Petauroides volans	CTTTATCCTTGACTTCCTTCCTCTCTTT----CTTCCTTT	627
Vombatus ursinus	CTTTCTTTCTTTCTTCCTCTTTTTCTTTCTTCCTTTCTTT	635

	650 … 680	
Acrobates pygmaeus	TTCTTCCTTTCCTTCATAGAATTTTGCTCTTTCCAATTTC	588
Distoechurus pennatus	TTCTTCCTTTCTTTCATAGTATTTTGTTTTCTCCAATTAC	599
Macropus eugenii	TCCTT--------------TATTTTATTTTCCCCAATTAC	617
Macropus fuliginosus	TTCTTCCTTTATTTGATAGTATTTTATTTTCCCCAATTAC	631
Macropus giganteus	TTCTTCCTTTATTTGATAGTATTTTATTTTCCCCAATTAC	631
Pseudochirops archeri	CTTTTCCTTTCTTTCACAATATTTTTTTTTTCCCAATTAC	678
Phascolarctos cinereus	CTTTTCCTTTCTTTCATAGTATTTTATTTTTCCCAATTAC	662
Pseudochirops corinnae	CTTTTCCTTTCTTTCACAATATTTTTTTTTTCCCAATTAC	678
Pseudochirops cupreus	CTTTTCCTTTCTTTCACAATATTTTTTTTTTCCCAATTAC	678
Phalanger gymnotis	CTTTTCCTTGCTTTCATAGTATTTTATTTTTCCCAGTTAC	676
Pseudocheirus occidentalis	CTTTTCCTTTCTTTCATAATATTTTTTTTTTCCCAATTAC	678
Pseudocheirus peregrinus	CTTTTCCTTTCTTTCATAATATTTTTTTTTTCCCAATTAC	678
Petauroides volans	CTTTTCCTTTCTTTCATAATATTTTTTTTTTCCCAATTAC	667
Vombatus ursinus	CTTTTCCTTTCTTTCATAGTATTTTATTTTCCCCAATTAC	675

	690 … 720	
Acrobates pygmaeus	ATATAAAGACAACCTTAACATTTATTTTAAAATTTTTTGA	628
Distoechurus pennatus	ATATAAAGACAA-CTTAACAGTCATTTAAAAAATTTTTGA	638
Macropus eugenii	ATATAAAGACAATTTTAACATTCATTTAAAAATTTTTTGA	657
Macropus fuliginosus	ATATAAAGACAATTTTAACATTCATTTAAAAATTTTTTGA	671
Macropus giganteus	ATATAAAGACAATTTTAACATTCATTTAAAAATTTTTTGA	671
Pseudochirops archeri	ATATAAAGACAATTTTAACATTCATTTAAAAAAATTTTGA	718
Phascolarctos cinereus	ATGTAAAGACAATTTTAACATTCATTTTTTAAAATTTTGA	702
Pseudochirops corinnae	ATACAAAGACAATTTTAACATTCATTTAAAAAAATTTTGA	718
Pseudochirops cupreus	ATATAAAGACAATTTTAACATTCATTTAAAAAAATTTTGA	718
Phalanger gymnotis	ATGTAAAGACAATTTTAATATTCATTTAAAAAATTTCC--	714
Pseudocheirus occidentalis	ATAT-AAGACAATTTTAACATTCATTTTAAAAAGTTTTGA	717
Pseudocheirus peregrinus	ATAT-AAGACAATTTTAACATTCATTTAAAAAAGTTTTGA	717
Petauroides volans	ATATAAAGACAATTTTAACATTCATTTAAAAAAA-TTTGA	706
Vombatus ursinus	ATGTAAAGACAATTTAAACATTTATTTTTTAAAATTTTGA	715

```
                              730       740       750       760
Acrobates pygmaeus            GTTTCAGATTTTCTTCTTCCCTTTCTTCTCACAACAGAAA 668
Distoechurus pennatus         GTTTCAAATTTTCTTCTTCCCTTTTTCCCCACAACAGAAA 678
Macropus eugenii              GTTTCACATTTTCTCCCTCCCTCCCTCTCAAAGACACAAA 697
Macropus fuliginosus          GTTTCACATTTTCTCCCTTCCTCCCTCTCAAAGACAGAAA 711
Macropus giganteus            GTTTCACATTTTCTCCCTTCCTCCCTCTCAAAGACAGAAA 711
Pseudochirops archeri         GTTTCAAATTTTCTCCTTCCTTCTCTCCCTAAGACAGAAA 758
Phascolarctos cinereus        GTTTCAAATTTTC----TTCCTCCCTCCTTAAGACCGAAA 738
Pseudochirops corinnae        GTTTCAAATTTTCTCCTTCCTTCTCCCCCTAAGACAGAAA 758
Pseudochirops cupreus         GTTTCAAATTTTCTCCTTCCTTCTCTCCCTAAGACAGAAA 758
Phalanger gymnotis            GTTTCAAATTTTCTCCCTTCTTCCTTCCCTAAGACAGAAA 754
Pseudocheirus occidentalis    GTTTCAAATTTTCTCCTTCCCTCTCTCCCTAAAACAGAAA 757
Pseudocheirus peregrinus      GTTTCAAATTTTCTCCTTCCCTCTCTCCCTAAAACAGAAA 757
Petauroides volans            GTTTCAAATTTTCTCCTTCCCTCTCTCCCTAAGACAGAAA 746
Vombatus ursinus              GTTTCAGATTTTCTTCCTTCCTCCCTCCCTAAGACAGAAA 755
```

```
                              770       780       790       800
Acrobates pygmaeus            GCAATTTGATATAGGTTAGATTATGCAATCATGTAAAACA 708
Distoechurus pennatus         GCAATTTGGTATAGGTTAGAATATGCAATCATGTAAAATA 718
Macropus eugenii              GCAATTTGATATAG----------GCAACCATGTAAAACA 727
Macropus fuliginosus          GCAATTTGACATAG----------GCAATCATGTAAAACA 741
Macropus giganteus            GCAATTTGATATAG----------GCAATCATGTAAAACA 741
Pseudochirops archeri         GTAGTTTGATATAGGCTAGATTATGCACTCATGTAAAACA 798
Phascolarctos cinereus        GCAATTTGATACTGGTAAGATTATGAAATCATGTTAAACA 778
Pseudochirops corinnae        GTAATTTGATATACGCTAGATTATGCACTCATGTAAAACA 798
Pseudochirops cupreus         GTAATTTGATATAGGCTAGATTATGCACTCATGAAAAACA 798
Phalanger gymnotis            GCAATTTGATATAGGTTAGGTTATGCAATCATGTAAAACA 794
Pseudocheirus occidentalis    GTAATTTGATATAGGCTAGATTATGCAATCATGTAAAACA 797
Pseudocheirus peregrinus      GTAATTTGATATAGGCTAGATTATGCAATCATGTAAAAGA 797
Petauroides volans            GTAATTTAATAAAGGCTAGATTATGCAATCATGTAAAACA 786
Vombatus ursinus              GCAATTTGATACTGGTTAGATGATGAAATCATGTTAAACA 795
```

```
                              810       820       830       840
Acrobates pygmaeus            TATTTCCATACTAGTGGA-GTACAAAAGAAGAAATAGACT 747
Distoechurus pennatus         TATTTCTGTACTAGTCAA-TTGCGGAAGAAGAAACAAACT 757
Macropus eugenii              TATTTCCATACTAGTCAAGTTGCAAAAGAAGAAATAGGCC 767
Macropus fuliginosus          TATTTCCATACTAGTCAAGTTGCAAAAAAAGAAATAGGCC 781
Macropus giganteus            TATTTCCATACTAGTCAAGCTGCAAAAAAAGAAATAGGCC 781
Pseudochirops archeri         TATTTTCATGCTAGTCAAGTTGCAAAAGAATAAATAGACT 838
Phascolarctos cinereus        TATTTCCATATTAGTCAAGTTACAAAAGAAGAAATAGACC 818
Pseudochirops corinnae        TATTTCCATGCTAGTCAAGTTACAAAAGAATAAATAGACT 838
Pseudochirops cupreus         TATTTCCATGCTAGTCAAGTTGCAAAAGAATAAATAGACT 838
Phalanger gymnotis            CATTTCTATACTAGTCAAGTTGTAAAAGAAGAAATAGACC 834
Pseudocheirus occidentalis    TATTTCCATACTAATCAAGTTGCAAAAGGAGAAATAGACT 837
Pseudocheirus peregrinus      TATTTCCATACTAATCAAGTTGCAAAAAAAGAAATAGACT 837
Petauroides volans            TATTCCCATACTAATCAAGTTGCAAAAGAAGAAAAAGACT 826
Vombatus ursinus              TATTTCCATATTAGTCAAGTTACAAAAGAAGAAATAGACC 835
```

	850 860 870 880	
Acrobates pygmaeus	AAAAGGAAAAC-AAAAAATTAAAGAAAGTGAAAATAGTAT	786
Distoechurus pennatus	AAAAGAAAAAACAAAAAAGTAAAGAAAGTGAAAATAGTAT	797
Macropus eugenii	GAAAGAAAAAA-AAAAAAATAAAGAAAGT-GAAATAGTAT	805
Macropus fuliginosus	AAAAGAAAAAACAAAAAAATAAAGAAAGT-GAAATAGTAT	820
Macropus giganteus	AAAAGAAAAAACAAAAAAATAAAGAAAGT-GAAATAGTAT	820
Pseudochirops archeri	AAAAGAAAAAAACAAAAAATAAAGAAAGTGAAAATAGTAT	878
Phascolarctos cinereus	CAAAAAAAACC-CCACAAAAAAAGAAAGTAAAAATAGTAT	857
Pseudochirops corinnae	AAAAG-AAAAAACAAAAAATAAAGAAAGTGAAAATAGTAT	877
Pseudochirops cupreus	AAAAGAAAAAAACAAAAAATAAAGAAAGTGAAAATAGTAT	878
Phalanger gymnotis	AAAAGAAAAAACAAAAAAATAAAGAAAGTAAAAATAGTAT	874
Pseudocheirus occidentalis	AAAAGAAAAAA--CAAAAATAAAGAAAGTGAAAAGAGTTT	875
Pseudocheirus peregrinus	AAAAGAAAAAA--CAAAAATAAAGAAAGTGAAAAGAGTTT	875
Petauroides volans	AAAAGAAAAAAACCTGAAATAAAGAAAGTAAAAATAGTAT	866
Vombatus ursinus	AAAAAATC----CCACAGAAAAAGAAAG-GAAAATAGTAT	870

	890 900 910 920	
Acrobates pygmaeus	TCTTCAATCTGCATTCAAACTCCATCACTTTTTTTTCTAG	826
Distoechurus pennatus	TCTTCAATCTGCTTTCAGACTCCATCAGTTTTTTCTCTGG	837
Macropus eugenii	AGTTCAATCTGCATTCATACGCCATCAACTTTTCCTCTGG	845
Macropus fuliginosus	AGTTCAATCTGCATTCATACGCCATCAACTTTTCCTCTGG	860
Macropus giganteus	AGTTCAATCTGCATTCATACGCCATCAACTTTTCCTCTGG	860
Pseudochirops archeri	GCTTCAATCTGCATTCAGACTCCATCGGTTTTTTCTCTGG	918
Phascolarctos cinereus	ACTTCAATCTGT-TTCAAACTCCATCAGTTTTTTCTCTGG	896
Pseudochirops corinnae	GCTTCAATCTGCATTCAGACTCCATTGGTTTTTTCTGTGG	917
Pseudochirops cupreus	GCTTCAATCTGCATTCAGACTCCATTGGTTTTTTCTCTGG	918
Phalanger gymnotis	GCTTCAGTCTTCATTCAGACTCCATCAGTTTTTCTTCTGG	914
Pseudocheirus occidentalis	GCTTCAATCTGCACTCAGACTCTATCAGTTTTTTCTCTGC	915
Pseudocheirus peregrinus	GCTTCAATCTGCACTCAGACTCTATCAGTTTTTTCTCTGC	915
Petauroides volans	GCTTCAATCTGCATCCAGACTCCATCAGTTTTTTCTCTGG	906
Vombatus ursinus	GCTTCAATCTGT-TTCAAACTGCATCAGTTTTTTCTCTGG	909

	930 940 950 960	
Acrobates pygmaeus	AGGTAGATAGCATTTTTCATCATGAGTCCTTTGGAATTCT	866
Distoechurus pennatus	AGGTAGATTGCATTTTTCATCATGAGTCCTTTGGAATTCT	877
Macropus eugenii	AGGTTGATAGCATTTTTCATCATGAGTCCTTTGGAATTCT	885
Macropus fuliginosus	AGGTTGATAGCATTTTTCATCATGAGTCCTTTGGAATTCT	900
Macropus giganteus	AGGTTGATAGCATTTTTCATCATGAGTCCTTTGGAATTCT	900
Pseudochirops archeri	AGGTGGATAGCATTTTCTATCATGAGTCTTTTGGAATTAT	958
Phascolarctos cinereus	AGGTGGATAGCATTTGCTATCATGAGTCCTTTGGAATTCT	936
Pseudochirops corinnae	AGGTGGATAGCATTTTCTATCATGAGTCTTTTGGAATTAT	957
Pseudochirops cupreus	AGGTGGATAGCATTTTCTATCATGAGTCTTTTGGAATTAT	958
Phalanger gymnotis	AGGTGGGTAGCATTTTCCATCCCAAATTCCTTGGAATTCT	954
Pseudocheirus occidentalis	AGGTGGATCGCATTTTCTATCATGAGTCCTTCGGAATTAT	955
Pseudocheirus peregrinus	AGGTGGATCGCATTTTCTATCATGAGTCCTTCGGAATTAT	955
Petauroides volans	AGGTGGATAGCATTTTCTGTCATGAGTCCTTTGGAATTAT	946
Vombatus ursinus	AGGTGGATAGCATTTTCCATCATGAGTCCTTTGGAATTCT	949

	970 980 990 1000	
Acrobates pygmaeus	CTTGCTGAGAATAGCTAAGTCACTAACTAATGATCATCAT	906
Distoechurus pennatus	CTGGCTAAGAATAGCTAAGTCACTAAATAATGGTCATCAT	917
Macropus eugenii	CTTGCTGAGAATAGCCAAGTCATTCATAATTGATCATCAT	925
Macropus fuliginosus	CTTGCTGAGAATAGCTAAGTCATTCATAATTGATCATCAT	940
Macropus giganteus	CTTGCTGAGAATAGCTAAGTCATTCATAATTGATCATCAT	940
Pseudochirops archeri	CTTGCTGAGAATAGCTAAATTATTAATAATTGATCATCAT	998
Phascolarctos cinereus	CTTGCTAAGAATAGCTAAGTCATTCATAATTGATCATCAG	976
Pseudochirops corinnae	CTTGCTGAGAATAGCTAAATTATTAATAATTGATCATCAT	997
Pseudochirops cupreus	CTTGCTGAGAATAGCTAAATTATTAATAATTGATCATCAT	998
Phalanger gymnotis	CTTGATGAGAATAGCTAAGTTATTCATAACTGATCAACCT	994
Pseudocheirus occidentalis	CTTGCTGAGAATAGCTAAATCATTAATAATTGATCATCAT	995
Pseudocheirus peregrinus	CTTGCTGAGAATAGCTAAATCATTAATAATTGATCATCAT	995
Petauroides volans	CTTGCCAAGAATAGCTACATCATTAACAATTGTTCATCAT	986
Vombatus ursinus	CTTGCTGAGAATAGCTAAGTCATTCATAATTGATCATCAG	989

	1010 1020 1030 1040	
Acrobates pygmaeus	ACAATGTTGTTGTTACTGTGTACAGTGTTCTCTTGGTTCT	946
Distoechurus pennatus	ACAATGTTGCTGTTACTGTGTACAATGTTCTCTTGGTTCT	957
Macropus eugenii	ACAATGTTGCTGTTACTGTGTACAATGTTCTCTTGGTTTT	965
Macropus fuliginosus	ACAATGTTGCTGTTACTGTGTACAATGTTCTCTTGGTTTT	980
Macropus giganteus	ACAATGTTGCTGTTACTGTGTACAATGTTCTCTTGGTTTT	980
Pseudochirops archeri	ATAGTGTTGCTGTTACTGTGTACAATGTTCTCTTGGTTCT	1038
Phascolarctos cinereus	ACAATGTTGCTATTACTGTGTACAATGTTCTCTTGGTTCT	1016
Pseudochirops corinnae	ATAGTGTTGCTGTTACTGTGTACAATGTTCTCTTGGTTCT	1037
Pseudochirops cupreus	ATAGTGTTGCTGTTACTGTGTACAATGTTCTCTTGGTTCT	1038
Phalanger gymnotis	ACAATGTTGCTGT-----TGTACAATGGTCTCTTGGTTCT	1029
Pseudocheirus occidentalis	ATGATGTTGCTGTTACTGTGTACAATGTTCTCTTGGTTCT	1035
Pseudocheirus peregrinus	ATGATGTTGCTGTTACTGTGTACAATGTTCTCTTGGTTCT	1035
Petauroides volans	ATAATATTGCTATTACTGTGTACAATGTTCTCTTGGTTCT	1026
Vombatus ursinus	ACAATGTTGCTGTTATTGTGTACAATGTTCTCTTGGTTCT	1029

	1050 1060 1070 1080	
Acrobates pygmaeus	GCTCACTTGACTTTTCATCAGTTCATGCAGGTCTTTCCAG	986
Distoechurus pennatus	GCTCACTTCACTTTGCATCAGCTCATGTAAG----TCCAG	993
Macropus eugenii	GCTCACTTCACTTTGTATCAGTTCATGTAAATCTTTCCAG	1005
Macropus fuliginosus	GCTCACTTCACTTTGTATCAGTTCATGTAAATCTTTCCAG	1020
Macropus giganteus	GCTCACTTCACTTTGTATCAGTTCATGTAAATCTTTCCAG	1020
Pseudochirops archeri	GCTCACTTCACTCAGCATCAGTTCATGTAAGTCTTTCCAG	1078
Phascolarctos cinereus	GCTCACTTCACTTTGCATCAGCTCATCTAAGTCTTTCCAG	1056
Pseudochirops corinnae	GCTCATTTCATTCAGCATCAGTTCATGTAAGTCTTTCCAG	1077
Pseudochirops cupreus	GCTCACTTCACTCGGCATCAGTTCATGTAAGTCTTTCCAG	1078
Phalanger gymnotis	GCTCACTTCACTTTGCATCAGTTCATGTAAGTTTTTCCAG	1069
Pseudocheirus occidentalis	GCTCATTTCACTCAGCATCAGTTCATGTAAGTCTTTCCAG	1075
Pseudocheirus peregrinus	GCTCACTTCACTCAGCATCAGTTCATGTAAGTCTTTCCAG	1075
Petauroides volans	GCTCACTTCACTCAGCATCAGTTCATGTAAGTCTTCCCAG	1066
Vombatus ursinus	GCTCACTTCACTTTGCATCAGTTTATGTAAGTCTTTCCAG	1069

	1090 1100 1110 1120	
Acrobates pygmaeus	GTTTTTCTGAAATTAGCCTGCTCATCATTTCTTATGCCAC	1026
Distoechurus pennatus	GTTTTTCTGAAATTAGCCTGCTCATCATTTCTTATACCAT	1033
Macropus eugenii	GTTTTTC---AATCAGCCTGCTTATCATTTCTTATAGCAC	1042
Macropus fuliginosus	GTTTTTCTGAAATCAGCCTGCTTATCATTTCTTATAGCAC	1060
Macropus giganteus	GTTTTTCTGAAATCAGCCTGCTTATCATTTCTTATAGCAC	1060
Pseudochirops archeri	GTTTTTCTGAAGTCCACCTGCTCCTCATTTCTTATACCAC	1118
Phascolarctos cinereus	GTTTTACTGAAATCAGCCTGCTCATCTTTCTTCATAGCAC	1096
Pseudochirops corinnae	GTTTTTCTGAAGTCCACCTGCTCCTCATTTCTTATACCAC	1117
Pseudochirops cupreus	GTTTTTCTGAAGTCCACCTGCTCCTCATTTCTTATACCAC	1118
Phalanger gymnotis	GTTTTTTTGAAATCAGCCTGCTTGTCATTTCTTATAACAC	1109
Pseudocheirus occidentalis	GTTTTTCTGAAGTCCACCTGCTCATCATTTCTTATACCAC	1115
Pseudocheirus peregrinus	GTTTTTCTGAAGTCCACCTGCTCATCATTTCTTATACCAC	1115
Petauroides volans	GTTTCTCTGAAGTCCACCTATTCATCATTTCTTATGCCAC	1106
Vombatus ursinus	GTTATTCTGAAATCAGCCTGCTCATC-TTTCTTATAGCAC	1108

	1130 1140 1150 1160	
Acrobates pygmaeus	AATAGTATTCTACTACATTCATATACCACAACTTATTCAG	1066
Distoechurus pennatus	AATAATATTCCACTACATTCATATACCACAACTTATTCAG	1073
Macropus eugenii	AATAGTATTCCATTATATTCATATATCACAACTCATTCAG	1082
Macropus fuliginosus	AATAGTATTCCGTTATATTCATATATCACAATTCATTCAG	1100
Macropus giganteus	AATAGTATTCCGTTATATTCATATATCACAATTCATTCAG	1100
Pseudochirops archeri	AAAGATATTCCATTATATTCATAGACCACAACTTGTTCAG	1158
Phascolarctos cinereus	AATAGTATTTCATTATATTCATATCCCAGAACTTGTCCAG	1136
Pseudochirops corinnae	AAAGGTATTCCATTATATTCATAGACCACAACTTGTTCAG	1157
Pseudochirops cupreus	AAAGGTATTCCATTATATTCATAGACCACAACTTGTTCAG	1158
Phalanger gymnotis	AATAGTATTCTATTACATTCATATGCCACAACTTGTTCAG	1149
Pseudocheirus occidentalis	AAAGGTATTCCATTACATTCAGAGACCATAACTTGTTCAG	1155
Pseudocheirus peregrinus	AAAGGTATTCCATTACATTCAGAGACCATAACTTGTTCAG	1155
Petauroides volans	AAAGGTATTCCATTACATTCATAAACCACAACTTGTTCAG	1146
Vombatus ursinus	AATAGTATTTCATTATATTCATATACCAGAACTTGTTCAG	1148

	1170 1180	
Acrobates pygmaeus	CCATTTCCCAACTGATGGACATCCCC	1092
Distoechurus pennatus	ACATTCCCCAATTGATGGGTATCCCC	1099
Macropus eugenii	CCATTCCCCAAGTGATAGGCATCCTC	1108
Macropus fuliginosus	CCATTCCCCAAGTGATAGGCATCCCC	1126
Macropus giganteus	CCATTCCCGAAGTGATAGGCATCCCC	1126
Pseudochirops archeri	CCATTCCCCAATTGATGAGCATCC--	1182
Phascolarctos cinereus	TCTTTCTCCAGTTGATGGGCATCCCC	1162
Pseudochirops corinnae	CCATTCCCCAATTGATGATCATCACC	1183
Pseudochirops cupreus	CCATTCCCCAATTGATGAGCATCCCC	1184
Phalanger gymnotis	CCATTTCCCAGTTGATGGGTATCCCC	1175
Pseudocheirus occidentalis	CCATTCCCTAATTGATGGGCATCCCC	1181
Pseudocheirus peregrinus	CCATTCCCTAATTGATGGGCATCCCC	1181
Petauroides volans	CCATTCCCTAATTGATGGGCATCCCC	1172
Vombatus ursinus	CCTTAATCCAATTGATGGGCATCCCC	1174

GAR 41701

Species	1–40	
Petaurus breviceps	GGAAAAAGATACAAGGCAGTTACAAAAATATCTGAGGAAC	40
Acrobates pygmaeus	GGAAAAAAATGCAAAGCAGTTACAAAAATATATGAGGAGC	40
Distoechurus pennatus	GGAAAAAGATGGAACGCAGTTACAAAAATATCTGAGGAGC	40
Dactylopsila trivirgata	GGAAAAAGATTCAAGGCAGTTACAAAACTATCTGAGGAGC	40
Macropus eugenii	GGAAAAAGATGAGAAGCAATTGCAAAAATATCCCAAGAGC	40
Macropus fuliginosus	GGAAAAAGATGCGAAGCAGTTGCAAAAATATCCCAAGTGC	40
Macropus giganteus	GGAAAAAGATGCGAAGCAGTTGCAAAAATATCCCAAGTGC	40
Pseudochirops archeri	GGAACAAGATAGGAGGCAGTTACAAAAATATCTGAGGAGC	40
Phascolarctos cinereus	GGAAAAAGATACAAAGCACTTAC-AAAATATCTGAGGAGA	39
Pseudochirops corinnae	GGAACAAGATAGGAACCAGTTACAAAAATATCTAAGGAGC	40
Pseudochirops cupreus	GGAACAAGATAGGAGCCAGTTACAAAAATATCTAAGGAGC	40
Phalanger gymnotis	GGAAAAAGATGCAAGGCAGTTACAAAAATA--TGAGGAGC	38
Pseudocheirus occidentalis	GGAAAAAGATAGGAGGCAGTTACAAAAATATCTGAGGAGC	40
Pseudocheirus peregrinus	GGAAAAAGATAGGAGGCAGTTACAAAAATATCTGAGGAGC	40
Petauroides volans	GGAAAAAGATCCGAGGCAGTTACAAAAATATCTGAAGAGC	40
Tarsipes rostratus	GGAAAAAGACACAAGGCAATTACAGAAATATCTGAGGAGC	40
Vombatus ursinus	GGAAAAAGATTCAAGGCAGTTACAAAAATATCTGAGGAGA	40

Species	41–80	
Petaurus breviceps	TGTCATTCTGAGATTGGCCATTAGATGGCAGCCAAAGAAT	80
Acrobates pygmaeus	TGTCATTCTTAAAATGGCCAGTAGATGGCAGCCGAAGAAT	80
Distoechurus pennatus	TGTCATTCTGAAATTGGCCAGTAGATGGCAGCCGAAGAAT	80
Dactylopsila trivirgata	TGTCATTCCGAGATTGGCCATTAGATGGCAACCGAAGAAT	80
Macropus eugenii	TGTCATTCTGAGATTGGTCAGTAGATGGCAGCCAAAGAAT	80
Macropus fuliginosus	TGTCATTCTGAGATTGGCCAGTAGATGGCAGCCAAAGAAT	80
Macropus giganteus	TGTCATTCTGAGATTGGCCAGTAGATGGCAGCCAAAGAAT	80
Pseudochirops archeri	TGTCATTCTGAGATTGGCCATTAGATGGCAGGCGAAGAAT	80
Phascolarctos cinereus	TGCCATTCTGAGATTAGCCAGTAGGTGGCAGCCAAGGAAT	79
Pseudochirops corinnae	TGTCATTCTGAGATTGGCCATTAGATGGCAGGCGAAGAAT	80
Pseudochirops cupreus	TGTCATTCTGAGATTGGCCATTAGATGGCAGGCGAAGAAT	80
Phalanger gymnotis	TGTCATTCTGAGATTAGCCAGTAGATGGCAGTCGAAGAAT	78
Pseudocheirus occidentalis	TGTCATTCTAAGATTGGCCATTAGATGGCAGGCGAAGAAT	80
Pseudocheirus peregrinus	TGTCATTCTAAGATTGGCCATTAGATGGCAGGCGAAGAAT	80
Petauroides volans	TGTCATTCTGAAATTGGCCATTAGATGGCAGGCAAAGAAT	80
Tarsipes rostratus	TGTCATTCTGAGATTGGCCATTAGATGGCAGACGAAGAAT	80
Vombatus ursinus	TGTCATTCTGAGATTTGCCAGTAGATGGTAGCCGAAGAAT	80

Species	90 · 100 · 110 · 120	
Petaurus breviceps	GCTGAGATAGTTTAGAGAGGCCTACTTGTTCTGCTTTCCT	120
Acrobates pygmaeus	GCTGAGATCGCTTGGAGAAGCCTCCTTGTTTTGCTTTTCT	120
Distoechurus pennatus	GCTGAGATCGCTTGAAGAAGCCTACTTACTTTGCTTTTCT	120
Dactylopsila trivirgata	GCTGAGATAGCTTAGAGAAGCCCACTTGTTTTGCTTTCCT	120
Macropus eugenii	GCTGTGATCTCTAGGAGGAGGCTACTTGTTTTCTCTTGCT	120
Macropus fuliginosus	GCTGAGATCTCTAGGAGGAGGCTACTTGTTTTCTCTTGCT	120
Macropus giganteus	GCTGAGATCTCTAGGAGGAGGCTACTTGTTTTCTCTTGCT	120
Pseudochirops archeri	GCTGAGATAACTTAGAGAAGCCTACTTGTTTCGCTTTCCT	120
Phascolarctos cinereus	GCTGAGATCATTTGGAGATGGCTGCTTGTTTTGC--TCCT	117
Pseudochirops corinnae	GCTGAGATAGCTTAGAGAAGCCTACTTGTTTCTCTTTCCT	120
Pseudochirops cupreus	GCTGAGATAGCTTAGAGAAGCCTACTTGTTTCTCTTTCCT	120
Phalanger gymnotis	GCTAAGAAGGCTTGGAGAAGGCTATTTGGTCTGCTTTCCT	118
Pseudocheirus occidentalis	GCTGAGATAGCTTAGGGAAGCCTACTTGTTTTGCTTTCCT	120
Pseudocheirus peregrinus	GCTGAGATAGCTTAGGGAAGCCTACTTGTTTTGCTTTCCT	120
Petauroides volans	GCTAAGATACCTTAGAGAAGCCTACTTGTTTGGCTTTACT	120
Tarsipes rostratus	GCTGAGATGGGTTGGAGAAGCCTGCTTCTTTCGCTTTCCC	120
Vombatus ursinus	GCTGAGATTATTTGGAGACGGCTGCTTGTTTTGCTTT-CC	119

Species	130 · 140 · 150 · 160	
Petaurus breviceps	TTATTAGCGGTGGGGATAACAACCTTGCTAGGGGCCTTTG	160
Acrobates pygmaeus	TTATTAGGGGCCTGGATAACAACCTTGCCAAGGGCCTTTG	160
Distoechurus pennatus	TTATTAGGGGCCTGGGTAACAACCTTGCTAAGGGCTTTTG	160
Dactylopsila trivirgata	TTATTAGAGGGTGGGATAACAACCTTGCTAGGGGCCTTTG	160
Macropus eugenii	TTTTTAGGGGTGGGAATAACAACCTTGCTAAGGTTATTTG	160
Macropus fuliginosus	TTTTTAGGGGTGGGAACAACAACCTTGCTAAGGTTGTTTG	160
Macropus giganteus	TTTTTAGGGGTGGGAATAACAACCTTGCTAAGGTTGTTTG	160
Pseudochirops archeri	TTATCAGAGGTAGGGATAACAACTTCCCTAAGGGCCTTTG	160
Phascolarctos cinereus	TTATTAGGGGCCTGGATAACAACCTTGCTCAGGGCCTTTG	157
Pseudochirops corinnae	TTATCAGAGGCAAGGATAACAACTTAGCTAAGGGCCTTTG	160
Pseudochirops cupreus	TTATCAGAGGTAGGGATAACAACTTAACTAAGGGCCTTTG	160
Phalanger gymnotis	TTATTAGGGACCTGGATAACAACCCTGCTAAGGGCCTTTG	158
Pseudocheirus occidentalis	TTATTAGAGGTAGGGATAAGAACTTTGCTAAGGGCCTTTG	160
Pseudocheirus peregrinus	TTATTAGAGGTAGGGATAAGAACTTTGCTAAGGGCCTTTG	160
Petauroides volans	TTATTAGAGGTAGGGATAACAACTTTGCTAAGGGCCTTTG	160
Tarsipes rostratus	TTATTAGTGGTCTGAATAACAACCTTGCTTAAGGCCTCTG	160
Vombatus ursinus	TTATTAGGGACCTGGGTAACAACCTTGCTCAGGGCCTTTG	159

	170 · 180 · 190 · 200	
Petaurus breviceps	AAAGGTAGACCTGCCTACTCTGAGGCTAGCTGGGTAAGTG	200
Acrobates pygmaeus	AAAGGTAGACCTGCCTACTCTGAAGCTAGCAGGGTAAGTG	200
Distoechurus pennatus	AAAGGTAGACCTGCCTACTCTGAGGCTAGCAGGGTAAGTG	200
Dactylopsila trivirgata	AAAGGTAGACCTGCCTACTCTGAGTCTAGCTGGGTAAGTG	200
Macropus eugenii	AAAGGTAGATCTGCCTACTCTGAGGCTAGCAGGGTAAGTG	200
Macropus fuliginosus	AAAGGTAGACCTGCCTACTCTGAGGCTAGCAGGGTAAGTG	200
Macropus giganteus	AAAGGTAGACCTGCCTACTCTGAGGCTAGCAGGGTAAGTG	200
Pseudochirops archeri	AAAGGTAGATCTGCCTACTCTGAGGCTAGCAGGGTAAGTG	200
Phascolarctos cinereus	AATGGTAGACCAGCCTACTCTGAGGCTAGCAGGGTAAGTG	197
Pseudochirops corinnae	AAAGGTAGATCTGCCTACTCTGAGGCTAGCAGGGTAAGTG	200
Pseudochirops cupreus	AAAGGTAGATCTGCCTACTCTGAGGCTAGCAGGGTAAGTG	200
Phalanger gymnotis	AAAGGTAGACCTGCCTACTCTGAAGCTAGCAGGGTAAGTG	198
Pseudocheirus occidentalis	AAAGGTAGATCTGCCTACTCTGCGGCTAGCAGGGTAAGTG	200
Pseudocheirus peregrinus	AAAGGTAGATCTGCCTACTCTGCGGCTAGCAGGGTAAGTG	200
Petauroides volans	AAAGGTAGATTTGCCTACTCGGAGGCTAGCAGGGTAAGTG	200
Tarsipes rostratus	AAAGGTAGACCTGCCTCCTCTGAGGCTAGCAGGGCAAGTG	200
Vombatus ursinus	AATGGTAGACGGGCCTACTCTGTGGCTAGCAGGGTAAGTG	199

	210 · 220 · 230 · 240	
Petaurus breviceps	GAGGAGGTAAACCAGTGACCAGATAAAGAAAACCAGGTTC	240
Acrobates pygmaeus	GAGGAGGTGAGTCAATGACCAGATAAAGAAAGCCATGTTC	240
Distoechurus pennatus	GAGGAGGTGAGCCAGTGACCAGATAAAGAAAGCCATGTTC	240
Dactylopsila trivirgata	GAGGAGGTAAACCAGTGACCAGATAAAGAAAACCACGTTC	240
Macropus eugenii	GAGGAGGTGAGCCAGTGACCAGATAAAGAAAACCATGTTC	240
Macropus fuliginosus	GAGGAGGTGAGCCAGTGACCAGATAAAGAAAACCATGTTC	240
Macropus giganteus	GAGGAGGTGAGCCAGTGACCAGATAAAGAAAACCATGTTC	240
Pseudochirops archeri	GAGGAGGTAAACCAGAGACCAGATAAAGAAAACCATGTTC	240
Phascolarctos cinereus	GAGGAGGTGAACCAGACACGAGATAAAGAAAACGGCATTC	237
Pseudochirops corinnae	GAGGAGGTAAACCAGAGACCAGAAAAAGAAAACCATGTTC	240
Pseudochirops cupreus	GAGGAGGTAAACCAGAGACCAGATAAAGAAAACCATGTTC	240
Phalanger gymnotis	GAGGAGGTGACCCAGTGACCAGATAAAGAAAACCATGTTC	238
Pseudocheirus occidentalis	GAGGAGGTAAACCAAAGACCAGATAAAGAAAACCACGTTC	240
Pseudocheirus peregrinus	GAGGAGGTAAACCAAAGACCAGATAAAGAAAACCACGTTC	240
Petauroides volans	GAGGAGGTAAACCAGAGACCAGATAAAGAAAACCACGTTC	240
Tarsipes rostratus	GAGGAGGTGAACCAGTGACCAGATAAAGAAAACCACGTTC	240
Vombatus ursinus	GAGGAGGTGAACCAGCGACTAGATAAAGAAAGCGGCATTC	239

	250 260 270 280	
Petaurus breviceps	CCACAAGTCAGGGGCAAAAAAAGAAAACACAGCCTTTGAG	280
Acrobates pygmaeus	CCACGATTCAGGGACAAAAAAAGAAAACACAACTTTTGAG	280
Distoechurus pennatus	CCATGATTCAGGGACAAAAAAAGAAAACACAACTTTTGAG	280
Dactylopsila trivirgata	CCACGATTCAGGGACAAAAAAAGAAAACACAGCCTTTGAG	280
Macropus eugenii	CCACGATTCAGGGATAAAAAAAGAAAACACAACCTTTGAG	280
Macropus fuliginosus	CCACGATTCAGGGACAAAAAAAGAAAACACAACTTTTGAG	280
Macropus giganteus	CCACGATTCAGGGACAAAAAAAGAAAACACAACCTTTGAG	280
Pseudochirops archeri	CCACGATTCAGGGACACAAAATGAAAACACAGCCTTTGAG	280
Phascolarctos cinereus	CCACGATTCAGGGACAAAAAAAGAAAACACAACTTTTGAG	277
Pseudochirops corinnae	CCACGGTTCAGGGACACAAAAAGAAAACACAGCTTTTGAA	280
Pseudochirops cupreus	CCACGATTCAGGGACACAAAAAGAAAACACAGCCTTTGAG	280
Phalanger gymnotis	CCACGATTCAGGGACAAAAAAAGAAAACACAACCTTTGAG	278
Pseudocheirus occidentalis	CCACGATTCAGGGACACAAAAAGAAAACACAGCCTTTGAG	280
Pseudocheirus peregrinus	CCACGATTCAGGGACACAAAAAGAAAACACAGCCTTTGAG	280
Petauroides volans	CCACGATTCAGGGACACAAAAAGAAAACACAGTCTTTGAG	280
Tarsipes rostratus	CCATGATTCAGGGACAAAAAACGAAAACACAGCCTTTGAG	280
Vombatus ursinus	CCACGATTCAGGGACAAAAAAAGAAAACACAACCTTTGAG	279

	290 300 310 320	
Petaurus breviceps	ATGAAAATAGGTCAGGGTCTTCCTACAGTGGGAAAAAGGG	320
Acrobates pygmaeus	ATGAAAATAGGTCAGGGTCCTCCTACAGTGGGAAAAAAAT	320
Distoechurus pennatus	ATGAAAATAGGTCAGGGTCCTCCTACAGTGGGAAAAAGGT	320
Dactylopsila trivirgata	ATGAAAATAGGTCAGGGTCTTCCTACAGTGGGAAAAAGGG	320
Macropus eugenii	TTGAAAATAGGTCAGGGTCCTCCTACAGTGGGAAAAAAGG	320
Macropus fuliginosus	TTGAAAATAGGTCAGGGTCCTCCTACAGTGGGAAAAAAGG	320
Macropus giganteus	TTGAAAATAGGTCAGGGTCCTCCTACAGTGGGAAAAAAGG	320
Pseudochirops archeri	ATGAAAATAGGTCAGGGTCTTCCTACAGTGGGAAAAAGGG	320
Phascolarctos cinereus	ATGAAAATAGGTCAGGGTCCTCCTACAGTGGGAAAAAGAA	317
Pseudochirops corinnae	ATGAAAATAGGTCAGGGTCTTCCTACAGTGGGAAAAAGGG	320
Pseudochirops cupreus	ATGAAAATAGGTCAGGGTCTTCCTACAGTGGGAAAAAGGG	320
Phalanger gymnotis	ACGAAAATAGGTCAGGGTCCTCCTACAGTGGGAAAAAAGG	318
Pseudocheirus occidentalis	ATGAAAATAGGTCAGGGTCTTCCGACAGTGGGAAAAAGGG	320
Pseudocheirus peregrinus	ATGAAAATAGGTCAGGGTCTTCCGACAGTGGGAAAAAGGG	320
Petauroides volans	ATGAAAATAGGTCAGGGTCTTCTGACAGTGGGAAAAAGGG	320
Tarsipes rostratus	ATGAAAATAGGTCAGGGTCTTTCAACAGTGGGAAAAAGGG	320
Vombatus ursinus	ATGAAAATAGGTCAGGTTCCTCCTACAGTGGGAAAAAGGG	319

	330 340 350 360	
Petaurus breviceps	GAGGGGGAGGAGAATCCACAGGAAGAGATGGATTTGCCTC	360
Acrobates pygmaeus	GGTAAGGGGGAGAATCCACAGGAAGAGATGGATTTGCCTC	360
Distoechurus pennatus	GGTAAGGGGGAGAATCCACAGGAAGAGATGGATTTGCCTT	360
Dactylopsila trivirgata	GAGGGGGAGGAGAATCCACAGGAAGAGATGGATTTGCCTC	360
Macropus eugenii	GAGGGGGGAGAGAATCCACAGGAAGACATGGATTTGCCTC	360
Macropus fuliginosus	GAGGGGGGAGAGAATCCACAGGAAGACATGGATTTGCCTC	360
Macropus giganteus	GAGGGGGGAGAGAATCCACAGGAAGACATGGATTTGCCTC	360
Pseudochirops archeri	GGAGGGGAGGAGAATCCACAGGAAGAGATGGATTTGCCTC	360
Phascolarctos cinereus	AGGGGGAGGAAGAATACACAGGAAGAGTTGGATTTGCCTC	357
Pseudochirops corinnae	GGAAGGGAGGAGAACCCACAGGAAGAGATGGATTTGCCTC	360
Pseudochirops cupreus	GGAGGGGAGGAGAACCCACAGGAAGAGATGGATTTGCCTC	360
Phalanger gymnotis	---GGGGTTAAGAATCCACAGGAAGAGATGGATTTGCCTT	355
Pseudocheirus occidentalis	GGAGGGGAGGAGAACCCACAGGAAGAGATGGATTTGCCTC	360
Pseudocheirus peregrinus	GGAGGGGAGGAGAACCCACAGGAAGAGATGGATTTGCCTC	360
Petauroides volans	GGAGGGGAGGAGAACCCACAGGAAGAGATGGATTTGCCTC	360
Tarsipes rostratus	GAGGGGGAGGACAATCTACAGGAAGAGATGGATTTGCCTC	360
Vombatus ursinus	AGGGGGAGGAAGAATACACAGGAAGAGATGGATTTGCCTC	359

	370 380 390 400	
Petaurus breviceps	CCTTATCCATTTGGACGAGGGAAAACAACTTCCAAGAACT	400
Acrobates pygmaeus	CCTTATCCATTTGGATGAGGGAAAACAACTTCCAAGAATT	400
Distoechurus pennatus	CCTTATCCATTTGGATGAGGGAAAACAACTTCCAAGAATT	400
Dactylopsila trivirgata	CCTTATCCATTTGGATGAGAGAAAACAACTTCCAAGAAGT	400
Macropus eugenii	CTTTATCCATTTGGATGAGGGAAAACAACTTCCAAGAACT	400
Macropus fuliginosus	CTTTATCCATTTGGATGAGGGAAAACAACTTCCAAGAACT	400
Macropus giganteus	CTTTATCCATTTGGATGAGGGAAAACAACTTCCAAGAACT	400
Pseudochirops archeri	CCTTATCCATTTGGATGAGGGAAAACAACTTCCAAGAACT	400
Phascolarctos cinereus	TCTTATCCATTTGGATGAGGGAAAACAACTTCCAAGAACT	397
Pseudochirops corinnae	CCTTATCCATTTGGATGAGGGAAAACAACTTCCAAGAACT	400
Pseudochirops cupreus	CCTTATCCATTTGGATGAGGGAAAACAACTTCCAAGAACT	400
Phalanger gymnotis	CCTTATCCATTTGGATGAGGGAAAACAATTTCCAAGAACT	395
Pseudocheirus occidentalis	CCTTATCCATTTGGATGAGGGAAAGCAACTTCCAAGAACT	400
Pseudocheirus peregrinus	CCTTATCCATTTGGATGAGGGAAAGCAACTTCCAAGAACT	400
Petauroides volans	CCTTATCCATTTGGATGAGGGGAAAGAACTTCCAAGAACT	400
Tarsipes rostratus	CCTTATCCATTTGGATGAGGGAAAACAACTTCCAAGAACT	400
Vombatus ursinus	CCTTATCCATTTGGATGAAGGAAAACAACTTCCAAGAACT	399

	410 420 430 440	
Petaurus breviceps	GCTGCAAACAGAAACACATGTCCCCTCATCTGGGGTGCAG	440
Acrobates pygmaeus	GCTGCAAACAGAAACACATGTCCCCTCATCTGGGGTGCAG	440
Distoechurus pennatus	GCTGCAAACAGAAACACATGTCCCCTCATCTGGGGTGCAG	440
Dactylopsila trivirgata	GCTGCAAACAGAAACACATGTCCCCTCATCTGGGGTGCAG	440
Macropus eugenii	GCTGCAAACAGAAACACATGTCCCCTCATCTGGGGTGCAG	440
Macropus fuliginosus	GCTGCAAACAGAAACACATGTCCCCTCATCTGGGGTGCAG	440
Macropus giganteus	GCTGCAAACAGAAACACATGTCCCCTCATCTGGGGTGCAG	440
Pseudochirops archeri	GCTGCAAACAGAAACACATGTCCCCTCATCTGGGGTGCAG	440
Phascolarctos cinereus	GCTGCAAACAGAAACACATGTAACCTCATCTGGGGTGCAG	437
Pseudochirops corinnae	GCTGCAAACAGAAACACATGTCCCCTCATCTGGGGTGCAG	440
Pseudochirops cupreus	GCTGCAAACAGAAACACATGTCGCCTCATCTGGGGTGCAG	440
Phalanger gymnotis	GCTGCAAACAGAAACACATGTCACCTCATCTGGGGTGCAG	435
Pseudocheirus occidentalis	GTTGCAAACAGAAACACATGTCCTCTCATCTGGGGTGCAG	440
Pseudocheirus peregrinus	GTTGGAAACAGAAACACATGTCCTCTCATCTGGGGTGCAG	440
Petauroides volans	GTTGCAAACAGAAACACATGTCCCCTCATCTGGGGTGCAG	440
Tarsipes rostratus	GCTGCAAACAGAAACACATGTCCCCTCATCTGGGCTGCAG	440
Vombatus ursinus	GCTG--------------------CTCATCTGGGGTGCAG	419

	450 460 470 480	
Petaurus breviceps	AAGCTGACCATTTCTGAACATTACAATAGTAACTGTCTAT	480
Acrobates pygmaeus	AAGCTGACCATTTCTGAACATTACAATAGTGACTGTCTAT	480
Distoechurus pennatus	AAGCTGACCATTTCTGAACATTACAATAGTAACTGTCTAT	480
Dactylopsila trivirgata	AAGCTGACCATTTCTGAACATTACAATAGTAACTGTCTAG	480
Macropus eugenii	AAGCTGACCCTTTCTGTACACTACAATAGTAACTGTCTAT	480
Macropus fuliginosus	AAGCTGACCCTTTCTGTACATTACAATAGTAACTGTCTAT	480
Macropus giganteus	AAGCTGACCCTTTCTGTACATTACAATAGTAACTGTCTAT	480
Pseudochirops archeri	AAGCTGACCATTTCTGAACATTACAATAGTAACTGTCTAT	480
Phascolarctos cinereus	AAGCTGACCATTTCTGTACATTACAATAGTAACTGGCTTT	477
Pseudochirops corinnae	AAGCTGACCATTTCTGAACATTACAATAGTAACTGTCTAT	480
Pseudochirops cupreus	AAGCTGACCATTTCTGAACATTACAATAGTAACTGTCTAT	480
Phalanger gymnotis	AAGCTGACCATTTCTGTACATTATAATAGTAACTATCTAT	475
Pseudocheirus occidentalis	AAGCTGACCATTTCTGAATATTACAATAGCAACTGTTTAT	480
Pseudocheirus peregrinus	AAGCTGACCATTTCTGAATATTACAATAGCAACTGTTTAT	480
Petauroides volans	AAGCTGACCACTTCTGAGCATTACAATAGTAACTGTCTAT	480
Tarsipes rostratus	AAGCTGACCATTTCTGAACATTATAATAGTAACTATCCAT	480
Vombatus ursinus	AAGCTGACCATTTCTGTACATTACAATAGTAACTGGCTTT	459

	490 500 510 520	
Petaurus breviceps	CAGGACAACAATCCTAACTCATATTTATATGGTACTTTAT	520
Acrobates pygmaeus	CAGGACAACAATCCTAACTCATGTTTATGTGGCACTTTAA	520
Distoechurus pennatus	CAGGACGACAATACTAACTCATGTTTATATGGCACTTTAT	520
Dactylopsila trivirgata	CAGGACAACAATCCTAACTTATATTTATATGGCACTTTAT	520
Macropus eugenii	AAGGACAGCAATCCTAACTCATGTTTATATGGCACTTTAT	520
Macropus fuliginosus	AAGGACAGCAATCCTAACTTGTGTTTATATGGCACTTTAT	520
Macropus giganteus	AAGGACAGCAATCCTAACTTGTGTTTATATGGCACTTTAT	520
Pseudochirops archeri	CAGGACAACGATCCTAACTCATATTTATATGGCACTTTAT	520
Phascolarctos cinereus	TAGGACAGCACTCTTAACTCATATTTATATGGTGCTTCAT	517
Pseudochirops corinnae	CAGGACACCGATCCTAACTCATATTTATATGGCACTTTAT	520
Pseudochirops cupreus	CAGGACAACGATCCTAACTCACATTTATATGGCACTTTAT	520
Phalanger gymnotis	CAGGACAGCAATCCTAACTCATATTTATATGGCACTTTAT	515
Pseudocheirus occidentalis	CAGGACAATAATCCTAACTCATATTTATATGGCACTTTAT	520
Pseudocheirus peregrinus	CAGGACAATAATCCTAACTCATATTTATATGGCACTTTAT	520
Petauroides volans	CGGGACAACAACCCTAACTCATATTTATATGGCCCTTTAT	520
Tarsipes rostratus	CAGGACAACAATCCTAGCTCATGTTTATATGGCACTTTGT	520
Vombatus ursinus	TAGGACAGCACTCTTAACTCGTATTTATATGGTGCTTTAT	499

	530 540 550 560	
Petaurus breviceps	AGCTGGCAAAATGCTTTCCCCCCAATAGCCTTGTGAGATT	560
Acrobates pygmaeus	AGCTGGCAAAATGCTTTCCTCCCAATAGCCTTGTGAGATT	560
Distoechurus pennatus	AGCTGGCAAAGTGCTTTCCTCCCAATAGCCTTGTGAGATT	560
Dactylopsila trivirgata	AGCTGGCAAAGTGCTTTCCTCCCAATAGCCTTGTAAGATG	560
Macropus eugenii	AGTTGGCAAAGTGCTTTCCTCCCAATAGCCTTGTGAGATT	560
Macropus fuliginosus	AGCTGGCAAAGTGCTTTCCTCCCAATAGCCTTGTGAGATT	560
Macropus giganteus	AGCTGGCAAAGTGCTTTCCTCCCAATAGCCTTGTGAGATT	560
Pseudochirops archeri	AGCTGGCAAAGTGCTTTCCTGCCAATAGCCTCGTGAGGTT	560
Phascolarctos cinereus	AACTGGCAGAATGCTTTTCTCCCAATAGCCTTGTGAGATT	557
Pseudochirops corinnae	AGCTGGCAAAGTGCTTTCCTGCCAATAGGCTCGTGAGGTT	560
Pseudochirops cupreus	AGCTGGCAAAGTGCTTTCCTGCCAATAGGCTCGTGAGGTT	560
Phalanger gymnotis	AGCTGGCACAGTGCTTTCCTCCCAATAGCCTTGTGAGATT	555
Pseudocheirus occidentalis	AGCTGGCAAAGTGCTTTCCTCCCAATAGCTTTGTGAGGTT	560
Pseudocheirus peregrinus	AGCTGGCAAAGTGCTTTCCTCCCAATAGCTTTGTGAGGTT	560
Petauroides volans	AGCTAGCAAAGCGCTCTCCTCCTAATAGCCTTGTGAGGTT	560
Tarsipes rostratus	AG---------------CCTCCCAATAGCTTTGTGAGATT	545
Vombatus ursinus	AGCTGGCCAAATGCTTTTCTCCCAATAGCCTTGTGAGATT	539

```
                                     570       580       590       600
Petaurus breviceps             ATTGTTATCAATGCTAGGCTAACCTTGAGGCCTTCAGGAA 600
Acrobates pygmaeus             ATTGTTATCAATGCTAGGCTAACCTTGAGGCCTGCAGGAA 600
Distoechurus pennatus          ACTGTTATCAATGCTAGGCTAACCTTGAGGCCTGCAGGAA 600
Dactylopsila trivirgata        ATTGTTATCAATGCTAGGTTAACCTTGAGGCCTGCAGGGA 600
Macropus eugenii               ATTGTTATCAATGCTAGGCTAACCTTGAGGCCTGCAGGAA 600
Macropus fuliginosus           ATTGTTATCAATGCTAGGCTAACCTTGAGGCCTGCAGGAA 600
Macropus giganteus             ATTGTTATCAATGCTAGGCTAACCTTGAGGCCTGCAGGAA 600
Pseudochirops archeri          ATTGTTATCCACGCTAGGCTAACCTTGAGGCCGGCAGGAA 600
Phascolarctos cinereus         ACTGTTATCAATGCAAGGCTAACCTTGAGGCCTGCAGAAA 597
Pseudochirops corinnae         ATTGTTATCCACGAGAGGCTAACCTTGAGGCCGGCAGGAA 600
Pseudochirops cupreus          ATTGTTATCCACGCTAGGCTAACCTTGAGGCCGGCAGGAA 600
Phalanger gymnotis             ATTGTTATCAATGCTAGGCTAACCTTGAGGCCTGCAGGAA 595
Pseudocheirus occidentalis     ATTGTTATCAGTGCTAGGCTAACCTTGAGGCCTGCAGAAA 600
Pseudocheirus peregrinus       ATTGTTATCAGTGCTAGGCTAACCTTGAGGTCTGCAGAAA 600
Petauroides volans             ATCGTCATCAGTGCTAGGCTAACCTTGAGGCCCGAAGGAA 600
Tarsipes rostratus             ATTGTTATCAATGCTAAGCTAACCTTGAGGCCTGCAAGAA 585
Vombatus ursinus               ATTATTATCAATGCAAGGCTAACCTTGAGTCCTTCAGGAA 579
```

```
                                     610       620       630       640
Petaurus breviceps             AAGTGTTCTCAGAAACAATGAGCTGAGATTTCATTATAAT 640
Acrobates pygmaeus             AAGTGTTCTCAGAAACAAAGAGCTGAGATTTCATTATAAT 640
Distoechurus pennatus          AAGTATTCTCAGAAACAAAGAGCTGAGATTTTATTATAAT 640
Dactylopsila trivirgata        AAGTGTTCTCAGAAACAATGAGCAGAGATTTCATTATACT 640
Macropus eugenii               CAGTGTTTTCAGAAACAATGAGCTAAGATTTCATTATAAT 640
Macropus fuliginosus           CAGTGTTTTCAGAAACAATGAGCTAAGATTTCATTATAAT 640
Macropus giganteus             CAGTGTTTTCAGAAACAATGAGCTAAGATTTCATTATAAT 640
Pseudochirops archeri          AAGTGTTCTCAGAAACAACGAGCTGAGGTTGCATTACAAC 640
Phascolarctos cinereus         CAGTGTTCTCAGAAACAATGATCCGAGATTTCATTATAAT 637
Pseudochirops corinnae         AAGTGTTCTCAGAAACAATGAGCTGAGGTTGCATGAAAAT 640
Pseudochirops cupreus          AAGTGTTCTCAGAAACTATGAGCTGAGGTTGCATTACAAT 640
Phalanger gymnotis             CAGTGTTCACAAAAACAATGAGCTGAGATTTCATTATAAT 635
Pseudocheirus occidentalis     AAGTGTTCTCAGAAACAATGAGCTGAG--TTCATTATAAT 638
Pseudocheirus peregrinus       AAGTGTTCTCAGAAACAATGAGCTGAG--TTCATTATAAT 638
Petauroides volans             AAGTGTTCTCAGAAACAATGAGCTGAGATTTCATCATAAT 640
Tarsipes rostratus             AAGTGTTCTCAGAAACAATCAGCTGAGATTTCATTATAAT 625
Vombatus ursinus               CAGTGTTCTCAGAAACAATGATCGGAGATTTCATTATAAT 619
```

	650 660 670 680	
Petaurus breviceps	GAGGTGCCTCCAACTTCTGATAACTAACACCCAGCCACCC	680
Acrobates pygmaeus	GAGGTTCCTCCAACTTCTGATAACTAACACACCCCCACCC	680
Distoechurus pennatus	AAGGTCCCTCCAACCTCTGATAACTAACACACCCCCACCC	680
Dactylopsila trivirgata	GAGGTGCCTCCAACTTCTGATAACTAACACCCCCCCCCCC	680
Macropus eugenii	GAGATCCCTTCAACTTCTGATAACTAACACTTATCCTCCC	680
Macropus fuliginosus	GAGATCCCTTCAACTTCTGATAACTAACACTTATCCTCCC	680
Macropus giganteus	GAGATCCCTTCAACTTCTGATAACTAACACTTATCCTCCC	680
Pseudochirops archeri	GAGGTGCCTCCAACTTCTGATAACTAACACCCACCCCACC	680
Phascolarctos cinereus	GAGGTTCCTCCAACTTCTGGTAACTAACACGT-CCCTCCC	676
Pseudochirops corinnae	GAGGTGCCTTCAACTTCTGATAACTAACACCCACCCCACC	680
Pseudochirops cupreus	GAGGTGCCTCCAACTTCTGATAACTAACACCCACCCCACG	680
Phalanger gymnotis	GAGGTCCCTTCAACTTCTGGTAACTAACA--------CCC	667
Pseudocheirus occidentalis	GAGGTGCCTCCAACTTCTGATAAGTAAC-CCCCCCCTCCT	677
Pseudocheirus peregrinus	GAGGTGCCTCCAACTTCTGATAAGTAAC-CCCCCCCTCCC	677
Petauroides volans	GAGGTGCCTCCAACTTCTGATAACTAACACCCACCCCACC	680
Tarsipes rostratus	GAGGTGCCTCCAACTTCTGGTAACTAACACCACCCCACCC	665
Vombatus ursinus	GAGGTCCCTCCAACTTCTGGTAACTAACACATCCCTCCCC	659

	690 700 710 720	
Petaurus breviceps	CCACCCCTGGTTGAATGGAGTCCAAGAGAATCCTCCTTAG	720
Acrobates pygmaeus	TCACCCCTGGTTGAATGGAGTCCAAGAGAACCCTCCTTAG	720
Distoechurus pennatus	TCACCCCTGGTTGAATAGAGTCCAAGAGAACCCTCCTTAG	720
Dactylopsila trivirgata	CCACCCCCGCCC---CGGCCTTTGAATGAGTCCTCCTTAG	717
Macropus eugenii	TCACCCCAAGTTGAATGGTGTCCAAGAGAACCCTCCTTAG	720
Macropus fuliginosus	TCACCCCAAGTTGAATGGTGTCCAAGAGAACCCTCCTTAG	720
Macropus giganteus	TCACCCCAAGTTGAATGGTGTCCAAGAGAACCCTCCTTAG	720
Pseudochirops archeri	CAGCCTTTGAATGAATGGAGTCCAGGAGAACCTTCCTTAG	720
Phascolarctos cinereus	CACCCCCCAGTTGAATGGAGTCCAAGAGAGCCCTCCTTAG	716
Pseudochirops corinnae	CAGCCTTTGAATGAACTGAGTCCAGGAGAACCTTCCTTAG	720
Pseudochirops cupreus	AAGCCTTTGAATGAATGGAATCCAGGAGAACCTTCCTTAG	720
Phalanger gymnotis	CCACTCCCAGTTGAATGGAGTCCAAGAGAACCCTCCTTAG	707
Pseudocheirus occidentalis	CAGCCTTTGAATGAGTGGAGTCCAGGATAACCCTCCTTAG	717
Pseudocheirus peregrinus	CAGCCTTTGAATGAGTGGAGTCCAGGATAACCCTCCTTAG	717
Petauroides volans	CAGCCCTTGAGTGAATGGAGTCCAGGAGAACCCTCCTTAG	720
Tarsipes rostratus	CAACCCCTGGTTGAATGGAGTCCAAGAAAACCGTCCTTAT	705
Vombatus ursinus	ATCCCCCCACTTGAATGGAGTCCAAGAGAGCCCTCCTTAG	699

730 740 750 760

Petaurus breviceps	ACTGAATTCTGCATTATACTATGTTGTCTTGTAGTAAAGC	760
Acrobates pygmaeus	ACTGAACTCTGCATTAGACTATGCTGTATTATAGTAAAGC	760
Distoechurus pennatus	ACTGAACTCTGCATTATACTATGTTGTATTATAGTAAAGC	760
Dactylopsila trivirgata	CCTGAATTCTGCATTATACTATGTTGCCTTGTAGTAAAGC	757
Macropus eugenii	ACTGAATTCTGCATTATACTATGTCATGTTATGGTAAAAC	760
Macropus fuliginosus	ACTGAATTCTGCATTATACTATGTCATGTTATGGTAAAAC	760
Macropus giganteus	ACTGAATTCTGCATTATACTATGTCATGTTATGGTAAAAC	760
Pseudochirops archeri	ACTGAATTCTGCATTATACCATGTTGTCTTGGAGTAAAGC	760
Phascolarctos cinereus	ACTGAATTCTGCGTTACACTATGTCATGTTATAGTAAAGC	756
Pseudochirops corinnae	ACTGAATTCTGCATTATACCATGTTGTCTTGTAGTAAAGC	760
Pseudochirops cupreus	ACTGAATTCTGCATTATACCATGTTGTCTTGTAGTAAAGC	760
Phalanger gymnotis	ACTGAATTCTGCATTATACTATGTCATGTTATAGTAAAGC	747
Pseudocheirus occidentalis	ACTGAATTCTGCATTATACTACGCTGTCTTGTAGTAAAGC	757
Pseudocheirus peregrinus	ACTGAATTCTGCATTATACTACGCTGTCTTGTAGTAAAGC	757
Petauroides volans	CCTGAATTCTGCATTCTACTGCATAGTCTTGTAGTAAAGC	760
Tarsipes rostratus	ACTGAATTCTGCATTACACTATGTTGTCTTGTAGTAAAGC	745
Vombatus ursinus	ACTGAATTCTGCATTATACTATGTCATGTTATAGTAAAGC	739

770 780 790 800

Petaurus breviceps	TCTGGTGTGTTATTTATTTGTTTTGCTTATGGATCTGGCC	800
Acrobates pygmaeus	TCTGGTGTCTCGTTAATATGTTTTGCTTTTGGATCTGGCC	800
Distoechurus pennatus	TCTGGTGTCTCGTTAATTTGTTTTGCTTGTGGATCTGGCC	800
Dactylopsila trivirgata	TCTGGTGTCTCATTTATTTGTTTTGCTTATGGATCTGATC	797
Macropus eugenii	TCTGGTGTCTCATTAATTTGTTTTGTTTATGGATCTGGCT	800
Macropus fuliginosus	TCTGGTGTCTCATTAATTTGTTTTGTTTATGGATCTGGCT	800
Macropus giganteus	TCTGGTGTCTCATTAATTTGTTTTGTTTATGGATCTGGCT	800
Pseudochirops archeri	TCTGGTGTCTCCTTTATTTGTTTTGCTTATGGATCTGGCC	800
Phascolarctos cinereus	TCTGGTGTCTCATTTATTTGTGTTGCTCATGGATCTGGCC	796
Pseudochirops corinnae	TCTGGTGTCTCCTTTATTTGTTTTGCTTATGGATCTGGCC	800
Pseudochirops cupreus	TCTGGTGTCTCCTTTATTTGTTTTGCTTATGGATCTGGCC	800
Phalanger gymnotis	TCTGGTGTCTCGTTTATTTGTTTTGCTTATGGATCTGGTC	787
Pseudocheirus occidentalis	TCTGGTGTCTCATTTATTCATTTTGCTTATTGATCTGGCC	797
Pseudocheirus peregrinus	TCTGGTGTCTCATTTATTCATTTTGCTTATTGATCTGGCC	797
Petauroides volans	TCTGGCGTCTCGTTTATTTGTTTTGCTTATGGATCTGGCC	800
Tarsipes rostratus	TCTGGTGTCTCTTTTACTTGTTTTGCTTATGGATCTGGCT	785
Vombatus ursinus	TCTGGTGTCTCGTTTATTTGTGTCGCTCATGGATCTGGCC	779

	810 / 820 / 830	
Petaurus breviceps	TTACCTCATTAGAACAGCACAGCCTGAGTTGGCC	834
Acrobates pygmaeus	TTACCTCATTAGAATAGCTCAGCCTGGATTGGCC	834
Distoechurus pennatus	TTACCTCATTAGAATAGCACAGCCTGGGTTGGCC	834
Dactylopsila trivirgata	TTACCTCATTAGAACAGCACAGCCTGAGTTGGCC	831
Macropus eugenii	TTACCTCATTAGAATAGCACAGTCTGGGATGGCC	834
Macropus fuliginosus	TTACCTCATTAGAATAGCACAGTCTGGGATGGCC	834
Macropus giganteus	TTACCTCATTAGAATAGCACAGTCTGGGATGGCC	834
Pseudochirops archeri	TTACC-----------ACACAGCCTGAGTTGGCC	823
Phascolarctos cinereus	TTACCTCATTAGAATAGCACAGCTTGGGTTAGCC	830
Pseudochirops corinnae	TTACCTCATTAGAATAGCACAGCCTGAGTTGGCC	834
Pseudochirops cupreus	TTACCTCATTAGAATAGCACAGCCTGAGTTG---	831
Phalanger gymnotis	TTACTTCATTAGAATAGCACAGCCTGGGTTGGCC	821
Pseudocheirus occidentalis	TTACCTCATTAGAATAGCACAGCCTGAGTTGGCC	831
Pseudocheirus peregrinus	TTACCTCATTAGAATAGCACAGCCTGAGTTGGCC	831
Petauroides volans	TTACCTCATTAGAACAGCACAGCCTGAGTTGGCC	834
Tarsipes rostratus	CTTCCTCATTAGAATAGCACAGCCTGAGTTGGCC	819
Vombatus ursinus	TCACCTCATTAGAATAGCACGGCTTGGATTAGCC	813

GAR 51182

	10 20 30 40	
Petaurus breviceps	GAAAAAGCTGATTTGTCCATTTATACAGATTGTAAAGAGG	40
Acrobates pygmaeus	AAAAAAGATGATTTGTCCAGTCACACACATTGTAAAGGGC	40
Distoechurus pennatus	AAAAAAGATGATTTGTCCAGTCACACATGTTGTAAAGGGG	40
Dactylopsila trivirgata	AAAAAAGGTGATTTGTCCAATCACACAGATTGTACAGGGG	40
Macropus eugenii	AAAAAAAGTGATTTGTTCAGTCACACAGATTATAAAGGGG	40
Macropus fuliginosus	AAAAAAAGTGATTTGTTCAGTCACACAGATTATAAAGGGG	40
Macropus giganteus	AAAAAAAGTGATTTGTTCAGTCACACAGATTATAAAGGTG	40
Pseudochirops archeri	AAAAAAGATGGTTTGTCCAGTCACACAGATTTTAAAGGGG	40
Phascolarctos cinereus	AAAAAAAGTGATTTTTCCAGTCACACAGATTGTAAAGGGG	40
Pseudochirops corinnae	AAAAAAGATGGTTTGTCCAGTCACACAGATTTTAAAGGGG	40
Pseudochirops cupreus	AAAAAAGATGGTTTGTCCAGTCACACAGATTTTAAAGGGG	40
Phalanger gymnotis	AAAAACAGTGATTTGTCCAGTCACACCGATTGTAAAGGGG	40
Pseudocheirus occidentalis	AAAAAAGGTGATTTGTCCAGTCACACAGATTTTAAAGGGG	40
Pseudocheirus peregrinus	AAAAAAGGTGATTTGTCCAGTCACACAGATTTTAAAGGGG	40
Petauroides volans	AAAAAAGGTGATTTGTCCAGTCACACAGATTTTAAAGGGG	40
Vombatus ursinus	AAAAAAAGTGATTTTTCCAGTTACACAGATTGTAAAGGGG	40

	50 60 70 80	
Petaurus breviceps	AGTTTAGTCTAAGATTAAGCCATTTGGCTTCAGTACATTG	80
Acrobates pygmaeus	AATTTAGACTGAGATTAAGCCATTTGGCTTCAGTACATTG	80
Distoechurus pennatus	AATTTAGAGTGAGATTAAGCCATTTGGCTTCAGTACATTG	80
Dactylopsila trivirgata	AGTTTAGTCTGAGATTAAGCCATTTGGCTTCGGTACGTTG	80
Macropus eugenii	AGTTTAGTCTGAGATTAAGCCATTTGGCTTCAGTACATTG	80
Macropus fuliginosus	AGTTTAGTCTGAGATTAAGCCATTTGGCTTCAGTACATTG	80
Macropus giganteus	AGTTTAGTCTGAGATTAAGCCATTTGGCTTCAGTACATTG	80
Pseudochirops archeri	AGTTTAGTCTGAGATTAAGCCATTTGGCTTCAGTACATTG	80
Phascolarctos cinereus	AGTTTAGTCTGAGATTAAGCCATCTGGCTTCAGTACATTG	80
Pseudochirops corinnae	AGTTTAGTCTGAGATTAAGCCATTTGGCTTCAGTACATTG	80
Pseudochirops cupreus	AGTTTAGTCTGAGATTAAGCCATTTGGCTTCAGTACATTG	80
Phalanger gymnotis	AGTTTAGTCTGAGATTAAGCCATTTGGCTTCAGTACATTG	80
Pseudocheirus occidentalis	AGTTTAGTCTGAGATTAAGCCATTTGGCTTCAGTACATTG	80
Pseudocheirus peregrinus	AGTTTAGTCTGAGATTAAGCCATTTGGCTTCAGTACATTG	80
Petauroides volans	AGTTTAGTCTGAGATTAAGCCATTTGGCTTCAGTACATTG	80
Vombatus ursinus	AGTTGAGTCTGAGATTAAGCCATCTGGCTTCAGTACATTG	80

90 100 110 120

Petaurus breviceps	TCATGTTGAGGCTGCACTACTGGTGAAAGTGGAGCGATTT	120
Acrobates pygmaeus	TCATGTTGAGGCTGCAGTACTGGTGAAAGTGAAGCTATTT	120
Distoechurus pennatus	TCATGTTGAGGCTACACTACTAGTGAAAGTGAAGCGATTT	120
Dactylopsila trivirgata	TCATGTTGAGGCTGCACTACTGGTGAAAGTGGAGCGATTT	120
Macropus eugenii	TCATATTGAGGCTGCACTACTGGTGAAAGTGGAGTGATTT	120
Macropus fuliginosus	TCATGTTGAGGCTGCACTACTGGTGGAAGTGGAGCGATTT	120
Macropus giganteus	TCATGTTGAGGCTGCACTACTGGTGGAAGTGGAGCGATTT	120
Pseudochirops archeri	TCATGTTGAGGCTGCACTACTGGTGAAAGTGGAGCGATTT	120
Phascolarctos cinereus	TCATGTTGAGGCTGCACTACTGGTGAAAGTGGAATGATTT	120
Pseudochirops corinnae	TCATGTTGAGGCTGTACTACTGGTGAAAGTGGAGCGATTT	120
Pseudochirops cupreus	TCATGTTGAGGCTGCACGACTGGTGAAAGTGGAGCGATTT	120
Phalanger gymnotis	TCATGTTGAGGCTGTACTACTGGTGAAAGTGGAGCGATTT	120
Pseudocheirus occidentalis	TCATGTTGAGGCTTCACTACTGGTGAAAGTGGAGCGATTT	120
Pseudocheirus peregrinus	TCATGTTGAGGCTGCACTACTGGTGAAAGTGGAGCGATTT	120
Petauroides volans	TCATGTTGAGGCTGCACTACTGGTGAAAGCGGAGCGATTT	120
Vombatus ursinus	TCATGTTGAGGCTGCACTACTGGTGAAAGTGGAATGATTT	120

130 140 150 160

Petaurus breviceps	TCTCATAATGAAATCATATTTATTGCAAAGATCCTCAAGG	160
Acrobates pygmaeus	TCTCATAATGAAGTCATATTTATTGCAAAGATCCTCAGGG	160
Distoechurus pennatus	TCTCATAATGAAGTCATATTTATTGCAAAAATCCTCAGGG	160
Dactylopsila trivirgata	TCTCATAATGAAGTCATATTTATTGCAAAGATCCTCAAGG	160
Macropus eugenii	TCTCATAGTGAAGTCATATTTATTGCAAAGATCCTCAAGG	160
Macropus fuliginosus	TCTCATAGTGAAGTCATATTTATTGCAAAGATCCTCAAGG	160
Macropus giganteus	TCTTATAGTGAAGTCATATTTATTGCAAAGATCCTCAAGG	160
Pseudochirops archeri	TCTTATAATGAAGTCATATTTATTGCAAAGATCCTCAAGG	160
Phascolarctos cinereus	TCTCATAATGAAGTCATATTTATTGCAAAGATCCTCAAGG	160
Pseudochirops corinnae	TCTCATAACGAAGTCATATTTATTGCAAAGATCCTCAAGG	160
Pseudochirops cupreus	TCTCATAATGAAGTCATATTTATTGCAAAGATCCTCAAGG	160
Phalanger gymnotis	TCTCATAATGAAGTCATATTTATTGCAAAGATCCTCAAGG	160
Pseudocheirus occidentalis	TCTCATAATGAAGTCATATTTATTGCAAAGATCCTCAAGG	160
Pseudocheirus peregrinus	TCTCATAATGAAGTCATATTTATTGCAAAGATCCTCAAGG	160
Petauroides volans	TCTCATAATGAAGTCATATTTATTGCAAAGATCCTCAAGG	160
Vombatus ursinus	TCTCATAATGAAGTCATATTTATTGCAAAGATCCTCAAGG	160

170 180 190 200

Petaurus breviceps	CTACTTAATTGTAAAAATAATTACATATGTCTGAGAAGAA	200
Acrobates pygmaeus	CTACTTAATTGTAAAAATAATTACATATGTCTGAGAAGAA	200
Distoechurus pennatus	CGACTTAATTGTAAAAATAATTACATATGTCTGAGAAGAA	200
Dactylopsila trivirgata	CTACTTAATTGTAAAAATAATTACATATGTCTGAGAAGAA	200
Macropus eugenii	CTACTTAATTGTAAAAATAATTACATATGTCTGAGAAGAA	200
Macropus fuliginosus	CTACTTAATTGTAAAAATAATTACATATGTTGGAGAAGAA	200
Macropus giganteus	CTACTTAATTGTAAAAATAATTACATATGTCGGAGAAGAA	200
Pseudochirops archeri	CTACTTAATTGTAAGAATAATTACATATGTCTGAGAAGAA	200
Phascolarctos cinereus	CTACTTAATTGTAAAAATAATTACATATGTCTGAGAAGAA	200
Pseudochirops corinnae	CTACTTAATTGTAAAAATAATTACATATGTCTGAGAAGAA	200
Pseudochirops cupreus	CTACTTAATTGTAAAAATAATTACATATGTCTGAGAAGAA	200
Phalanger gymnotis	CTACTTAATTGTAAAAATAATTACATATGTCTGAGAAGAA	200
Pseudocheirus occidentalis	CTACTTAATTGTAAAAATAATTACATATGTCTGAGAAGAA	200
Pseudocheirus peregrinus	CTACTTAATTGTAAAAATAATTACATATGTCTGAGAAGAA	200
Petauroides volans	CTACTTAATTGTAAAAATAATTACATATGTCTGAGAAGAA	200
Vombatus ursinus	CTACTTAATTGTAAAAATAATTACATATGTCTGAGAAGAA	200

	210 220 230 240	
Petaurus breviceps	CTGGGAGATCAAGGATTTCTTTCCAAAAATCCCATGATGC	240
Acrobates pygmaeus	CTGGGAGATCAAGGATTTCTTTCCAAAAATCCCATGATGC	240
Distoechurus pennatus	CTGGGAGATCAAGGATTTCTTTCCAAAAATCCCATGATGC	240
Dactylopsila trivirgata	CTGGGAGATCAAGGATTTCTTTCCAAAAATCCCATGATGC	240
Macropus eugenii	CTGGGAGATCAAGAATTTCTTTCCAAAAATCCCATGATGC	240
Macropus fuliginosus	CTGGGAGATCAAGAATTTCTTTCCAAAAATCCCATGATGC	240
Macropus giganteus	CTGGGAGATCAAGAATTTCTTTCCAAAAATCCCATGATGC	240
Pseudochirops archeri	CTGGGAGATCAAGGATTTCTTTCCAAAAATCCCATGATGC	240
Phascolarctos cinereus	CTGGGAGATCAAGGATTTCTTTCCAAAAATCCCATGATGC	240
Pseudochirops corinnae	CTGGGAGATCAAGGATTTCTTTCCAAAAATCCCATGATGC	240
Pseudochirops cupreus	CTGGGAGATCAAGGATTTCTTTCCAAAAATCCCATGATGC	240
Phalanger gymnotis	CTGGGAGATCAAGGATTTCTTCCCAAAAATCCCATGATGC	240
Pseudocheirus occidentalis	CTGGGAGATCAAGGATTTCTTTCCAAAAATCCCATGATGC	240
Pseudocheirus peregrinus	CTGGGAGATCAAGGATTTCTTTCCAAAAATCCCATGATGC	240
Petauroides volans	CTGGGAGATCAAGGATTTCTTTCCAAAAATCCCATGATGC	240
Vombatus ursinus	CTGGGAGATCAAGAATTTCTTTCCAAAAATCCCATGATGC	240

	250 260 270 280	
Petaurus breviceps	TCTTAGATTGCAAATTGTGAAGAAG-GAAAAAAAACAGCT	279
Acrobates pygmaeus	TCTTAGATTGCAAATTTTATGAAGAAGAAAAAAAAATGGCT	280
Distoechurus pennatus	TCTTAGATTGCAAATTATGAAGAAGAAAAAAAAAATGGCT	280
Dactylopsila trivirgata	TCTTAGATTGCAAATTGTGGAGAAGAAAAAAAAAACGGCT	280
Macropus eugenii	TCTTGGATTGCAAATTGTGAAGAAGAAAAAAAAAACAGCT	280
Macropus fuliginosus	TCTTAGATTGCAAATTGTGAAGAAGGAAAAAAAACCAGCT	280
Macropus giganteus	TCTTAGATTGCAAATTGTGAAGAAGGAAAAAAAACCAGCT	280
Pseudochirops archeri	TCTTAGATCACAAATTGTGAAGAAGAAAAAAAAAATGGCT	280
Phascolarctos cinereus	TCTTAGATTGCAAATTGTGAAGAAGAAGAAAAAACCAGCT	280
Pseudochirops corinnae	TCTTAGATTGCAAATTGTGAAGAAGAAAAAAAAAATGGCT	280
Pseudochirops cupreus	TCTTAGATTGCAAATTGTGAAGAAGAAAAAAAAAATGGCT	280
Phalanger gymnotis	TCTTAAATTGCAAATTGTGAAAAGAAGGGGAAAAGCAGCT	280
Pseudocheirus occidentalis	TCTTAAATTGCGAATAGTGAAGACG-AAAAAAAAATGGCT	279
Pseudocheirus peregrinus	TCTTAGATTGCGAATAGTGAAGATG-AAAAAAAAATGGCT	279
Petauroides volans	TCTTAGATTGCAAATTGTGAAGAAGAAAAATAA---GGGT	277
Vombatus ursinus	TCTTAGATTGCAAATTGTGAAGAAGAAGAAGAAACCAGCT	280

	290 300 310 320	
Petaurus breviceps	CTAGGTTACAGAACACCAGGCCTCCAAAAGGGAGAAGCTA	319
Acrobates pygmaeus	CTAGGTTACAGAACACTAGGCCTCCAAAAGGGAGAAACCA	320
Distoechurus pennatus	CTAGGTTACAGAACACCAGGCCTCCAAAAGGGAGAAGCCA	320
Dactylopsila trivirgata	CTAGGTTACAGAACATCAGGCCTCCAAAAGGGGGAAGCTA	320
Macropus eugenii	CTAGGTTACAGAATACCAGGCCTCCAAAAGGGAGAAGATA	320
Macropus fuliginosus	CTAGGTTACAGAACACCAGGCCTCCAAAAGGGAGAAGCTA	320
Macropus giganteus	CTAGGTTACAGAACACCAGGCCTCCAAAAGGGAGAAGCTA	320
Pseudochirops archeri	CTAGGTTACAGAACACCAGGCCTCCAAAAGGAAGAAGCTA	320
Phascolarctos cinereus	CTAGGTTATAGAACACCAGGCCTCCAGAAGGGAGAAGCTG	320
Pseudochirops corinnae	CTAGGTTACAGAACACCAGGCCTCCAAAAGGAAGAAGCTA	320
Pseudochirops cupreus	CTAAGTTACAGAACACCAGGCCTCCAAAAGGAAGAAGCTA	320
Phalanger gymnotis	CTAGGTTACAGAACACCAGGCCTCCAAAAGGGAGAAGCT-	319
Pseudocheirus occidentalis	CTAGGTTACAGAACACCAGGCCTCCAAAAGGAAGAAGCTA	319
Pseudocheirus peregrinus	CTAGGTTACAGAACACCAGGCCCCCAAAAGGAAGAAGCTA	319
Petauroides volans	CTAGGTTACAGAACACCAAGCCTCCAAAAAGAAGAAACTA	317
Vombatus ursinus	CTAGGTTACAGAACACCAGGCCTCCAAAAGGGAGAAGCTA	320

	330 340 350 360	
Petaurus breviceps	TCTAATCCAAAACATATCTAGCCAAGAATCTTCTCTGTAC	359
Acrobates pygmaeus	TCTCATCCAAGA------TGGCCAAGAATCTTCTCTA---	351
Distoechurus pennatus	TCTCATCCAAAATATACCTGGCCAAGAATCTTCTCTG---	357
Dactylopsila trivirgata	TCTAATCCAAAACATACCTAGCCAAGAATCTTCTCTGTAC	360
Macropus eugenii	TCTAATCCAAAACATACCTGATGAAAAA---TCTCTACAC	357
Macropus fuliginosus	TCTAATCCAAAACATACCTGATGAAAAA---CCTCTACAC	357
Macropus giganteus	TCTAATCCAAAACATACCTGATGAAAAA---CCTCTACAC	357
Pseudochirops archeri	TCTAATCCAAAACATACCTAGCCAAGAATCTTCTCTATAC	360
Phascolarctos cinereus	TCTAATCCAAAATATACCTGACCAAGAATCTTCTCTATAC	360
Pseudochirops corinnae	TCTAATCCAAAACATACCTAGCCAAGAATCTTCTCTATAC	360
Pseudochirops cupreus	TCTAATCCAAAACATACCTAGCCAAGAATCTTCTCTATAC	360
Phalanger gymnotis	--CAATCCAAAACATACCTGACCAAGAATCGGCTCTGTAC	357
Pseudocheirus occidentalis	TCTAATCCAAAACATACTTAACCAAGAATCTTCTCTGTAC	359
Pseudocheirus peregrinus	TCTAATCCAAAACATACTTAGCCAAGAATCTTCTCTGTAC	359
Petauroides volans	TCTAATCCAAAAGATACTTAGCCAAAATTCTTCTCTATAC	357
Vombatus ursinus	TCTAATCCAAAATATACCTGACCAAGAATCTTCTCTACAC	360

	370 380 390 400	
Petaurus breviceps	TGGCCCCTACACGTGTTTCTTCAACTTCCCTGTTCTGTTT	399
Acrobates pygmaeus	----CCCTACAAGTGTTCCTTCAACTTCCCCATTCTGTTT	387
Distoechurus pennatus	TGGCCCCTACAAGTGTTCCTTCAACTTCCCCCTTCCGGTT	397
Dactylopsila trivirgata	TGGCCCCTACAAGTGTTCCTTCAACTTCCCCATTCTGTTT	400
Macropus eugenii	TGGTCCCTAGAAGAGTTCCTTCAACTTTCCCGTTCTGTTT	397
Macropus fuliginosus	TGGTCCCTAGAAGAGTTCCTTCAACTTTCCCGTTCTGTTT	397
Macropus giganteus	TGGTCCCTAGAAGAGTTCCTTCAACTTTCCCGTTCTGTTT	397
Pseudochirops archeri	TGGCCCCTACAAGTGTTCCTTCAACTTCCCCATTCTGTGT	400
Phascolarctos cinereus	TGGCCCCTACAAGTGTTCCTTCAACTTTCCCATTTTGCTT	400
Pseudochirops corinnae	TGGCCCCTACAAGTGTTCCTTGAACTTCCCCATTCTGTTT	400
Pseudochirops cupreus	TGGCCCCTACAAGTGTTCCTTCAACTTGCCCGTTCTGTTT	400
Phalanger gymnotis	CAGCTCTGGGAACAGTTCCTTCAACTTTCCCATTCTGTTT	397
Pseudocheirus occidentalis	TGGTCCCTACAAGTATTCCTTCAACTTCCCCATTCTGTTT	399
Pseudocheirus peregrinus	TGGTCCCTACAAGTATTCCTTCAACTTCCCCATTCTGTTT	399
Petauroides volans	TGGCCCCTACAAGTGTTCCTTCAACTTCCTCATTCTGTTT	397
Vombatus ursinus	TGGCCCCGAGAAGCGTTCCTTCAACTTTCCCATTCTGCTT	400

	410 420 430 440	
Petaurus breviceps	TTGGACAATTCCAATTGTTAGAAACTTGGTCCAAATCTAC	439
Acrobates pygmaeus	TTAGATAGCTCCAATCGTTAAAAACTTGGTCCATATCTAC	427
Distoechurus pennatus	TTAGATAGCTCCAATCATTAGAAACTTGGTCCATATCTAC	437
Dactylopsila trivirgata	TTGGATAGCTCCAATTGTTAGAAACTTGCTCCAAATCTAC	440
Macropus eugenii	TTGGATAGTTCCAAATGTTAGAAAGTTGGTCCAAATCTAT	437
Macropus fuliginosus	TTGGATAGTTCCAAATGTTAGAAAGTTGGTCCAAATCTAC	437
Macropus giganteus	TTGGATAGTTCCAAATGTTAGAAAGTTGGTCCAAATCTAC	437
Pseudochirops archeri	TTGGATAGCTCCAATTGTTAGAAACTTGGTCCAAATCTAC	440
Phascolarctos cinereus	TTGGATAGCTCCAAT-------------GTCTAAATCTAC	427
Pseudochirops corinnae	TTGGATAGCTCCAATTGTTAGAAACTTGGTCCAAATCTAC	440
Pseudochirops cupreus	TTGGATAGCTCCAATTGTTAGAAACTTGGTCCAAATCTAC	440
Phalanger gymnotis	TTAGATAATTCCAATTGTTAGAAACTTGGTCCAAATCTAC	437
Pseudocheirus occidentalis	TTGGATAGCTTCAATTGTTAGAAACTTGGTCCAAATCTAC	439
Pseudocheirus peregrinus	TTGGATAGCTTCAATTGTTAGAAACTTGGTCCAAATCTAC	439
Petauroides volans	TTGGATAGCTCCAATTGTTAGAAACTTGGTCCAAATCTAC	437
Vombatus ursinus	TTGGATAGCTCCAATTGTCAGAAACTTGATCTAAATCTAC	440

	450 460 470 480	
Petaurus breviceps	CACTGTACCTTCCACACATTGTTCCTAGTTCTGCCTTTAG	479
Acrobates pygmaeus	CACTGTACCTTCCACACGTTTTCTTAGTTCTTGCCTT---	464
Distoechurus pennatus	CACTGTACCTTCCACACAATGTCCTTAGTTCTGCCTTTGG	477
Dactylopsila trivirgata	CACTGTACTTTCCACACATTGCTCCTAGTTCTGCCTTTAG	480
Macropus eugenii	CACCATACCTTCCACACATCGTTCCTGGTTCTGCCTTTGC	477
Macropus fuliginosus	CACCATACCTTCCACACATTGTTCCTGGTTCTGCCTTTGG	477
Macropus giganteus	CACCATACCTTCCACACATTGTTCCTGGTTCTGCCTTTGG	477
Pseudochirops archeri	CATTGTACCTTCCACGAATTGTTCCTAGTTCTGCCTTTGG	480
Phascolarctos cinereus	CACTATACTTTCCACACATTCTTCCTAGTTCTGCCTTTGG	467
Pseudochirops corinnae	CACTGTACCTTC--CACATTGTTCCTAGTTCTGCCTTTGG	478
Pseudochirops cupreus	CACTGTACCTTCCACGCATTGTTCCTAGTTCTGCCTTTGG	480
Phalanger gymnotis	CACCGTACCTTCCACACATTGTTCCTAGTTCTGCCTTTGG	477
Pseudocheirus occidentalis	CACTGTACCTTCTATACATTGTTCCTAGTTCTGCCTTTGG	479
Pseudocheirus peregrinus	CACTGTACCTTCTATACATTGTTCCTAGTTCTGCCTTTGG	479
Petauroides volans	CACTGTACCTTCCACACATTGTTCCTAGTTCTGCCTTTGG	477
Vombatus ursinus	CACTATACTTTCCACACATTCCTCCTAGTTCTGCCTTTGG	480

	490 500 510 520	
Petaurus breviceps	GGTGAGCAGAATGAATTTAATCTCCCTCCCACCTGACAGC	519
Acrobates pygmaeus	--TGAGCAGAACGAATTTAATCTCCCTCTTCCCTCCCTGA	502
Distoechurus pennatus	GGTGAGCAGAACGAATTTAATCTCCCTCCCACCCCATGAC	517
Dactylopsila trivirgata	GGTGAGCAGAATGAATTTAACCTCCCTCCCACCTGACGAC	520
Macropus eugenii	GGAGAGCAGAACGAATTTAATCTCCCTCCTACCTGAAAGC	517
Macropus fuliginosus	GGAGAGCAGAATGAATTTAATCTCCCTCCTACCTGAAAGC	517
Macropus giganteus	GGAGAGCAGAATGAATTTAATCTCCCTCCTACCTGAAAGC	517
Pseudochirops archeri	GGTGAGCAGAATGAATTTAATCTCCCTCCCACCTGACAGC	520
Phascolarctos cinereus	GGTGAGCAGAACAAATTTAATCTCCCTCCCACCTGACATC	507
Pseudochirops corinnae	GGTGAGCAGAACGAATTTAATCTCCCTCCCACCTGACAGC	518
Pseudochirops cupreus	GGTGAGCAGAATGAATTTAATCTCTCTCCCACCTGACAGC	520
Phalanger gymnotis	GATGAACAGAAGGAATTTAATCTCTCTCCCACCTGACAGC	517
Pseudocheirus occidentalis	GGTGAGCAAAATGAATTTAATCTCCCTCCTACCTCACAGA	519
Pseudocheirus peregrinus	GGTGAGCAAAATGAATTTAATCTCCCTCCTACCTCACAGA	519
Petauroides volans	GGTGAGCAGAATGAATTTAATCTCCCTCCCACATCACAGC	517
Vombatus ursinus	GGTGAGCAGAACAAATTTAATCTCCCTCCTACCTGATATC	520

	530 540 550 560	
Petaurus breviceps	TATCATGTTTTCCAGGAGCCTTCCCTAATTCTTTAAACTA	559
Acrobates pygmaeus	CAGTATGTTTCCCAGGAGCCTTCCCTAATTCCTTAAACCA	542
Distoechurus pennatus	AGCTATGTTTCCCACCAGCCTTCACCAGTTCCTTAAATCA	557
Dactylopsila trivirgata	TGTCATGTTTCCCATGAGCCTTCCCTAATTCTTTAAACTA	560
Macropus eugenii	TATCATGATTCACAGGAGCTTTCCCTAATTCCTTCAGCTA	557
Macropus fuliginosus	CATCATGATTCGCAGGAGCTTTCCCTAATTCCTTCAGCTA	557
Macropus giganteus	CATCATGATTCGCAGGAGCTTTCCCTAATTCCTTCAGCTA	557
Pseudochirops archeri	TATCATGTTCCTCATGAGCCTTCCCTAATTCCTTAAACTA	560
Phascolarctos cinereus	TATCATGTTTCCCAGGAGCCTTCCCTAATTCCCTAAACTA	547
Pseudochirops corinnae	TATCATGTTCCTCATGAGCCTTCCCTAATTCCTTAAACTA	558
Pseudochirops cupreus	TATCATGTTCCTCATGAGCCTTCCCTAATTCCTTAAACTA	560
Phalanger gymnotis	TATCGTGTTTCCCATGAGCCTTCCCTAATTCCTTAAACTA	557
Pseudocheirus occidentalis	TATCATGTTTCTCATGAGCCTTCCCTAATTCCTGCAACTA	559
Pseudocheirus peregrinus	TATCATGTTTCTCATGAGCCTTCCCTAATTCCTTCAACTA	559
Petauroides volans	TATCATGTTTCTCATGAGCCTTCCCTAATTCCTTAAACTA	557
Vombatus ursinus	TATCATGTTTCCCAGGAGCCTTCCCTAATTCCTTAAACTA	560

	570 580 590 600	
Petaurus breviceps	TTTCAAATACATTATGCTCATAGACATTATGTTCTATAGA	599
Acrobates pygmaeus	TTTCAAATACATTATGTTCAGAGACCTTTCTCCATCCTGA	582
Distoechurus pennatus	TTTCAAATACATTATGTTCATAGACCTTTCTTCATTCTGA	597
Dactylopsila trivirgata	TTTCAAATACATTATATTCATAGACATTGTGTTCTATAGA	600
Macropus eugenii	TTTCAAATACATTATGTTCATAGACTTTTCACTATCCTGA	597
Macropus fuliginosus	TTTCAAATACATTATGTTCATAGACTTTTCACTATCCTGA	597
Macropus giganteus	TTTCAAATACATTATGTTCATAGACTTTTCACTATCCTGA	597
Pseudochirops archeri	TTTCAAATACATTATATTCATAGACATTATGTTCTATAGA	600
Phascolarctos cinereus	TTTCAAATACATTAAGTTCGTAGTCCTTTTGCCATCCTGA	587
Pseudochirops corinnae	TTTCAAATACATTATATTCATAGACATTATGTTCTATAGA	598
Pseudochirops cupreus	TTTCAAATACATTATATTCATAGACATTATGTTCTATAGA	600
Phalanger gymnotis	TTTCAAATACATTACATTCATAGGTCTTTTGCCATCCTGA	597
Pseudocheirus occidentalis	TTTCAAATACATTATA---------------TTCTATAGA	584
Pseudocheirus peregrinus	TTTCAAATACATTATA---------------TTCTATAGA	584
Petauroides volans	TTTCAAATATATTATGTTCATAGACATTATGTTCTATAGA	597
Vombatus ursinus	TTTCAAATACACTACGTTCATAGTCCTTTTGCCATCCTGA	600

	610 620 630 640	
Petaurus breviceps	TCACCCTCTTCTTTCCTTGACATTTGTAGAAAATGCACCT	639
Acrobates pygmaeus	TCACCCTCTTCTTTCTTTGACATTGGTGGAAAATACACCT	622
Distoechurus pennatus	TCACCCTCTTCTTTCTTTGACATT----------------	621
Dactylopsila trivirgata	TCACCCTATTCTTTCCTTGACATTTGTAGAAAATGCACCT	640
Macropus eugenii	TCAC---CTTCTTTCCTTGACAGTTGAAGAAAATGCACCT	634
Macropus fuliginosus	TCAC---CTTCTTTACTTGACAGCTGAAAAAAATGCACCT	634
Macropus giganteus	TCAC---CTTCTTTACTTGACAGCTGAAGAAAATGCACCT	634
Pseudochirops archeri	TCACCCTCTTCTTTCCTTGACATATGTTGAAAATGCACCT	640
Phascolarctos cinereus	TCACCCTCTTCTTTCCTTGACATTTGAAGCAAATGCACCT	627
Pseudochirops corinnae	TCACCCTCTTCTTTCCTTGATGTTTGCTGAAAGTGCACCT	638
Pseudochirops cupreus	TCACCCTCTTCTTTCCTTGACATTTGTTGAAAATGCACCT	640
Phalanger gymnotis	TCACCCTCTTCTTTCCTTGACATTTGAAGAAAATGCACAT	637
Pseudocheirus occidentalis	TCACCCTCTTCTTTCCTTGACATTTGTTGAAAATGCGCCT	624
Pseudocheirus peregrinus	TCACCCTCTTCTTTCCTTGACATTTGTTGAAAATGCGCCT	624
Petauroides volans	TCACCCTCTTCTTTCCTTAACATTTATTGAAAATGCACCT	637
Vombatus ursinus	TCACCCTCTTCTTTCCTTGACATTTGAAGCAAATGCACCC	640

	650 660 670 680	
Petaurus breviceps	ATGAGCATGATAAACCTCTAAGAAATATTAACCTAGAGAA	679
Acrobates pygmaeus	ATGAGCATGATTAACCTCTAAGAAGTATTAACGTCGAGAA	662
Distoechurus pennatus	-------TGATAAACCTCTAAGAAATAGTAACCTAGAGAA	654
Dactylopsila trivirgata	ATGAGCATGATAAACCTCTGAGAAAAATTAAACTAGAGAA	680
Macropus eugenii	ATGAGCATGACAAACCTCCAAGAAATACT--CCTAGAGAA	672
Macropus fuliginosus	ATGAGCATGACAAACCTCCAAGAAATACT--CCTAGAGAA	672
Macropus giganteus	ATGAGCATGACAAACCTCCAAGAAATACT--CCTAGAGAA	672
Pseudochirops archeri	ATGAGCACGATAAACCTCTAAGAAATATTAACCTAGAGAA	680
Phascolarctos cinereus	ATGAGCGTGATAAACCTCTACGAAATAATAACTTGGAGAA	667
Pseudochirops corinnae	ATGAGCACAGTAAAGCTCTAAGAAATATTAATTTAGAGAA	678
Pseudochirops cupreus	ATGAGCACAGTAAACCTCTAAGAAATATTAACTTAGAGAA	680
Phalanger gymnotis	ATGAGCATGATAAACCTTTAAGAAATATTAACCTAGAGAA	677
Pseudocheirus occidentalis	ATGAGCATGATAAACCTCCGAGAAATATTAACCTAGAGAA	664
Pseudocheirus peregrinus	ATGAGCATGATAAACCTCCGAGAAATATTAACCTAGAGAA	664
Petauroides volans	ATGAGCACAATAAACCTCTAAGAAATATTAACCTAGAGAA	677
Vombatus ursinus	ATGAGCGTGATAAACCTCCAAGAAATAATAACTTAGAGAA	680

	690	
Petaurus breviceps	ACTGTGTTATT	690
Acrobates pygmaeus	ACTGTA--ATT	671
Distoechurus pennatus	AATGT--CATT	663
Dactylopsila trivirgata	ACTGTGTTATT	691
Macropus eugenii	ACTGTGTCATT	683
Macropus fuliginosus	ACTGTGTCATT	683
Macropus giganteus	ACTGTGTCATT	683
Pseudochirops archeri	ACTGTGTCATT	691
Phascolarctos cinereus	TATGTGTCATT	678
Pseudochirops corinnae	ACTGCATCATT	689
Pseudochirops cupreus	ACTGTGTCATT	691
Phalanger gymnotis	ACTGTGTCATT	688
Pseudocheirus occidentalis	ACTGTGTCATT	675
Pseudocheirus peregrinus	ACTGTGTCATT	675
Petauroides volans	ACTGTGTCATT	688
Vombatus ursinus	GATGTGTCATT	691
